# Whole‐brain microscopy reveals distinct temporal and spatial efficacy of anti‐Aβ therapies

**DOI:** 10.15252/emmm.202216789

**Published:** 2022-11-16

**Authors:** Daniel Kirschenbaum, Ehsan Dadgar‐Kiani, Francesca Catto, Fabian F Voigt, Chiara Trevisan, Oliver Bichsel, Hamid Shirani, K Peter R Nilsson, Karl J Frontzek, Paolo Paganetti, Fritjof Helmchen, Jin Hyung Lee, Adriano Aguzzi

**Affiliations:** ^1^ Institute of Neuropathology University Hospital Zurich University of Zurich Zurich Switzerland; ^2^ Department of Bioengineering Stanford University Stanford CA USA; ^3^ Laboratory of Neural Circuit Dynamics, Brain Research Institute University of Zurich Zurich Switzerland; ^4^ Neuroscience Center Zurich University of Zurich & ETH Zurich Zurich Switzerland; ^5^ Division of Chemistry, Department of Physics, Chemistry and Biology Linköping University Linköping Sweden; ^6^ Laboratory for Biomedical Neurosciences Torricella‐Taverne Neurocenter of Southern Switzerland, Ente Cantonale Ospedaliero Switzerland; ^7^ Faculty of Biomedical Neurosciences Università della Svizzera Italiana Lugano Switzerland; ^8^ Department of Neurology and Neurological Sciences Stanford University Stanford CA USA; ^9^ Department of Electrical Engineering Stanford University Stanford CA USA; ^10^ Department of Neurosurgery Stanford University Stanford CA USA

**Keywords:** Alzheimer's disease, amyloid‐beta, brain, light‐sheet microscopy, tissue clearing, Methods & Resources, Neuroscience

## Abstract

Many efforts targeting amyloid‐β (Aβ) plaques for the treatment of Alzheimer's Disease thus far have resulted in failures during clinical trials. Regional and temporal heterogeneity of efficacy and dependence on plaque maturity may have contributed to these disappointing outcomes. In this study, we mapped the regional and temporal specificity of various anti‐Aβ treatments through high‐resolution light‐sheet imaging of electrophoretically cleared brains. We assessed the effect on amyloid plaque formation and growth in Thy1‐APP/PS1 mice subjected to β‐secretase inhibitors, polythiophenes, or anti‐Aβ antibodies. Each treatment showed unique spatiotemporal Aβ clearance, with polythiophenes emerging as a potent anti‐Aβ compound. Furthermore, aligning with a spatial‐transcriptomic atlas revealed transcripts that correlate with the efficacy of each Aβ therapy. As observed in this study, there is a striking dependence of specific treatments on the location and maturity of Aβ plaques. This may also contribute to the clinical trial failures of Aβ‐therapies, suggesting that combinatorial regimens may be significantly more effective in clearing amyloid deposition.

## Graphical abstract

The brain is highly compartmentalized with many distinct regions. It is unknown how drugs for treating Alzheimer's Disease (AD) work across brain regions and disease stages. We developed a technology to quantify the effects of different AD drugs throughout the brain at different time points.

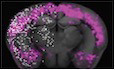

## Introduction

Pathological protein aggregations typically occur in distinct neuroanatomical locations and give rise to specific clinical pictures (Lau *et al*, 2020; Shahnawaz *et al*, 2020), yet the determinants of this specificity are poorly understood. In Alzheimer's Disease (AD), the most prevalent neurodegenerative disorder (Fiest *et al*, 2016), deposition of amyloid‐β (Aβ) plaques occurs in a well‐characterized sequence (Braak *et al*, 1993; Hardy & Selkoe, 2002; Thal *et al*, 2002). This plaque load can be effectively reduced by either quenching Aβ production (De Strooper *et al*, 2010), reducing the propagation of Aβ aggregates (Jiang *et al*, 2013), or enhancing Aβ catabolism (Sevigny *et al*, 2016). However, the clinical efficacy of Aβ removal is still debated (Morris *et al*, 2018; Howard & Liu, 2020), perhaps because intervention is too late to be efficacious (Sperling *et al*, 2013). It is also conceivable that anti‐Aβ drugs remove plaques differentially in distinct CNS regions, some of which may not coincide with the areas that matter most to proper brain functioning.

To investigate this latter hypothesis, we developed a high‐throughput quantitative 3D histology (Q3D) platform for optically clarifying, staining, imaging, and quantifying Aβ plaques in whole brains of mice. Aβ plaques were electrophoretically stained in cleared brains and imaged with a mesoscale selective plane illumination microscope (mesoSPIM; Voigt *et al*, 2019). In APP/PS1 mice (Radde *et al*, 2006), we tested the effects of the polythiophene LIN5044, which intercalates with amyloids and is therapeutic in prion diseases (Margalith *et al*, 2012; Herrmann *et al*, 2015), the BACE1 inhibitor NB360 (Neumann *et al*, 2015, 2019), and a β1‐antibody (Paganetti & Schmitz, 1996). We found that each drug had differential efficacy on plaque formation, plaque growth, and plaque maturity, as well as a striking spatiotemporal dependence. We further found that the two most effective treatments for reducing plaque growth, BACE1 and LIN5044, acted onto distinct, largely non‐overlapping brain regions. Finally, the alignment of whole‐brain treatment maps to a spatial transcriptomics atlas allowed us to identify transcriptional signatures correlating with the effectiveness of each drug.

## Results

### Rapid tissue clearing and staining platform

**Figure 1:**
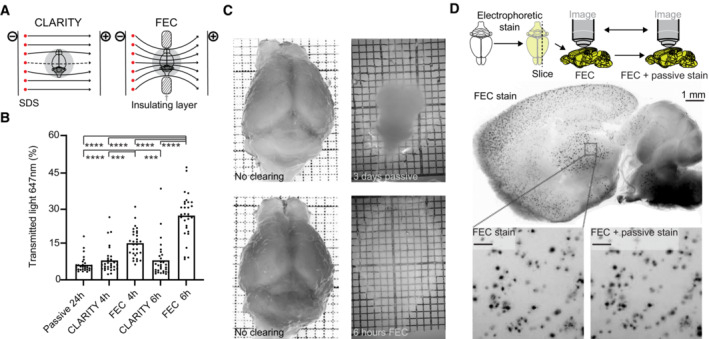
Focused electrophoresis improves tissue clearing efficiency Schematics of both CLARITY and focused electrophoretic clearing (FEC). An insulating layer constrains the electrical field through the tissue.Light transmittance of tissue increased more rapidly with FEC than with CLARITY. Each datapoint plotted is the mean from three neighboring transmittance readings (hence resulting in 10 datapoints from the 30 measurements/brain, technical replicates; One‐way ANOVA ***P < 0.001, ****P < 0.0001; *n* = 3 per group, biological replicates).Comparison of mouse brains cleared by FEC (6 h) and passive (3 days). Passive clearing was incomplete even after 3 days.After whole‐brain electrophoretic staining with polythiophenes, sagittal 500 μm slices were cut and plaques were counted. By passively re‐staining the same slice, no additional plaques were detected (“FEC + passive”). Focal shifts after slice reprocessing account for the slight differences between the images. One biological replicate. Scale bars: 100 μm. Source data are available online for this figure.

Detergent‐mediated lipid extraction from hydrogel‐embedded tissues is facilitated by electrophoretic mobilization of detergent molecules (Chung *et al*, 2013; Tomer *et al*, 2014) in a buffer‐filled container. However, the electrical resistivity of 4% paraformaldehyde‐fixed PBS‐soaked brain tissue is fourfold higher than that of PBS (Appendix Fig S1E). Therefore, any buffer surrounding the specimen short‐circuits its electrophoresis. We resolved this issue by constructing a focused electrophoretic clearing (FEC) device that uncouples buffer recirculation in the anodic and cathodic circuits with an insulating layer (Fig 1A; Appendix Fig S1). By forcing the electrical current to traverse the tissue specimen (130 mA in constant‐current, 39.5°C), FEC reduced the clearing time from 48 to 120 with CLARITY to 6–14 h (Chung *et al*, 2013, Tomer *et al*, 2014) and resulted in homogeneous high‐quality clearing (Fig 1B and C; Appendix Fig S1G and H). Similar to other hydrogel‐based clearing methods, brain tissue showed some swelling during clearing, which was reversible upon PBS washing and refractive‐index matching.

For Aβ plaque staining of intact mouse brains by electrophoresis, we constructed buffer‐filled chambers hosting the electrodes. Their inner faces were cast with 10% polyacrylamide in tris‐tricine buffer and functioned as electrically conductive contact surfaces. The sample was mounted with a holder between the two polyacrylamide walls. This allowed electrophoresis to occur through the buffers, the gels, and the tissue specimen; spanning 10 cm between the two electrodes (Appendix Fig S2A and B). The electric resistance of the electrophoretic system (20 V, 20°C) increased from initially ~ 2 to ~ 20 kΩ after 2 h (Appendix Fig S2C).

We then ran native‐gel electrophoreses of proteins with various charges at different pH and ionic strengths. Tris‐tricine (50 mM each) at pH 8.5 yielded the best results (Appendix Fig S2D–G). As expected, the electrophoretic mobility of proteins was influenced by the charge of covalently coupled fluorophores (Appendix Fig S2D–G). The polythiophenes qFTAA (616.5 g/mol, m/z = 205.5) and hFTAA (948.9 g/mol, m/z = 237.125) were dissolved in agarose (600 μl, congealing temperature 26–30°C) and cast on the acrylamide‐tissue interface in order to confine them to the smallest possible volume. Under these conditions, the dye front traversed the entire brain within 2 h.

An uneven passage of the dye front through the brain may lead to local inhomogeneities of plaque detection, particularly at gel–liquid interfaces. To investigate this question, a hydrogel‐embedded and cleared APP/PS1 brain was electrophoretically stained with polythiophenes (2 h) and cut into 500‐μm sagittal sections with a vibratome. Free‐floating sections were imaged with a fluorescent stereomicroscope. Then, the sections were passively re‐stained with the same polythiophene dyes using a well‐established protocol (Nystrom *et al*, 2013; Rasmussen *et al*, 2017), and images were acquired again. The numbers of Aβ plaques were 3,085 and 3,061 plaques before and after re‐staining, respectively, and their morphology was very similar (Fig 1D; Appendix Fig S3). Hence, the sensitivity and spatial homogeneity of electrophoretic plaque staining of whole brains were not inferior to that of conventional histochemical slice staining. Slight differences in plaque counts and morphology were a result of physical distortions of the slices during passive staining and due to focal shifts during re‐imaging of the free‐floating slices.

**Figure EV1:**
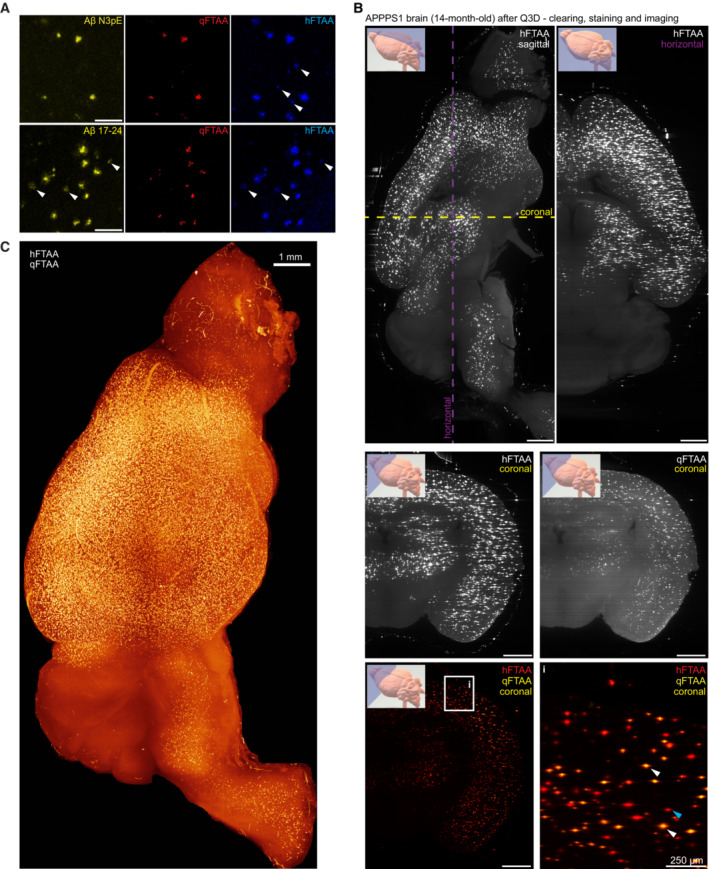
Homogeneous whole‐brain Aβ plaque stain by electrophoretic infusion of qFTAA and hFTAA polythiophenes Plaque maturation states determined with qFTAA vs hFTAA were compared to those identified by N3pE (pyroglutamylated Aβ) vs Aβ_17‐24_ (all Aβ moieties). APPPS1 brain sections from one mouse (paraffin, 10 μm) were stained with Aβ‐N3pE or Aβ17 24 followed by qFTAA+hFTAA staining. Aβ‐N3pE detected plaques similarly to qFTAA, while hFTAA highlighted additional plaques. Conversely, Aβ_17‐24_ labeled the entire plaque population similarly to hFTAA (white arrowheads). Scale bars: 100 μm.Lightsheet imaging and digital reslicing of cleared whole brains. Plaques were visible in the cortex and in deep diencephalic areas, indicative of homogeneous dye penetration and image acquisition. Plaque maturity was assessed with qFTAA+hFTAA co‐staining. As an example, hFTAA identified all plaques (blue arrows), whereas qFTAA stained the cores (white arrows) of more mature plaques. Scale bars: 1 mm.Whole‐brain hemisphere rendering of an APPPS1 mouse with hFTAA signal. The cerebellum, where the Thy1 promoter is inactive, was unaffected. Source data are available online for this figure.

Antibody Aβ17‐24 recognizes early plaques and stains their entire surface, whereas N3pE labels plaques that accumulate at later stages (Rijal Upadhaya *et al*, 2014). Likewise, the polythiophene hFTAA stains the entire area of early plaques, whereas qFTAA stains the cores of plaques in older mice. The qFTAA/hFTAA ratio correlates with plaque compactness (Nystrom *et al*, 2013) and is used as a proxy for their maturity. We stained histological sections (3 μm) from paraffin‐embedded APP/PS1 brains with Aβ17‐24 or N3pE, followed by staining with qFTAA and hFTAA. The hFTAA and Aβ17‐24 signals were largely superimposable and identified more plaques than qFTAA and N3pE, which stained selectively the cores of a subset of plaques (Fig EV1A). Most Aβ17‐24^+^ N3pE^−^ plaques were hFTAA^+^ qFTAA^−^, suggesting that they contained less mature amyloid. We conclude that the qFTAA/hFTAA stain is a good proxy to plaque maturity and suitable for whole‐brain staining (Fig EV1B and C).

### Evaluation of anti‐Aβ therapies by Q3D

**Figure 2:**
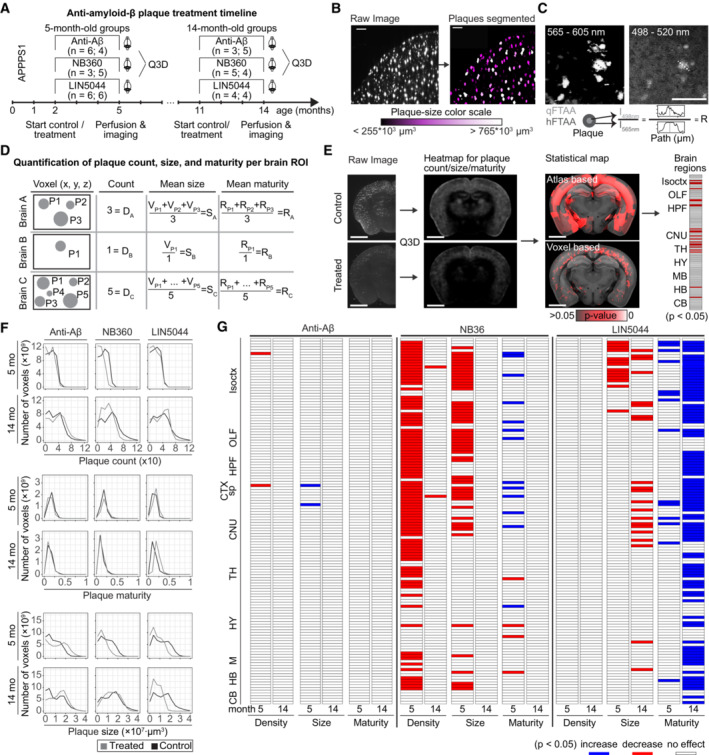
Region and age‐specific plaque clearing by various anti‐Aβ treatments APPPS1 mice were treated with antibodies, the BACE1 inhibitor NB360, the polythiophene LIN5044 or appropriate controls.We segmented the plaques and color‐coded them by plaque size (μm^3^). Scale bars: 250 μm.Intensity ratios were calculated by dividing the peak fluorescent emission in the qFTAA channel with the peak emission of the hFTAA channel in the center of each plaque. Scale bar: 100 μm.Descriptive statistics were calculated for every voxel of individual brains resulting in plaque counts, mean plaque size, and mean intensity ratio for each voxel (plaques: P1, P2 … Pn).After every brain was registered to a reference atlas, plaques were grouped by anatomical brain regions. The mean plaque density, size, and maturity was compared between control and treated brains for corresponding brain regions. Seeking an unbiased volume unit for spatial analysis we divided brain data into (25 μm)^3^ voxels. Atlas‐based anatomical normalization overestimates the volume of treatment‐affected brain compared to voxel‐level analysis. Scale bars: 2 mm.Histograms visualizing the changes in plaque size and maturity upon different treatments. For the β1 antibody treatment, there was a reduction in the number of smaller plaques, but there was no change in plaque density or maturity. NB360 and LIN5044 reduced the prevalence of large plaques in young mice; LIN5044 also reduced it in old mice. NB360 affected the plaque maturity of young but not of old mice, while LIN5044 increased plaque maturity primarily in old mice.Statistical tests resulted in heatmaps of significance by anatomical brain regions. The β1 antibody treatment showed very limited effects in all analyzed brain regions and across all analyzed metrics. NB360 reduced plaque density and mean size in brain regions in 5‐month but not in 14‐month‐old mice. Mean plaque‐core maturity increased in cortex but decreased in several subcortical structures. LIN5044 was effective in reducing mean plaque size and maturity in many brain areas at 14 months, and on mostly cortical areas at 5 months. Isoctx, isocortex; OLF, olfactory areas; HPF, hippocampal formation; CTX sp, cortical subplate; CNU, caudate nucleus; TH, thalamus; HY, hypothalamus; MB, midbrain; HB, hindbrain; CB, cerebellum.

Groups of 2‐month old or 11‐month old APP/PS1 mice (30 and 25 mice/group, henceforth referred to as “young” and “old,” respectively) were treated for 90 days with the BACE1 inhibitor NB360 (0.5 g inhibitor/kg chow, ~ 3 mg inhibitor/day/mouse), with β1 antibody against Aβ (0.5 mg in 200 μl PBS), 1×/week intraperitoneally, based on previous protocols (Pfeifer *et al*, 2002, Balakrishnan *et al*, 2015), or with the amyloid‐binding compound LIN5044 (0.4 mg in 100 μl PBS, 1×/week intraperitoneally; Table 1). Control treatments included control food chow and intraperitoneally injected recombinant pooled IgG or PBS, respectively (Fig 2A). Mice were sacrificed 1 week after the last administration of LIN5044 or β1; the NB360 chow was provided without interruption. Brains were subjected to clearing, staining, and imaging. Raw data volumes were transformed to the coordinate space of the Allen Brain Atlas (Wang *et al*, 2020) and anatomically registered (Appendix Fig S4A, D, and E). We then performed automated plaque segmentation and regional quantification of plaque pathology (Fig. 2B; Appendix Fig S4A–C; Table 2). Voxel‐level plaque counts, mean size, and maturity (qFTAA/hFTAA ratio) were determined for each treatment group (Fig 2C and D; Appendix Fig S5; Table 3). Corresponding voxels of brains treated with anti‐Aβ compounds and their respective controls were compared pairwise by inferential statistics (Fig 2E). This allowed us to identify “Significantly Altered Voxels” (SAV) across entire brain volumes. SAV heatmaps were presented as montages of coronal slices.

**Table 1:** Number of mice in treatment cohorts.

	β1‐old	β1‐young	NB360‐old	NB360‐young	LIN5044‐old	LIN5044‐young
Control	5	4	4	5	4	6
Treated	3	6	5	3	4	6

**Table 2:** Neuroanatomical areas used for Allen Reference Atlas registration.

Region acronym	Region name
FRP	Frontal pole, cerebral cortex
MOp	Primary motor area
MOs	Secondary motor area
SSp	Primary somatosensory area
SSs	Supplemental somatosensory area
GU	Gustatory areas
VISC	Visceral area
AUDd	Dorsal auditory area
AUDp	Primary auditory area
AUDpo	Posterior auditory area
AUDv	Ventral auditory area
VISal	Anterolateral visual area
VISam	Anteromedial visual area
VISl	Lateral visual area
VISp	Primary visual area
VISpl	Posterolateral visual area
VISpm	posteromedial visual area
VISa	Anterior area
VISli	Laterointermediate area
ACA	Anterior cingulate area
PL	Prelimbic area
ILA	Infralimbic area
ORB	Orbital area
AI	Agranular insular area
RSP	Retrosplenial area
VISpor	Postrhinal area
VISrl	Rostrolateral visual area
TEa	Temporal association areas
PERI	Perirhinal area
ECT	Ectorhinal area
OLF	Olfactory areas
MOB	Main olfactory bulb
AOB	Accessory olfactory bulb
AON	Anterior olfactory nucleus
TT	Taenia tecta
DP	Dorsal peduncular area
PIR	Piriform area
NLOT	Nucleus of the lateral olfactory tract
COA	Cortical amygdalar area
PAA	Piriform‐amygdalar area
TR	Postpiriform transition area
HPF	Hippocampal formation
HIP	Hippocampal region
ENTl	Entorhinal area, lateral part
ENTm	Entorhinal area, medial part, dorsal zone
PAR	Parasubiculum
POST	Postsubiculum
PRE	Presubiculum
SUB	Subiculum
ProS	Prosubiculum
HATA	Hippocampo‐amygdalar transition area
APr	Area prostriata
CTXsp	Cortical subplate
CLA	Claustrum
EP	Endopiriform nucleus
LA	Lateral amygdalar nucleus
BLA	Basolateral amygdalar nucleus
BMA	Basomedial amygdalar nucleus
PA	Posterior amygdalar nucleus
STR	Striatum
CP	Caudoputamen
ACB	Nucleus accumbens
FS	Fundus of striatum
OT	Olfactory tubercle
LSX	Lateral septal complex
AAA	Anterior amygdalar area
BA	Bed nucleus of the accessory olfactory tract
CEA	Central amygdalar nucleus
IA	Intercalated amygdalar nucleus
MEA	Medial amygdalar nucleus
PAL	Pallidum
GPe	Globus pallidus, external segment
GPi	Globus pallidus, internal segment
SI	Substantia innominata
MA	Magnocellular nucleus
MSC	Medial septal complex
TRS	Triangular nucleus of septum
PALc	Pallidum, caudal region
TH	Thalamus
VENT	Ventral group of the dorsal thalamus
SPF	Subparafascicular nucleus
SPA	Subparafascicular area
PP	Peripeduncular nucleus
GENd	Geniculate group, dorsal thalamus
LAT	Lateral group of the dorsal thalamus
ATN	Anterior group of the dorsal thalamus
MED	Medial group of the dorsal thalamus
MTN	Midline group of the dorsal thalamus
ILM	Intralaminar nuclei of the dorsal thalamus
RT	Reticular nucleus of the thalamus
GENv	Geniculate group, ventral thalamus
MH	Medial habenula
LH	Lateral habenula
HY	Hypothalamus
PVZ	Periventricular zone
PVR	Periventricular region
AHN	Anterior hypothalamic nucleus
MBO	Mammillary body
MPN	Medial preoptic nucleus
PMd	Dorsal premammillary nucleus
PMv	Ventral premammillary nucleus
PVHd	Paraventricular hypothalamic nucleus, descending division
VMH	Ventromedial hypothalamic nucleus
PH	Posterior hypothalamic nucleus
LHA	Lateral hypothalamic area
LPO	Lateral preoptic area
PST	Preparasubthalamic nucleus
PSTN	Parasubthalamic nucleus
PeF	Perifornical nucleus
RCH	Retrochiasmatic area
STN	Subthalamic nucleus
TU	Tuberal nucleus
ZI	Zona incerta
ME	Median eminence
MB	Midbrain
MBsen	Midbrain, sensory related
MBmot	Midbrain, motor related
MBsta	Midbrain, behavioral state related
P	Pons
NLL	Nucleus of the lateral lemniscus
PSV	Principal sensory nucleus of the trigeminal
PB	Parabrachial nucleus
SOC	Superior olivary complex
P‐mot	Pons, motor related
P‐sat	Pons, behavioral state related
MY	Medulla
MY‐sen	Medulla, sensory related
MY‐mot	Medulla, motor related
MY‐sat	Medulla, behavioral state related
CB	Cerebellum
VERM	Vermal regions
HEM	Hemispheric regions
CBN	Cerebellar nuclei

**Table 3:** Plaque loads in control mice of each treatment cohort show low variability.

Treatment	Age	Count Mean	Count SD	Count SE
Antibody	Old	2,108,735	292,186	130,670
Antibody	Young	1,097,711	159,214	79,607
BACE1 inhibitor	Old	2,790,290	245,900	122,950
BACE1 inhibitor	Young	1,151,097	159,849	79,924
Polythiophene	Old	2,738,744	333,892	166,946
Polythiophene	Young	1,243,608	156,807	64,016
<all>	Old	2,512,293	426,799	118,373
<all>	Young	1,175,491	159,340	42,585

### Local efficacy of therapies with neuroanatomical areas

**Figure EV2:**
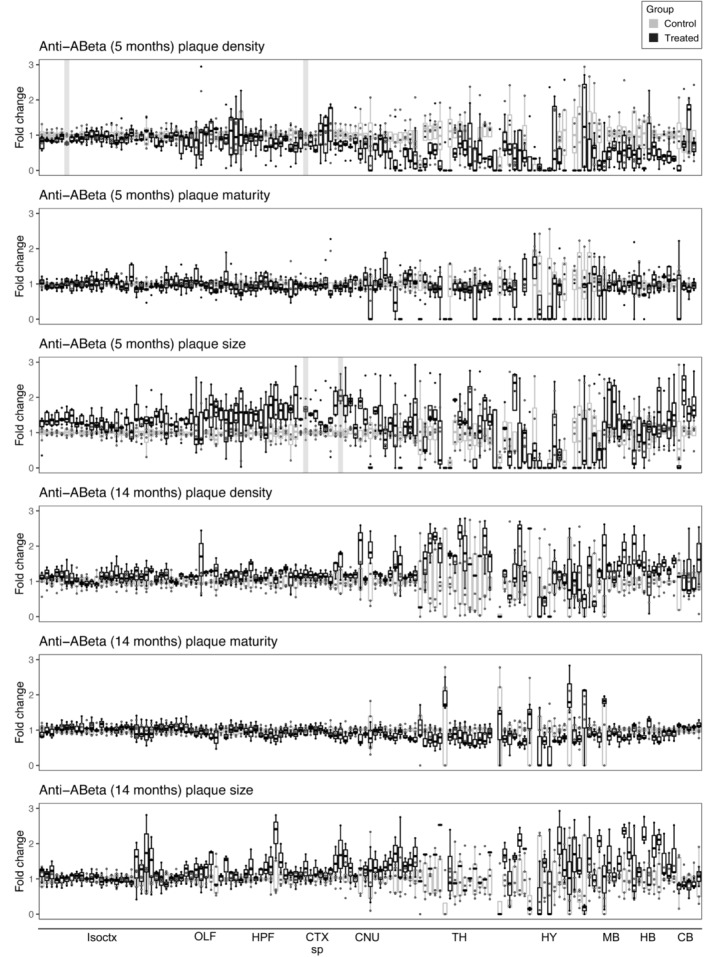
The β1 antibody has minimal to no effect on neuroanatomical plaque count, maturity, or size in 5‐month‐old and 14‐month‐old mice Fold‐change reduction in various plaque metrics across all brain regions in both 5‐month‐old and 14‐month‐old mice, compared with control. The β1 antibody has a minimal effect on plaque‐count increase in 5‐month‐old mice and has no effect in old mice. In 5‐month‐old mice, significant effects in plaque size reduction occurred mostly in the brainstem. The plaque‐maturity change induced by β1 antibody treatment in 5‐ and 14‐month‐old mice is very limited. The plaque‐size change induced by β1 antibody treatment in 5‐month‐old mice is very limited and absent in 14‐month‐old mice. The plaque‐maturity change induced by β1 antibody treatment in 5‐ and 14‐month‐old mice is very limited. Brain regions with a significant treatment effect (*P* < 0.05) are shaded gray. Isoctx, isocortex; OLF, olfactory areas; HPF, hippocampal formation; CTX sp, cortical subplate; CNU, caudate nucleus; TH, thalamus; HY, hypothalamus; MB, midbrain; HB, hindbrain; CB, cerebellum.

**Figure EV3:**
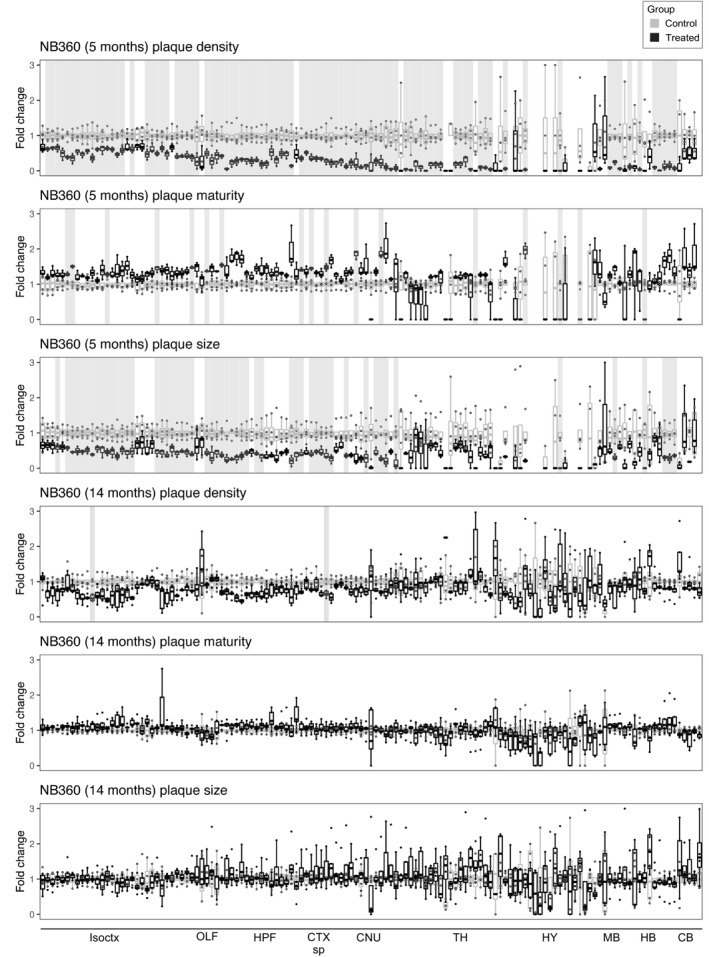
BACE1‐inhibition induced conspicuous plaque count reduction in 5‐month‐old mice, but not in 14‐month‐old mice Fold‐change reduction in various plaque metrics across all brain regions in both 5‐month‐old and 14‐month‐old mice. BACE1‐inhibition induces considerable reduction in both plaque count and plaque size in many anatomical regions in 5‐month‐old mice, but not in 14‐month‐old mice. Plaque maturity change by BACE1‐inhibition in 5‐month‐old mice shows both region‐dependent maturity increase and decrease, while there is no effect at 14‐months. Brain regions with a significant treatment effect (*P* < 0.05) are shaded gray. Isoctx, isocortex; OLF, olfactory areas; HPF, hippocampal formation; CTX sp, cortical subplate; CNU, caudate nucleus; TH, thalamus; HY, hypothalamus; MB, midbrain; HB, hindbrain; CB, cerebellum.

The effects of the β1 antibody were surprisingly small. In young mice, the increase in plaque density was slightly reduced (gustatory areas and claustrum: *P* = 0.014), whereas size was marginally increased (claustrum: *P* = 0.044), and plaque maturity was unaffected. In old mice there was no significant effect (Figs 2G and EV2; Appendix Fig S6). In contrast, NB360 robustly quenched the increase in plaque density and (to a lesser extent) size in 5‐month‐old mice. The effect on plaque density was most pronounced in subcortical areas (claustrum: *P* = 0.004) and in ventral and posterior cortical areas including the perirhinal and posterolateral visual area (both *P* = 0.004), whereas the effect on plaque size was particularly strong in the amygdala and piriform area (both *P* = 0.024; Figs 2F and G and EV3). Plaque maturity (based on the qFTAA/hFTAA fluorescent ratio) was increased in superficial cortical areas (e.g., olfactory areas *P* = 0.011) but decreased in deep subcortical structures (e.g., amygdala *P* = 0.017). In old mice, NB360 had no significant effect on plaque density, size, and maturity.

**Figure EV4:**
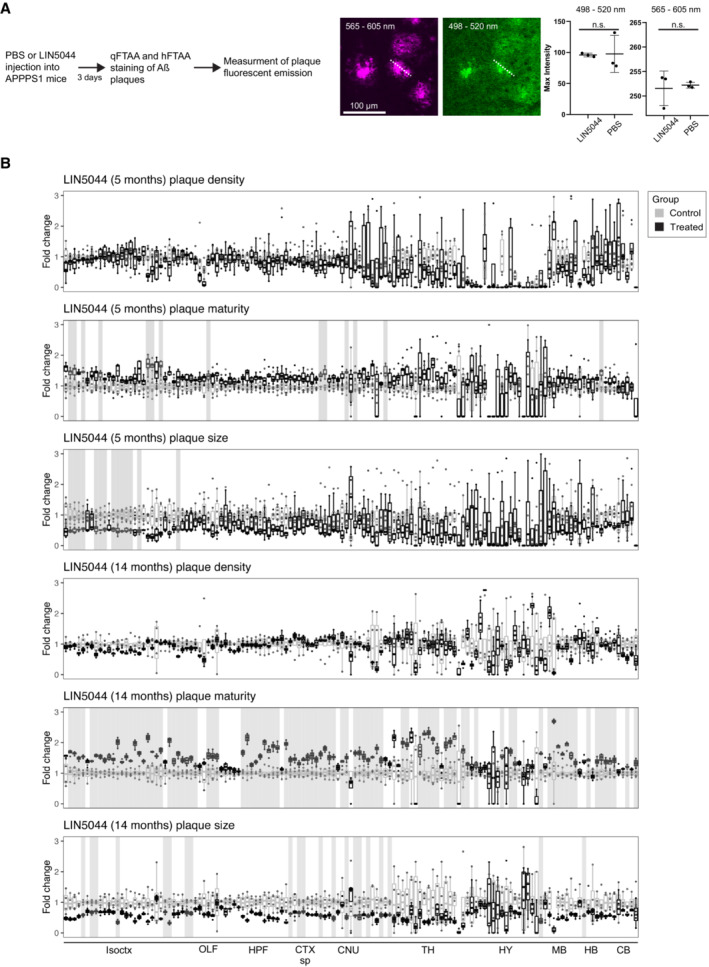
Trend in plaque‐count reduction in few anatomical regions after LIN5044 treatment in 5‐ and 14‐month‐old mice To see whether LIN5044 induces artifacts into the plaque‐maturity analysis APPPS1 mice were injected with a single‐dose PBS (*n* = 1) or LIN5044 (0.4 mg in 100 μl PBS; *n* = 1). Next, emission intensities used for plaque maturity analysis (at wave lengths 498–520 and 565–605 nm) were measured in 10 plaques in 3 slices per animal with a confocal microscope. Plaques of LIN5044 and PBS injected mice showed no difference in fluorescent emissions.Fold‐change reduction in various plaque metrics across all brain regions in both 5‐month‐old and 14‐month‐old mice, compared with control. Plaque‐count reduction is not significant but shows a trend in 14‐month‐old mice. Mean plaque‐sizes are significantly reduced in some anatomical regions after LIN5044 treatment in 5‐month‐old mice, and in cortical areas of 14‐month‐old mice. Plaque maturity change by LIN5044 in 5‐month‐old mice shows some neuroanatomical regions with mean maturity increase, and a more widespread regional increase at 14‐months in cortical areas. Brain regions with a significant treatment effect (*P* < 0.05) are shaded gray. Isoctx, isocortex; OLF, olfactory areas; HPF, hippocampal formation; CTX sp, cortical subplate; CNU, caudate nucleus; TH, thalamus; HY, hypothalamus; MB, midbrain; HB, hindbrain; CB, cerebellum.

LIN5044 acted primarily on plaque size, but only marginally on plaque density of old mice (Figs 2F and G and EV4B). Plaques were smaller in subcortical areas (medial septal complex and amygdala: *P* = 0.0048 and 0.012 respectively) and cortical areas with a rostro‐dorsal emphasis (supplemental somatosensory area: *P* = 0.0067). The effect of LIN5044 on mean plaque size was more pronounced in old mice, but the spatial distribution of the treatment effect was similar in young and old mice (Figs 2F and G, and EV4B). In contrast, the effect of LIN5044 on plaque density in young mice was less conspicuous.

LIN5044 treatment may influence the fluorescent spectra of plaques and distort maturity analyses. We therefore measured plaque spectra of APP/PS1 mice 3 days after a single injection of LIN5044 or PBS. The emission spectra of plaques were not influenced (Fig EV4A). In contrast, the cohorts treated for 3 months with LIN5044 showed a massive shift toward increased plaque maturity in old mice (retrosplenial area: *P* = 0.0014) and to a lesser extent in young mice (supplemental somatosensory area: *P* = 0.026; Figs 2G and EV4B).

### Regional drug – Effect analysis based on voxel‐level probability distribution

**Figure 3:**
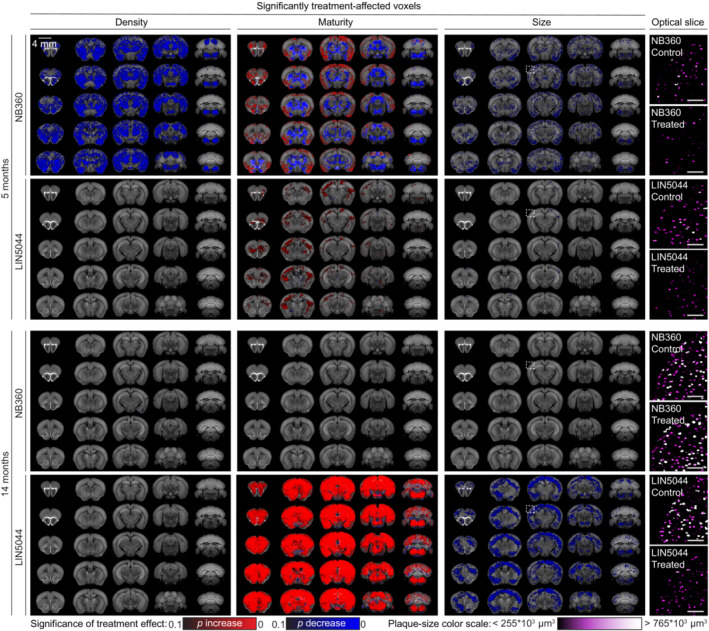
Voxel‐based brain analysis reveals temporally distinct anti‐Aβ treatment effects across various plaque metrics Each three‐dimensional map of SAVs summarizes all the treated and control samples within a cohort (8–12 samples). These maps reveal regiospecific efficacy unique to each treatment modality. NB360 reduced plaque count and mean size in both cortical and subcortical areas of young (5‐month) mice, with little to no effect in older mice (14‐month). NB360 also showed profoundly divergent effects on mean plaque maturity between cortical and subcortical regions, which showed increase and decrease, respectively. In older mice, LIN5044 treatment showed a widespread increase in mean plaque maturity, and a significant reduction in mean plaque size across the whole brain. The mean plaque size reduction suggested by the voxel maps was confirmed by looking at segmentations of randomly picked cortical optical slices (white inserts on heatmaps), color‐coded by plaque size. Scale bars: 500 μm.

We decomposed atlas‐registered brains into spatially registered cubic voxels (15,625 μm^3^) and generated descriptive statistics of plaque density, size, and maturity for each voxel. We then assessed the effects of each treatment arm at the single‐voxel level (Fig 3; Appendix Figs S4 and S5). We found that the locales of treatment effectiveness did not coincide with neuroanatomically defined regions. Indeed, assignment by neuroanatomical boundaries failed to capture peaks of regiospecific therapeutic efficacy and overestimated the volume of treatment‐affected brain tissue (Fig 2E). Remarkably, voxel‐level heatmaps of *P*‐values showed that BACE1 inhibition reduced the increase in plaque counts most effectively in the posterior and ventral telencephalon (Fig 3), whereas LIN5044 reduced the increase in the size of plaques primarily in rostro‐dorsal areas (Fig 3). These effects were symmetric across the midline and showed sharp boundaries lining the deep cortical layers (LIN5044), the thalamus, and CA3 (NB360). The β1‐treated young mice showed patchy reduction in plaque count and size in the brainstem and some decrease in maturity (Fig EV2; Appendix Fig S6). However, these effects were marginal. The effects of β1 in old mice were even less significant (Fig EV2; Appendix Fig S6). Therefore, β1 was excluded from further analyses.

The inferred effects of NB360 and LIN5044 rely on complex computations on terabyte‐sized datasets. To intuitively visualize these effects, we randomly selected single cortical mesoSPIM images of atlas‐registered brains from each treatment and control groups. Upon segmentation, we color‐coded plaques based on their size (Fig 2B; Appendix Fig S7). Fig 3 and Appendix Fig S7 confirm the reduced plaque density and plaque size in NB360‐treated young mice and LIN5044‐treated old mice, respectively.

### Colocalization analysis reveals little overlap in the regiospecificity of therapies

**Figure 4:**
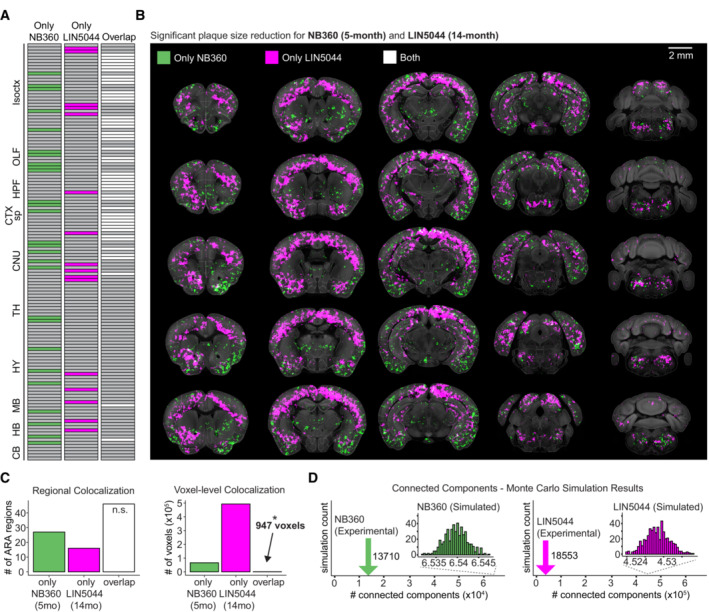
Colocalization of LIN5044 and NB360 voxel‐based analysis reveals spatially distinct anti‐Aβ treatment effect on plaque size A, BThe distinct significant effect of each of the strongest treatments on plaque size cluster, NB360 5‐month and LIN5044 14‐month, as well as their overlap, using either (A) neuroanatomical boundaries, or (B) SAVs.CThe two treatments demonstrate high colocalization based on neuroanatomical boundaries, while the SAVs for plaque‐size cluster separately with limited overlap (*P = 0.03; hypergeometric test: 32403808 total voxels, 66,128 NB360 voxels, 492,779 LIN5044 voxels, 947 overlapping voxels).DTo demonstrate that these individual SAV‐clusters are non‐random, Monte‐Carlo simulations were run, which randomly distributed the experimentally measured number of voxels while the number of connected components was counted. LIN5044 and NB360 SAVs both show at least fivefold higher clustering than random.

As a global measure of regiospecific similarity, we counted the overlapping SAVs in all treatment pairs and metrics (plaque density, mean plaque size, and maturity; Table 4). Despite a strong colocalization within neuroanatomical boundaries, the LIN5044 and NB360 SAVs appeared to cluster in distinct patterns (Fig 4A). The maximal SAV overlap between pairs was < 1% or < 2.65% (*P* < 0.05 or *P* < 0.1, respectively) indicating that each treatment had a unique voxel‐specific fingerprint (Fig 4A–C, Table 4). Hypergeometric tests confirmed that the voxel‐level overlaps were not significant (*P* < 0.03).

**Table 4:** Significantly affected voxels (SAV) after NB360, β1‐antibody or LIN5044 treatment.

	Young			Old		
	NB360	β1	LIN5044	NB360	β1	LIN5044
***P* < 0.05**						
Density						
NB360	2,649,191	435	2	46	0	0
β1	435	4,625	0	0	40	0
LIN5044	2	0	29	0	0	276
Size						
NB360	66,128	3	1	10	0	0
β1	3	2,518	0	0	18	1
LIN5044	1	0	80	0	1	492,779
Maturity						
NB360	1,593,392	9,542		136	0	
β1	9,542	52,893		0	639	
***P* < 0.1**						
Density						
NB360	7,185,540	5,443	30	16,304	0	0
β1	5,443	21,359	0	0	69	0
LIN5044	30	0	121	0	0	486
Size						
NB360	888,437	282	1,302	25	0	0
β1	282	8,661	1	0	61	5
LIN5044	1,302	1	20,530	0	5	2,941,884
Maturity						
NB360	2,181,488	19,777		221	0	
β1	19,777	84,862		0	1,432	

After all the brain scans were registered to a brain atlas, the brains were deconstructed into standard voxels in a coordinate system. Then, for every treatment a “phantom” brain‐volume was generated where each voxel represented the *P*‐value of treatment effect. This was generated by two‐sided *t*‐testing all treated against all control brains (for a respective voxel). Voxels with *P*‐values either *P* < 0.1 or 0.05 were termed as SAV. Each treatment's phantom brain was thresholded to only contain SAVs (*P* < 0.1 or 0.05). The effect‐overlap between treatments was defined by the voxels, which were significantly affected in both of the compared treatments. Our results show that most SAVs are non‐overlapping. The overlap was <1% or <2.65% at *P* < 0.05 or *P* < 0.1, respectively.

To probe the randomness of such clusters, we measured the number of connected components (neighboring SAVs that are touching each other) in NB360 and LIN5044‐treated brains (13,710 and 18,553, respectively). We then generated 500 Monte Carlo simulations with the same number of SAVs than we measured experimentally, but without constraints on their spatial distribution. We found that the number of connected components in the experimental measurements was at least fivefold lower than in the simulations, indicating that the SAVs are grouped into distinct, spatially confined clusters (Fig 4D).

### Regiospecificity is not due to pharmacokinetics or Aβ and BACE1 levels

To test if the regiospecificity was caused by differential penetration of therapeutic compounds, we determined the biodistribution of β1, NB360, and LIN5044 by dissecting brains into eight standard regions (Appendix Fig S8A). NB360 levels were measured 1 h after oral administration (Neumann *et al*, 2015). Since antibodies have long half‐lives (Vieira & Rajewsky, 1988) and limited blood–brain barrier penetration, brain levels were measured 6 and 24 h after intraperitoneal injection. As the pharmacokinetic properties of LIN5044 are unknown, we measured brain levels 2 and 6 h after intraperitoneal administration. There was no difference in regional NB360 levels. LIN5044‐treated brains showed higher levels in the brainstem and cerebellum (*P* < 0.034 and 0.024, respectively) after 2 h, but its distribution became homogeneous after 6 h (Appendix Fig S8B–D). β1 showed higher levels in the brainstem after 6 and in the brainstem and the cerebellum after 24 h (Appendix Fig S8E and F). However, there was no difference in antibody levels between diencephalic and telencephalic regions. Pooled nonspecific recombinant IgG was used for control and did not accumulate in any brain region at 24 h (Appendix Fig S8G). Hence, regional pharmacokinetic differences do not explain the region‐specific drug effects. We also tested the abundance of Aβ and BACE1 in eight brain regions (Appendix Fig S8A) biochemically (Appendix Fig S9). We could not differentiate between brain regions biochemically; however, both LIN5044 and NB360 reduced the amount of Aβ monomers compared with control mice (Appendix Fig S9).

### Genetic markers revealed by aligning the Q3D output to gene‐expression atlases of the brain

**Figure 5:**
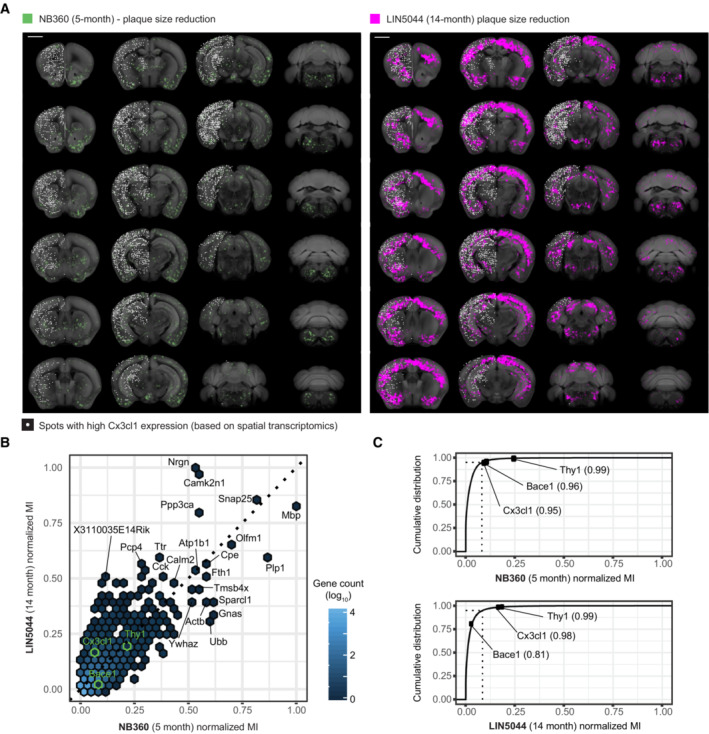
Genetic markers may predict local drug responsiveness SAV plaque‐size‐reduction maps for NB360‐5‐month and LIN5044‐14‐month effects overlaid with data from a spatial transcriptomics database, with the Cx3cl1 gene shown as an example. Spots with high Cx3cl1 expression (white) show more overlap with the LIN5044 effect than with NB360. Calculating the mutual information between each SAV and gene expression pair allows for the discovery of candidate genetic markers that predict each drug's responsiveness. Scale bars: 2 mm.Normalized Mutual Information (MI) of genes above the 95^th^ percentile (ranked by MI, [*n* = 1,169]) for either NB360 or LIN5044.MI between SAV and gene expression ranked cumulatively by MI (23,371 genes). Cx3cl1, a neuron‐borne microglia chemoattractant, is a higher ranked genetic marker for LIN5044 responsiveness. Dashed line: genes above the 95^th^ percentile of MI.

The findings above suggested that the local heterogeneity of drug efficacy may be controlled by intrinsic properties of the host brain. We therefore compared the plaque‐size SAVs of NB360 and LIN5044 to a gene‐expression atlas reporting whole‐genome expression at > 30,000 spots of the mouse brain (Ortiz *et al*, 2020; Fig 5A). We calculated the mutual information (MI), a similarity metric describing the nonlinear interdependence of random variables. We also created an online application for browsing these data (https://fgcz‐shiny.uzh.ch/SPAGEDI/). As expected, most genes showed low MI scores with either treatment (Fig 5B). The MI of *Thy1*, whose promoter was used to drive APP/PS1 expression, ranked at the 99^th^ percentile of 23,371 genes for both LIN5044 and NB360 (MI: 8.06 × 10^−4^; NB360 5.32 × 10^−4^, respectively). Similarly, *Bace1* expression correlated highly with the efficacy of its inhibitor NB360 (MI: 2.22 × 10^−4^, 96^th^ percentile), but not with LIN5044 (1.37 × 10^−4^, 81^st^ percentile; Fig 5C). These results confirm that the morphological‐genetic analyses presented here identify sensitively and reliably the genetic networks controlling the efficacy of amyloid removal therapies.

**Figure EV5:**
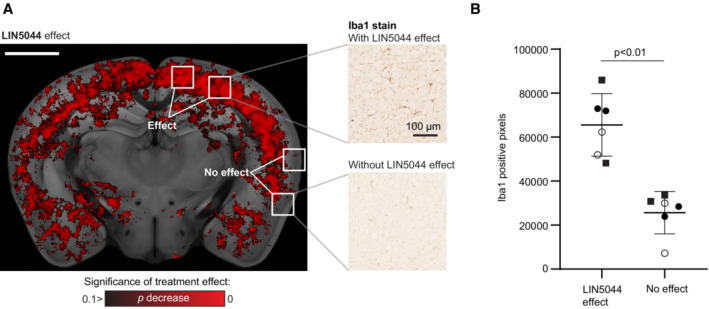
Histological sectioning and Iba1 labeling validates increased microglia density in regions where LIN5044 was more effective in reducing plaque size Regions of interest were selected based on voxel‐based statistics of LIN5044 efficacy in reducing plaque size in 14‐month‐old mice. Microglia density was measured in regions displaying both strong or absent LIN5044 efficacy (3 three wild‐type mice, 3 slices/mouse). Scale bar: 2 mmRegions showing strong LIN5044 effects contained more microglia (each distinct symbol represents one mouse).

The effect of LIN5044 showed a high MI (7.23 × 10^−4^, 98^th^ percentile) with Cx3cl1, a microglial chemoattractant mostly expressed by neurons (Tarozzo *et al*, 2003; NB360, Cx3cl1 MI: 1.95 × 10^−4^, 95^th^ percentile; Fig 5), potentially suggesting that the effects of LIN5044 are stronger in areas of high microglia recruitment. To test this, we quantified Iba1^+^ microglia in cortical areas with strong or weak LIN5044 effects. Regions strongly affected by LIN5044 showed significantly higher microglia counts (Fig EV5).

## Discussion

Brain‐wide analysis of Aβ plaque load, as shown previously (Liebmann *et al*, 2016), requires reliable brain clearing and amyloid staining (Richardson *et al*, 2021). However, current hydrogel‐based methods for whole‐brain clearing are slow and often inhomogeneous. We have solved these limitations by insulating the anodic from the cathodic detergent reservoir, thereby constraining the lipid‐clearing ion flow through the sample. This design enabled whole‐brain clearing within 14 h (Chung *et al*, 2013; Tomer *et al*, 2014; Richardson & Lichtman, 2015), and electrophoresis of qFTAA/hFTAA resulted in homogenous plaque staining within 2 h. Comparisons between the Q3D pipeline, including light‐sheet acquisition (3.26 × 3.26 × 6.5 μm resolution), with conventional staining of histological sections showed that Q3D reliably visualizes Aβ plaques within mouse brains. The precision of Q3D resulted in highly consistent plaque counts among age‐matched APP/PS1 mice (standard deviation: 2.5–3.3%).

The β1 antibody showed only marginal effects in young APP/PS1 mice and no effect in old mice, consistent with previous reports (Pfeifer *et al*, 2002; Balakrishnan *et al*, 2015). The marginal effect was primarily visible in the brainstem, where higher β1 antibody drug concentrations were measured (Appendix Fig S8). It was shown that peripheral β1 injections result in significant Aβ reduction in the brain and Aβ epitope masking (Pfeifer *et al*, 2002, Balakrishnan *et al*, 2015). Furthermore, the β1 antibody was shown to bind Aβ plaques in the brain and to retain Aβ in the blood after peripheral administration (Winkler *et al*, 2010). However, the limited effect of the β1 antibody may be explained by its low bioavailability relative to the high abundance of Aβ. In contrast, both LIN5044 and NB360 reduced amyloid load more effectively. This provided the first evidence that polythiophenes can be efficacious against Aβ. However, we unexpectedly found that the activities of LIN5044 and NB360 were highly divergent in many ways. As our colocalization analysis revealed, the spatial efficacies of LIN5044 and NB360 were almost mutually exclusive, being more effective against either rostro‐dorsal or ventrocaudal amyloid deposits, respectively. Moreover, LIN5044 was more effective in reducing the growth of existing plaques, rather than their total number. LIN5044 was also more effective in aged mice than in younger mice. In contrast, BACE1 inhibition was most effective in reducing plaque numbers in young mice, yet affected their growth to a lesser extent. The reduction in plaque burden showed steep ventrocaudal (NB360) and dorsorostral (LIN5044) gradients whose boundaries did not correspond to defined neuroanatomical areas or vascular territories. Thus, the voxel‐level analysis was crucial in finding clusters of treatment significance beyond conventional anatomical boundaries.

The strong Aβ‐monomer reducing effect of BACE1 decreases plaque loads in young, but not in old mice, suggesting that distinct amyloid species are contributing to plaques at different ages. While primary nucleation is mainly dependent on Aβ concentration (Hellstrand *et al*, 2010; Burgold *et al*, 2014) and is highly sensitive to BACE1 inhibition (Brendel *et al*, 2018; Peters *et al*, 2018), secondary nucleation (Cohen *et al*, 2013) may become more important in aged mice. In prion diseases, polythiophenes (including LIN5044) slow disease progression by reducing the number of seeds for secondary nucleation. This is achieved by polythiophenes binding and stabilizing amyloid fibrils, resulting in reduced fibril fragmentation as a source of seeds (Margalith *et al*, 2012; Herrmann *et al*, 2015). Similarly, Aβ fibril hyper‐stabilization could explain the effect of LIN5044 in APP/PS1 mice. The remarkable efficacy of LIN5044 in aged mice may relate to the dependency of secondary nucleation onto the concentration of seeds, which increases with age. Accordingly, LIN5044 in older mice primarily reduced mean plaque sizes (Fig 2G), all while increasing the number of smaller plaques (Fig 2F), providing further evidence that LIN5044 reduces additive plaque growth. Potentially, smaller plaques might be a consequence of an increase in plaque compaction upon LIN5044. The stronger effect of LIN5044 in old mice suggests that it preferentially targets tightly packed plaques, which become more abundant with time.

Pharmacokinetic analyses did not support the notion that differential bioavailability may account for the regiospecific activities of NB360 and LIN5044 (Appendix Fig S8). Also, we did not detect regional differences in endogenous Aβ and BACE1 levels. Because of its inherent fluorescence, LIN5044 might also influence the spectral properties of plaques. We therefore compared plaque spectra in a mouse injected i.p. with LIN5044 (0.4 mg) and one injected with PBS. No differences were detected, suggesting that the effect of LIN5044 on plaque maturity is not artefactual (Fig EV4A).

By aligning SAV heatmaps to a spatial transcriptomic atlas (Ortiz *et al*, 2020), we identified a strong spatial similarity between *Bace1* expression and the regiospecific efficacy of NB360, but not of LIN5044. *Thy1*, the promoter driving the APP/PS1 mouse model, ranked highly for both treatments, providing validation for the unsupervised approach chosen here. LIN5044 efficacy showed high spatial similarity with transcription maps of CX3CL1, a neuron‐borne microglial chemoattractant (Szepesi *et al*, 2018). The regional variation of CX3CL1 expression may result in differential recruitment of microglia, thereby contributing to the clearance of LIN5044‐intercalated amyloid. Indeed, follow‐up experiments confirmed that the areas where LIN5044 had a stronger effect also harbored more microglia in APP/PS1 mice. The cognate microglial receptor CX3CR1 is highly expressed at late AD stages (Chen *et al*, 2020), which may partly explain the potency of LIN5044 in old mice. As shown here, correlating spatial transcriptomic atlases to therapy atlases can be used as a tool to generate hypotheses for treating Aβ pathology. However, when using such an approach, one needs to take into consideration that gene expression and protein localization might not coincide. In the future, such spatial analyses could benefit from incorporating proteomic data.

Our results suggest that the regional variation and age dependence of anti‐amyloid drug efficacy could considerably influence clinical trial efficacy. However, to what extent regional variation in the effects of CNS drugs translates from mouse to human is unclear. Systematic regional neuropathological analysis after AD clinical trials could shed light on regional differences in drug response. Since LIN5044 and NB360 affect mostly non‐overlapping areas of the brain, combinatorial regimens may synergistically protect larger brain volumes from amyloid deposition. Notably, the computational methods developed for Aβ amyloid quantification can enhance the statistical power of the *in vivo* assessment of anti‐Aβ drugs and can be adapted to a broad range of protein aggregation diseases.

## Materials and Methods

### Animal treatments and tissue preparation

All animal experiments were carried out in strict accordance with the Rules and Regulations for the Protection of Animal Rights (Tierschutzgesetz and Tierschutzverordnung) of the Swiss Bundesamt für Lebensmittelsicherheit und Veterinärwesen at the animal facility of the University Hospital Zurich, Switzerland, and were preemptively approved by the Animal Welfare Committee of the Canton of Zürich (permit 040/2015). APP/PS1 (Radde *et al*, 2006; KOESLER, Rottenburg, Germany) male and female mice were either treated with NB‐360 (Neumann *et al*, 2015) BACE1‐inhibitor (Novartis) orally (0.5 g inhibitor/kg chow, ~ 6 g chow/day/mouse = 3 mg inhibitor/day/mouse), with the β1 monoclonal IgG2a antibody recognizing the human‐specific EFRH tetrapeptide of amino acids 3–6 of Aβ (Paganetti *et al*, 1996; Pfeifer *et al*, 2002; Balakrishnan *et al*, 2015; Novartis; 0.5 mg/week, once/week in 200 μl phosphate‐buffered saline (PBS) intraperitoneally), or with the amyloid intercalator LIN5044 (0.4 mg/week, once/week in 100 μl PBS) based on previous reports. Because the pharmacokinetics of LIN5044 was unknown, the dose was selected based on previous work (Herrmann *et al*, 2015). Control mice were treated with control chow, pooled recombinant nonspecific IgG, and PBS, respectively. The ages of NB‐360, β1 antibody, and LIN5044‐treated mice were 353 ± 21, 313 ± 9, and 308 ± 7 days, respectively (groups of old mice), as well as 61 ± 3 days, 59 ± 2, and 65 ± 2 days, respectively (groups of young mice; Table 1). After treatments were completed, mice were deeply anesthetized with ketamine and xylazine, then transcardially perfused with ice‐cold phosphate‐buffered saline (PBS) followed by a hydrogel monomer mixture of 4% acrylamide, 0.05% bisacrylamide, and 1% paraformaldehyde (Yang *et al*, 2014). Brains were harvested and further incubated passively in the hydrogel mixture for 24 h. The hydrogel was degassed and purged with nitrogen, followed by polymerization at 37°C for 2.5 h. Samples were either stored in PBS or clearing solution until Q3D clearing.

For generating the whole‐brain vascular images (Appendix Fig S1H), Claudin5‐GFP (Gensat.org. Tg(*Cldn5*‐GFP)) mice were processed as described above.

### Tissue clearing

Brains were cleared with focused electrophoretic tissue clearing (FEC) in a custom‐built chamber in 8% clearing solution (8% w/w sodium dodecyl sulphate in 200 mM boric acid, pH 8.5). Standard settings were 130 mA current‐clamped at a voltage limit of 60 V, at 39.5°C. Clearing time varied between 6 and 14 h for a whole mouse brain. Tissue clarity was determined by visual inspection. The polarity of the electrodes was switched after approximately 50% of the total clearing time. Transparency was assessed by visual inspection. The clearing solution was circulated from a buffer reservoir of 250 ml. After clearing a brain, 60 ml of clearing buffer was exchanged with fresh buffer before starting to clear the next sample. Q3D‐clearing chambers were 3D‐printed, and clearing was done in an incubator at 39.5°C. For details on the chamber design, see supplementary material and model repository (Appendix Fig S1).

### Relative electrical resistivity measurement between brain tissue and buffer

For electrical resistivity measurements comparisons, mouse brains were first fixed in 4% paraformaldehyde, then measured in PBS. Platinum electrodes (1.5 × 0.2 mm) were mounted at a distance of 2 mm, and a constant voltage of 30 V was applied. Current measurements were used to calculate the resistance between the electrodes. Brain tissue resistance was measured by sticking the electrode pair into the brain at three different locations (frontal cortex, occipital cortex, and brainstem). At each location, current values were measured three times. The resistivity in the buffer was measured nine times. These measurements resulted in a ~ 1,200‐Ohm resistance in the brain and ~ 300‐Ohm resistance in the buffer. As the electrode setup and the voltages were constant for every measurement, the relative resistivity was calculated as the ratio of the two resistivity measurements, resulting in a resistivity ratio of 1:4 (buffer:brain).

### Comparison between Q3D and CLARITY

Hydrogel‐embedded brains from 3‐month‐old mice were either passively cleared with 8% clearing solution for 24 h at 39°C (*n* = 3) or for 4–6 h with either Q3D or CLARITY (Chung *et al*, 2013) clamped at 130 mA at 60 V at 39°C (four active clearing groups, each *n* = 3). After clearing, brains were washed with PBS, followed by refractive index‐matching and mounting in quartz cuvettes. Samples were illuminated with a ~ 4–5 μm wide 647‐nm laser beam, calibrated to 0.35‐mW power. Each brain was illuminated stereotypically from the dorsal end at 10 aligned points with 0.5 mm spacing along the rostro‐caudal axis, with the same incumbent laser power (as measured through the imaging medium and cuvette). This was done in three parallel lines (one in the midline, and a line 2 mm left and right from it), resulting in 3 × 10 measurement points per brain (Appendix Fig S1F). The point pattern was defined by starting from the lambdoid fossa. The transmitted light was measured at each point with a digital optical power meter (Thorlabs, PM100D, Compact Power and Energy Meter Console, Digital 4″ LCD; Thorlabs S130C photodiode sensor). The mean and standard error of the mean for all points for each sample were plotted (Appendix Fig S1F and G). We also plotted the mean transmitted light on every rostro‐caudal level, resulting in 10 datapoints summarizing the three parallel lines (two hemispheres and the midline; 0.35 mW input power, 647 nm wavelength, 3 brains/group, 30 measurement points/brain, Fig 1B and Appendix Fig S1). These 10 points per sample were submitted to a one‐way ANOVA (α = 0.05), which was corrected with Tukey's test for multiple comparisons.

### Electrophoretic staining with Q3D

Histochemistry by iontophoretic tissue staining was initially done by layering paraffin and agarose around the sample in order to limit current flow to the tissue. Later, Q3D staining chambers were 3D‐printed (Appendix Fig S2A and B). In total, 50 mM tris and 50 mM tricine at a pH of 8.5 were used as the electrophoresis buffer. Tests in native polyacrylamide gel electrophoresis were followed by silver staining (Thermo Scientific Pierce Silver Stain Kit, #24612) that showed, as expected, that electrophoretic mobility is highly dependent on the pH and buffer (Appendix Fig S2D–G). Amyloid plaques were stained with a combination of luminescent conjugated polythiophenes (LCP), heptamer‐formyl thiophene acetic acid (hFTAA), and quadro‐formyl thiophene acetic acid (qFTAA). The combination of these dyes was used for the discrimination of neuritic plaques (Nystrom *et al*, 2013) at different maturation states (Rasmussen *et al*, 2017).

### Refractive index matching

Brains that were cleared and stained with Q3D were refractive index (RI) ‐matched to 1.46 with a modified version of the refractive index matching solution (Yang *et al*, 2014) by including triethanolamine (tRIMS). tRIMS was made by mixing Histodenz (Sigma #D2158; 100 mg), phosphate‐buffered saline (75 ml), sodium azide (10% w/v, 500 μl), tween‐20 (75 μl), and triethanolamine (42 ml). tRIMS maintained the RI while reducing the amount of Histodenz required and improving transparency. After prolonged air and light exposure, tRIMS tends to undergo browning; however, in air‐tight tubes at 4°C, samples remain stable for at least 2 years.

### Antibody and polythiophene staining

Slices from formalin fixed and paraffin‐embedded brain tissue from a 13‐month‐old APP/PS1 mouse were stained for Aβ plaques. Slices were stained with mouse anti‐human Aβ_17‐24_ antibody (4G8, AB2734547 Biolegend Cat. No. 800708) after antigen retrieval with 10% formic acid. Slices were blocked with M.O.M. Kit (BMK‐2202), and the primary antibody was detected with Alexa‐594 conjugated goat anti‐mouse IgG (Invitrogen A‐11005, 1:1,000 dilution). Alternatively, slices were stained with Aβ N3pE rabbit anti‐human antibody (IBL 10045, Clone 82E1, 1:50 dilution) after 10% formic acid antigen retrieval, followed by blocking with 10% goat serum, detected with Alexa‐594 conjugated goat anti‐rabbit IgG (Invitrogen A‐11037, 1:500 dilution). Both antibody stainings were followed by staining with qFTAA (0.75 μM in PBS) and hFTAA (3 μM in PBS) for 30 min, followed by diamidino‐phenylindole (DAPI) staining. Slices were imaged with a Leica SP5 confocal microscope with a 10× air objective (numerical aperture 0.4). The qFTAA and hFTAA stainings were imaged by exciting both at 488 nm and collecting emission between 493–510 nm and 530–579 nm, respectively. The dynamic range of images was adjusted consistently across stainings, and images (3.57 μm/pixel) were median filtered with ImageJ (pixel radius 0.5).

To test the effect of the LIN5044 treatment on the qFTAA+hFTAA plaque maturity analysis, 12‐month‐old APP/PS1 mice (*n* = 2) were injected i.p. with LIN5044 (0.4 mg in 100 μl PBS; *n* = 1) and PBS (100 μl; *n* = 1). After 3 days mice were deeply anesthetized with ketamine and xylazine and transcardially perfused first with ice cold PBS, followed by 4% paraformaldehyde. Brains were harvested and were further incubated in the paraformaldehyde solution for 24 h. Brains were then incubated in 30% sucrose in PBS for 2 days at 4°C. Next, brains were snap‐frozen and stored at −80°C overnight. Brains were embedded in tissue freezing medium (Leica biosystems), and 20 μm coronal slices were cut with a cryostat and mounted on Super‐frost micro‐slides. Tissue slices were fixed in 10% formalin overnight and rehydrated by dipping them in consecutive baths of 99% ethanol, 70% ethanol, dH_2_O, and PBS, 10 min in each. The tissue sections were allowed to dry under ambient conditions. For polythiophene staining, 200 μl of hFTAA and qFTAA solution (20 μM) were added onto the tissue sections to cover them and slices were incubated for 30 min at room temperature. Sections were rinsed in a PBS bath for 10 min, followed by nuclear staining with DAPI. Tissue sections were dried under ambient conditions, followed by mounting with fluorescence mounting medium (DAKO). Slides were imaged with a Leica SP5 Confocal microscope. Nuclei and plaques were imaged with a 10×/0.25 NA dry objective, using the following settings: 405/30 nm excitation/emission filters for DAPI (nuclei) and 498–520 nm (compact amyloid) and 565‐605 nm (looser amyloid) for LCPs. Laser power was set on 10% for all the conditions and a line average of 96 was used for acquisition. Three 8‐bit images were recorded from each sample. Ten plaques per image were measured for their fluorescent intensity by quantifying the maximum intensity of a line drawn through each individual plaque with Fiji. The mean intensity of 10 plaques per image was compared with a two‐tailed *T*‐test between the two conditions (LIN5044 or PBS treated).

For microglia immunohistochemistry, fixed brains of LIN5044‐treated transgene‐negative littermates from APP/PS1 nests (*n* = 3) were embedded in paraffin. 4‐μm‐thick paraffin sections (three sections per mouse) were deparaffinized through a decreasing alcohol series. Slices were stained with Iba‐1 antibody (1:1,000; Wako Chemicals GmbH, Germany) and detected using an IVIEW DAB Detection Kit (Ventana). Sections were imaged using a Zeiss Axiophot light microscope. For the quantification of the Iba‐1 staining, in every slice, two regions of interest were selected in the cortex. The four regions of interest were selected representing 2–2 cortical areas with either high or no LIN5044 therapeutic effect (Figs 3A and EV5A). Pixels in the regions of interest were classified and counted as microglia (Iba‐1 positive) or background (Iba‐1 negative) with a manually trained (trained on three images) pixel classifier in ILASTIK (https://www.ilastik.org/), and ImageJ. Hypothesis testing was done with a two‐tailed *T*‐test.

### Drug distribution measurements

NB360 was administered orally in three male C57BL/6 black mice at 5 mg/kg body‐weight dose level at 0.5 mg/ml in water with 0.5% methylcellulose and 0.1% Tween‐80. Based on preceding pharmacokinetic studies (Neumann *et al*, 2015), brains were harvested 1 h later, followed by homogenization in water and acetonitrile precipitation. NB360 levels were measured with tandem mass spectrometry with electrospray ionization. LIN5044 was administered intraperitoneally into male C57BL/6 black mice at 16 mg/kg body‐weight dose level at 4 mg/ml in PBS. As preceding pharmacokinetic studies were not available, 2‐ and 6‐h incubation timepoints were chosen, each with three mice. Brains were homogenized in water and precipitated with methanol. LIN5044 levels were measured with high pressure liquid chromatography–tandem fluorescence detection. Brain regions' drug levels were compared with one‐way ANOVA (α = 0.05) for each compound and timepoint separately (NB360, LIN5044 2 h, LIN5044 6 h); multiple comparisons were corrected for with Tukey's test.

### Antibody brain‐distribution measurements

C57BL/6 mice were injected one‐time with either β1 antibody (*n* = 6), as a control with pooled recombinant nonspecific IgG  (*n* = 2; 0.5 mg in 200 μl intraperitoneally), or with no injection (*n* = 1). β1‐injected mice were sacrificed after 6 (*n* = 3) or 24 h (*n* = 3), while control mice were sacrificed 24 h after injection. Amyloid β Protein Fragment 1–42 (A9810, Sigma) was diluted at 1 μg/ml in PBS and passively absorbed on multiwell plates (SpectraPlate‐384 HB, Perkin Elmer) overnight at 4°C. Plates were washed three times in 0.1% PBS‐Tween 20 (PBS‐T) and blocked with 80 μl per well of 5% skim milk (Migros) in 0.1% PBS‐T, for 2 h at room temperature. β1‐antibody and pooled recombinant IgG were used as positive and negative controls, respectively. Blocking buffer was discarded, and both samples and controls were dissolved in 1% skim milk in 0.1% PBS‐T for 1 h at 37°C. Twofold dilutions of β1‐antibody, starting at a dilution of 1,000 ng/ml in 1% skim milk and in 0.1% PBS‐T, were used for a calibration curve. Goat polyclonal anti‐mouse antibody (1:1,000, 115‐035‐062, Jackson ImmunoResearch) was used to detect murine antibodies. Chromogenic reaction was induced by addition of TMB Stabilized Chromogen (SB02, Thermo Fisher Scientific) and stopped by addition of 0.5 M H_2_SO_4_. Absorbance was read at λ = 450 nm. Unknown β1‐antibody concentrations were interpolated from the linear range of the calibration curve using linear regression (GraphPad Prism, GraphPad Software).

### Western blots of Aβ and BACE1

Brain hemispheres from mice treated with LIN5044 (0.4 mg/week, once/week in 100 μl PBS; *n* = 1) or PBS (*n* = 1) intraperitoneally, or with NB360 chow (0.5 g inhibitor/kg chow, ~ 6 g chow/day/mouse = 3 mg inhibitor/day/mouse; *n* = 1) or control chow (*n* = 1) for 3 months, were dissected into eight anatomical regions: rostro dorsal, rostro ventral, medio dorsal, medio ventral, caudo dorsal, caudo ventral, brain stem, cerebellum (Appendix Fig S8A). Each region was homogenized using Ribolyser for 5 min in 500 μl lysis buffer (140 mM NaCl, 20 mM TrisHCl, pH 7.5, protease inhibitors [complete Mini, Roche], phosphatase inhibitors [PhosphoSTOP, Roche] in PBS), and centrifuged at 15,000 *g* for 30 min at 4°C. Subsequently, the supernatant of each sample was isolated, and the pellet was resuspended in 200 μl of lysis buffer with the addition of 0.5% SDS to obtain the insoluble fraction. In total, 2  μl of Dithiothreitol (DTT) was added to 20 μl of each fraction. Samples were loaded on a SDS‐PAGE (Novex NuPAGE 4–12% Bis‐Tris Gels). After electrophoresis, gel was transferred to iBlot I (Invitrogen) and transferred onto polyvinylidene difluoride (PVDF) membrane. Membranes were blocked in 5% Sureblock for 1 h at room temperature followed by incubation at 4°C overnight with 1:1,000 dilution of the following primary antibodies: mouse monoclonal to human amyloid beta 1–16, clone 6E10 (Sigma) or rabbit polyclonal to BACE1 (abcam ab2077). Membranes were washed 3× (10 min each) with PBS‐Tween (0.1%) followed by incubation with HRP‐tagged secondary antibody (Peroxidase‐Goat Anti‐Mouse IgG (H+L; #62‐6520) or Peroxidase‐Goat Anti‐Rabbit IgG (H+L; #111.035.045); 1 h at room temperature) and further washes (3×, 10 min). Membranes were developed with Luminata Crescendo (Millipore), and images were acquired using Fusion Solo S (Vilber).

### Whole‐brain imaging

Whole brain images were recorded with a custom‐made selective plane illumination microscope (mesoSPIM; Voigt *et al*, 2019). SPIM imaging was done after clearing and refractive index matching. The laser/filter combinations for mesoSPIM imaging were as follows: for qFTAA at 488 nm excitation, a 498–520 nm bandpass filter (BrightLine 509/22 HC, Semrock/AHF) was used as the emission filter; for hFTAA at 488 nm excitation, a 565–605 nm bandpass filter (585/40 BrightLine HC, Semrock/AHF) was used. Transparent whole brains were imaged at a voxel size of 3.26 × 3.26 × 3 μm^3^ (X × Y × Z). For scanning a whole brain, 16 tiles per channel were imaged (eight tiles per brain hemisphere). After the acquisition of one hemisphere, the sample was rotated, and the other hemisphere was then acquired. The entire process resulted in typical acquisition times of 2–3 h, followed by stitching (Bria & Iannello, 2012). Data accumulated from one brain ranged around 600 GB in size. Further technical details of the mesoSPIM have been previously reported (Voigt *et al*, 2019). The scientist imaging the brains was not blinded to the experimental ID of the mice.

### Computational and statistical analysis

The following computations were performed using custom scripts written in Python and R as well as existing third‐party libraries (Table 2). The two‐channel (498–520 nm and 565–605 nm) substacks for each brain hemisphere were first stitched together with Terastitcher (Bria & Iannello, 2012). The result was downsampled from the acquired resolution (3.26 μm lateral, 3 μm depth) to an isotropic 25 μm resolution and then registered to the Allen Institute 25 μm average anatomical template atlas (Wang *et al*, 2020). This was performed automatically using a combination of affine and b‐spline transformation registrations with a mutual information similarity metric, using parameters influenced from a previous study performing mouse whole‐brain fluorescence quantification (Renier *et al*, 2016). The resulting pairs of transformations were used in subsequent steps to transform coordinates in the raw data space to the template atlas space (Appendix Fig S4).

The 565–605 nm channel at its original resolution was used to determine the locations of aggregates of amyloid‐β stained with qFTAA and hFTAA. A random forest classifier was used to classify each voxel as either “belonging to a plaque” or “background.” This classifier was generated using the open‐source Ilastik framework (Berg *et al*, 2019) and trained on a random subset of data (random stacks, three stacks [187 × 176 × 1242 pixels] picked from every experimental group) that was separately annotated by two neuropathologists. Amyloid‐β aggregates were considered to be the individually connected components from this binarized volume. The three‐dimensional center of mass and total volume were then calculated for each component. Connected components with a volume below a global threshold were considered noise and ignored. The centers of mass were used to look up the peak fluorescent intensity of each plaque in the 498–520 nm (qFTAA) channel. Plaque maturity was calculated for each plaque as its peak intensity in the 498–520 nm (qFTAA) channel divided by its peak intensity in the 565–605 nm (hFTAA) channel (Nystrom *et al*, 2013; Fig 2C).

After downsampling each aggregate center to 25‐μm resolution and applying the optimized registration transformation, the number of aggregates were counted at each voxel in this atlas space (Appendix Fig S4E and F). Smoothed heatmaps were generated by placing a spherical ROI with 15‐voxel diameter (= 375 μm) at each voxel and summing the plaque counts within the ROI. This ROI diameter was set to match the mean spatial Jacobian‐matrix determinant of the previously registered b‐spline transformation across all samples. This method for smoothing and accounting for variable registration quality has also been described in a previous whole‐brain study (Renier *et al*, 2016). Voxel‐level statistics across treated and control brains involved running a two‐sided *t*‐test at each heatmap voxel across the two groups. Each voxel *P*‐value was adjusted using the Benjamini–Hochberg method (Benjamini *et al*, 2001). These adjusted *P*‐value maps were then binarized with a threshold of 0.05 or 0.10 for subsequent analysis or visualization.

The transformed locations of each plaque were also further grouped into 134 different anatomically segmented regions in the Allen Reference Atlas (Wang *et al*, 2020) for further statistical analysis between longitudinal groups (Appendix Figs S4G and S10). Similar heatmap generation, voxel statistics, and regional statistics were performed for two other metrics: the mean plaque volume and the mean plaque maturity. For all statistical tests, no sample size estimation methods were used.

The voxel‐level statistical map for each treatment group was also compared against a spatially resolved transcriptomics database consisting of 23,371 genes across 34,103 locations throughout the brain (Ortiz *et al*, 2020). Since the data from this study and the spatial transcriptomic database were both in the ARA coordinate space, it was possible to spatially compare each gene's expression map with each treatment group's binarized *P*‐value map using a similarity metric. In this case, mutual information was used as the similarity metric since it will detect any sort of linear or nonlinear relationship between these two discrete datasets. In order to rank the relevancy of genes in contributing to the spatial map of plaque removal efficacy for a particular compound, we generated a ranked list of genes for each treatment group, sorted by their corresponding mutual information score. We also created an online browser for the MI database (https://fgcz‐shiny.uzh.ch/SPAGEDI/).

Key Resources are summarized in supplemental table 2 (Table 5). See also References (Bradski, 2000; Klein *et al*, 2010; Millman & Aivazis, 2011; Furth *et al*, 2018 *)*.

**Table 5:** Third‐party libraries used for the computational pipeline.

Software and algorithms	Source
ClearMap	Renier, N *et al* (2016) Mapping of Brain Activity by Automated Volume Analysis of Immediate Early Genes. *Cell* **165**: 1789–1802 10.1016/j.cell.2016.05.007
Python	Python Software Foundation. Python Language Reference, version 2.7 (http://www.python.org)
FIJI	Schindelin J *et al* (2012) Fiji: an open‐source platform for biological‐image analysis. Nat Methods **9**: 676–682
R	R Core Team (2013) R: a language and environment for statistical computing. R Foundation for Statistical Computing, Vienna, Austria (http://www.R‐project.org)

## Author contributions

**Daniel Kirschenbaum:** Conceptualization; data curation; validation; investigation; visualization; methodology; writing—original draft; writing—review and editing. **Ehsan Dadgar‐Kiani:** Data curation; software; formal analysis; validation; visualization; methodology; writing—original draft; writing—review and editing. **Francesca Catto:** Data curation; validation; investigation. **Fabian F Voigt:** Methodology. **Chiara Trevisan:** Investigation. **Oliver Bichsel:** Investigation. **Hamid Shirani:** Resources. **Peter R Nilsson:** Resources. **Karl J Frontzek:** Investigation. **Paolo Paganetti:** Resources. **Fritjof Helmchen:** Methodology. **Jin Hyung Lee:** Conceptualization; supervision; funding acquisition; writing—original draft; project administration; writing—review and editing. **Adriano Aguzzi:** Conceptualization; supervision; funding acquisition; writing—original draft; project administration; writing—review and editing.

## Disclosure and competing interests statement

J.H.L. is a founder, consultant, and shareholder of LVIS. The University of Zurich has filed a patent protecting certain aspects of the rapid‐clarification technology described here. Prof. Adriano Aguzzi is a member of the EMM Editorial Board. This has no bearing on the editorial consideration of this article for publication.

## For more information

Correlations between spatial transcriptomic data and drug efficacy: https://fgcz‐shiny.uzh.ch/SPAGEDI/.

**The paper explained**

**Problem**

Alzheimer's disease (AD) is an incurable neurodegenerative brain disease. The amyloid beta protein, mainly produced by nerve cells, is considered to play a major role in the development of AD. Many compounds were shown to reduce amyloid beta in the brain of mice leading to improved cognition. While amyloid beta reduction in humans was successful in some clinical trials, the improvement in brain function has been less convincing. There are several reasons why this might be the case. For example, amyloid beta might play a smaller role in AD than previously thought, or AD is a result of a combination of factors. Alternatively, reducing amyloid beta is important, but the treatment must happen in the right time and in the relevant areas of the brain. Therefore, we need to understand *when & where* amyloid beta drugs target the brain.

**Results**

To test *when & where* AD drugs target amyloid beta in the brain, we developed a novel technology, termed Q3D, to microscopically measure changes in amyloid beta across the entire brain of mice. The development of Q3D included the development of protocols that turn mouse brains transparent while staining amyloid beta aggregates. Next, we scanned the brain samples with a selective plane illumination microscope (SPIM). The SPIM scans provide entire mouse brain scans at a microscopic resolution. At this resolution, we can count all the amyloid beta aggregates, called plaques, in the brain. We quantified amyloid beta plaques in the entire brain and used it as a proxy to measure *when & where* AD drugs change amyloid beta. Our results show that two compounds we tested, an amyloid beta production inhibitor (NB360) and an amyloid stabilizer polythiophene (LIN5044), affect plaques at different times in different brain regions.

**Impact**

Our experiments revealed that *when & where* NB360 and LIN5044 target amyloid beta is very distinct. This shows that when testing AD drugs in clinical trials, scientists must take into consideration the potential brain‐region and disease‐stage‐specific effects of drugs. These effects may have a major influence on the outcome of clinical trials. AD drugs should be tested *when & where* they impact the brain, both in preclinical and clinical trials.

## Supporting information



Appendix

Expanded View Figures PDF

Source Data for Expanded View and Appendix

PDF+

Source Data for Figure 1

## Data Availability

All data needed to evaluate the conclusions in the paper are present in the paper and/or the Supplementary Materials. The extensive imaging data are available upon request.
3D designs used for 3D printing tissue clearing and staining chambers (https://grabcad.com/library/electrophoretic‐tissue‐clearing‐and‐staining‐chamber‐1) The computational code for browsing the correlations between spatial transcriptomic data and regional drug efficacy (https://github.com/dadgarki/alz‐drug‐3d‐browser) Spatial transcriptomic data (Ortiz *et al*, 2020, DOI: 10.1126/sciadv.abb3446) Lightsheet scans are archived in the Institute of Neuropathology, University Hospital Zurich, and are available upon request.
