# Supplementary material for: Whole‐brain microscopy reveals distinct temporal and spatial efficacy of anti‐Aβ therapies
Source: EMBO Mol Med. 2022 Nov 16;15(1):e16789. doi: 10.15252/emmm.202216789 (PMC9832821; doi:10.15252/emmm.202216789)
Supplement: Supplementary file 1 — Appendix [file EMMM-15-e16789-s004.pdf]

Appendix

Table of contents

Appendix Figure S1

Appendix Figure S2

Appendix Figure S3

Appendix Figure S4

Appendix Figure S5

Appendix Figure S6

Appendix Figure S7

Appendix Figure S8

Appendix Figure S9

Appendix Figure S10

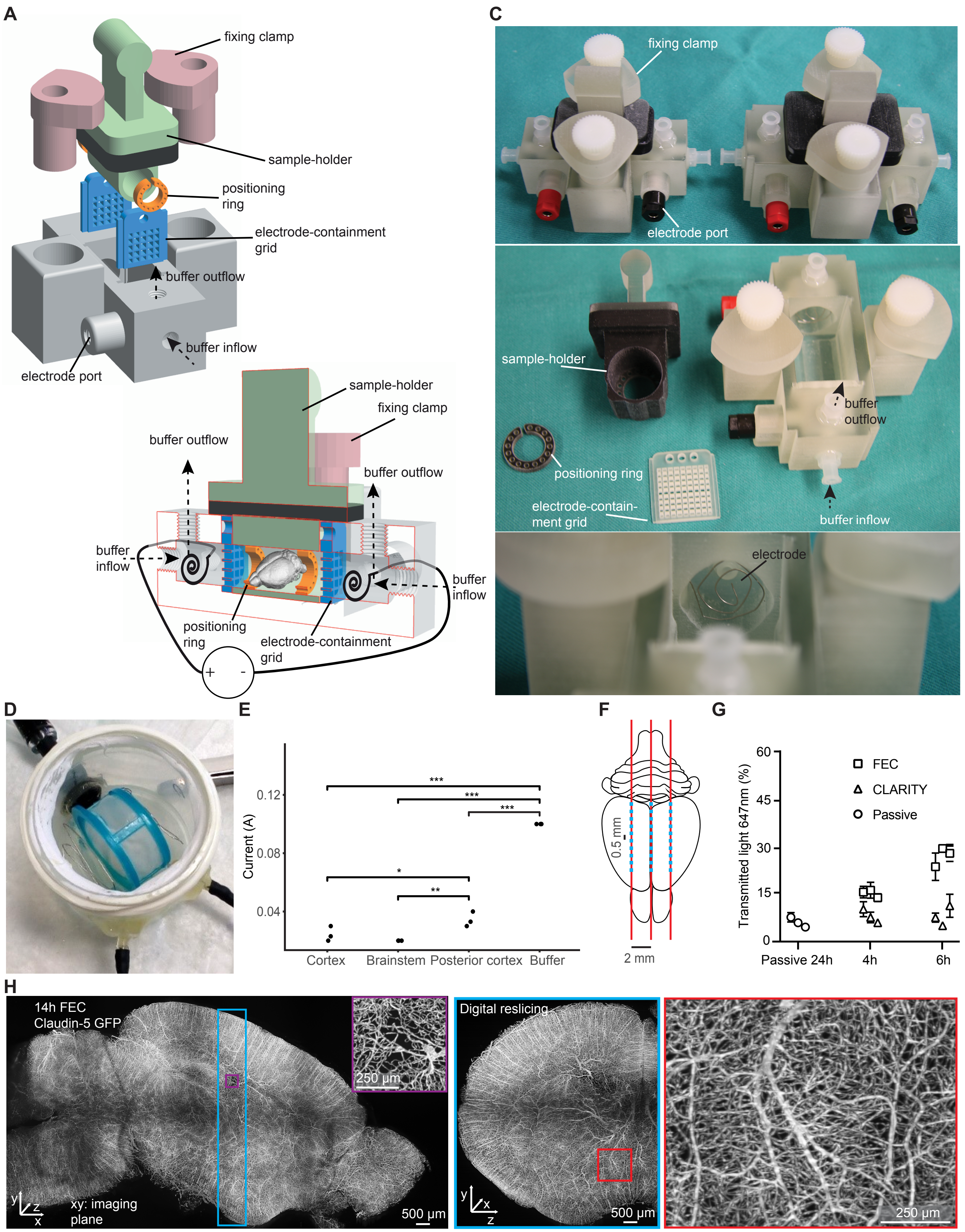

Appendix Figure S1

Appendix Figure S1. Focused electrophoretic tissue clearing (FEC) chambers allow for improved tissue clearing efficiency.

A-C Computer designed and 3D-printed Q3D-clearing chambers. (A) Rendering and cross-section of a chamber. The clearing solution is circulated using two independent, electrically isolated circuits around each electrode. The tissue is contained in a sample-holding plunger (green), and is separated from the electrodes by small nets (blue). The plunger is sealed by a rubber ring (black). Samples are positioned within the tube with rings (orange). (C) 3D printed FEC clearing chambers in different sizes.

D Reproduction of a CLARITY chamber for comparison.

E Electric current measurements in different parts of one 4% paraformaldehyde fixed brain in PBS and in PBS buffer (*$p<0.05$, **$p<0.01$, ***$p<0.001$).

F, G CLARITY and FEC were compared by measuring the light transmitted through the brain in 30 points (f, blue dots). Measurement points were defined along 3 parallel lines 2 mm apart, one in the midline (red lines). Then, for every measurement the laser beam was moved in 0.5 mm steps rostrally starting from the lambdoid fossa. (**G**) Higher transparency in FEC-cleared samples after 4 and 6 hours. Each datapoint represents the mean of all 30 measurements in one mouse brain (n=3 per group).

H The brain vasculature of a Claudin5-GFP mice after 14 hours of clearing was imaged sagittally by mesoSPIM. Digital reslicing and visualization by maximum-intensity projection demonstrates that focused clearing resulted in highly uniform signals. Darkened areas are due to stitching/vignetting artifacts.

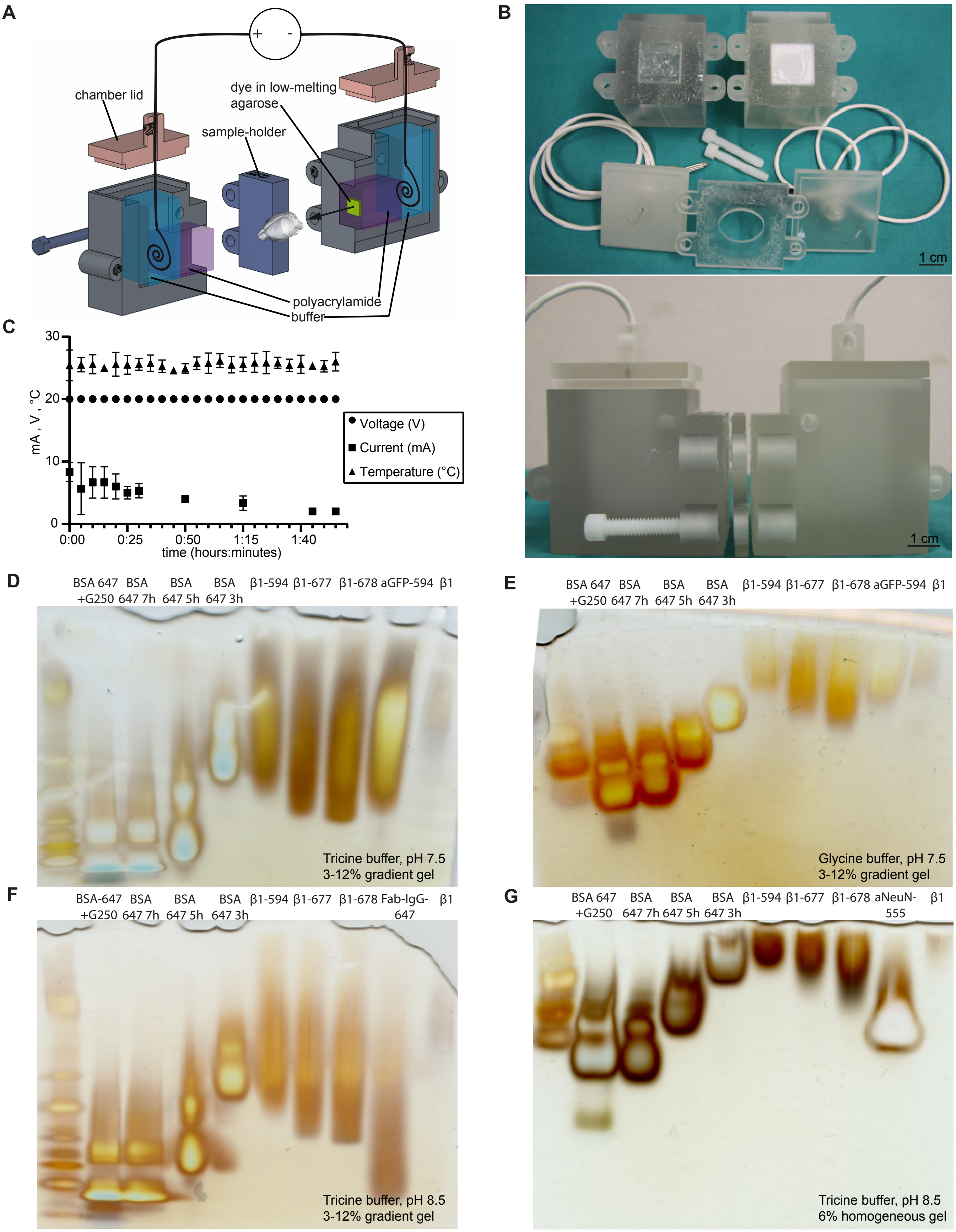

Appendix Figure S2

Appendix Figure S2. Digitally designed and 3D printed setups for convenient electrophoretic staining of tissues.

A, **B** Rendering and 3D prints of electrophoretic tissue staining Q3D chambers. Electrophoresis buffer (light blue) chambers (gray) are separated by two blocks containing polyacrylamide plugs (purple). The sample is placed in a spacer (dark blue) between the plugs. One of the plugs contains the dye solution immobilized in low-melting agarose (green). Each chamber contains a platinum electrode (black).

C Electrophoretic staining was performed at constant voltage and tissue temperature. The electrical current decreased after some minutes.

D-**G** Non-denaturing polyacrylamide gel electrophoresis of various proteins at a range of pH and buffer compositions. (D-F**)** Tricine buffer provided better electrophoretic mobility than (E) glycine buffer, as did higher pH (D: pH 7.5, F: pH 8.5). (D-G) Increasing the electric charge of the β1-antibody by covalently coupling three valent anionic fluorophores (β1-594, β1-677, β1-768) increased electrophoretic mobility. BSA-647 was loaded in 2-hour intervals. In a 6% polyacrylamide native gel (G, while D - F are 3-12 % gradient gels) this resulted in running distances proportional to the run-time. (BSA-bovine serum albumin, β1 - β1 antibody, aNeuN-555-Alexa555 conjugated anti-NeuN antibody, aGFP-594-Alexa594 conjugated anti-GFP antibody). One experimental replicate for each condition.

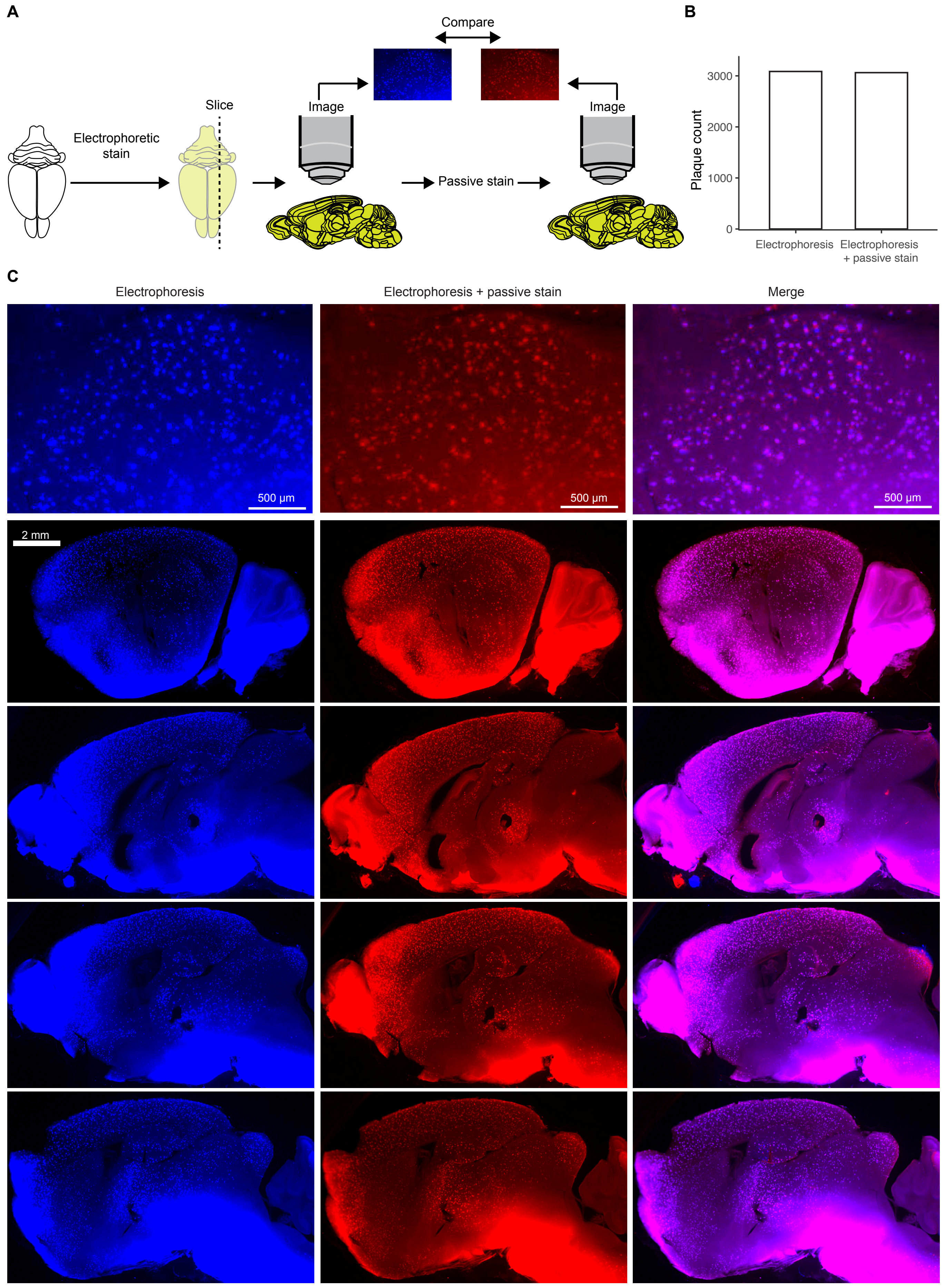

Appendix Figure S3

Appendix Figure S3. Identical Aβ plaque populations are labeled after electrophoretic and passive polythiophene staining.

A A clarified APP/PS1 mouse brain was electrophoretically stained with qFTAA and hFTAA. Sagittal 500-μm sections were imaged with a fluorescent stereomicroscope. After the first round of imaging the slices were passively stained with qFTAA and hFTAA, followed by re-imaging. The images from the two imaging rounds were compared. One biological, four technical replicates.

B No significant difference between the number of plaques in electrophoretically stained and passively re-stained slices.

C Visual inspection of the electrophoretically stained and passively re-stained slices shows close to identical plaque populations. Slight differences between the images are most likely due to the physical manipulations during re-staining and re-imaging.

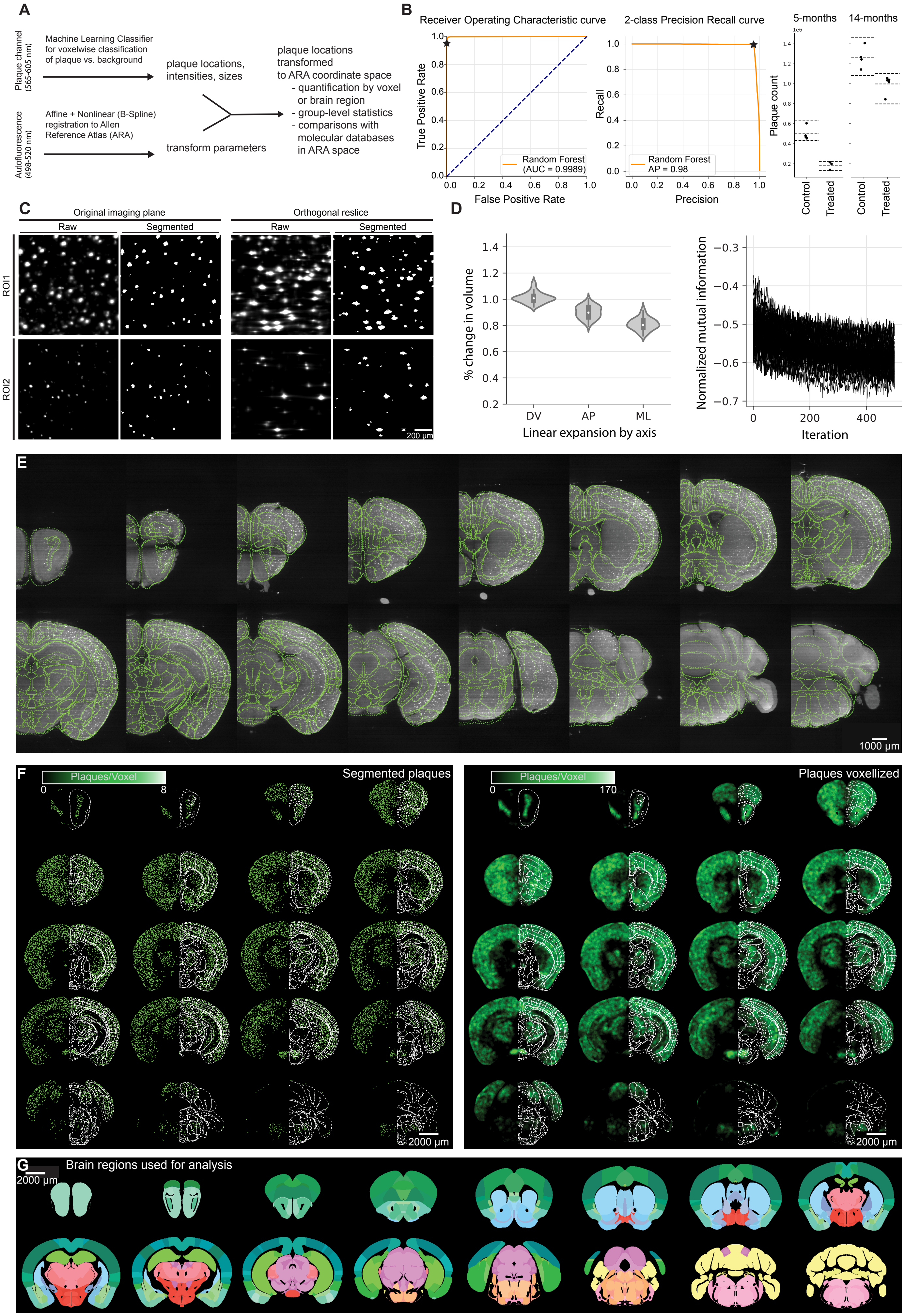

Appendix Figure S4

Appendix Figure S4. Digital atlas registration and machine learning-based plaque segmentation results in precise plaque-load heatmaps.

A Schematic of sample quantification pipeline. Steps in the data analysis pipeline include accurate registration of brains to an atlas using non-linear transformations, plaque segmentation with expert-trained machine learning., and transformation of plaque information to an atlas space where group-level and other analyses can be performed.

B The Receiver Operating Characteristic and Precision Recall curves quantify the classifier's accuracy of plaque detection on an expert-annotated test dataset. When accounting for the classifier's worst-case recall rate (dotted lines, 0.95%), the effect on whole brain plaque count following the NB360 treatment is greater than the variability from overcounting or undercounting whole brain plaques.

C Machine learning-based segmentation fits with raw data. Spherical plaques (Orthogonal imaging plane) show spindle artefacts when digitally resliced orthogonally (Orthogonal reslice – Raw). Segmentation decreased artefacts (Orthogonal reslice – Raw and Segmented).

D Singular values from the registration process capture linear expansion of cleared brains across three spatial axes with respect to the brain atlas. There was consistent shrinking across the anterior-posterior (AP) and medial-lateral (ML) axes, while the dorsal-ventral (ML) axis showed minimal linear expansion. Despite linear expansion, registration optimization metrics all converged to suitable values and

E atlas region contours (green outlines) accurately align with the autofluorescence channels in three dimensions.

F Segmented plaques (left) are voxelized resulting in heatmaps of either plaque density, mean size or maturity (right).

G Statistics on segmented plaques can be done by grouping them into brain regions.

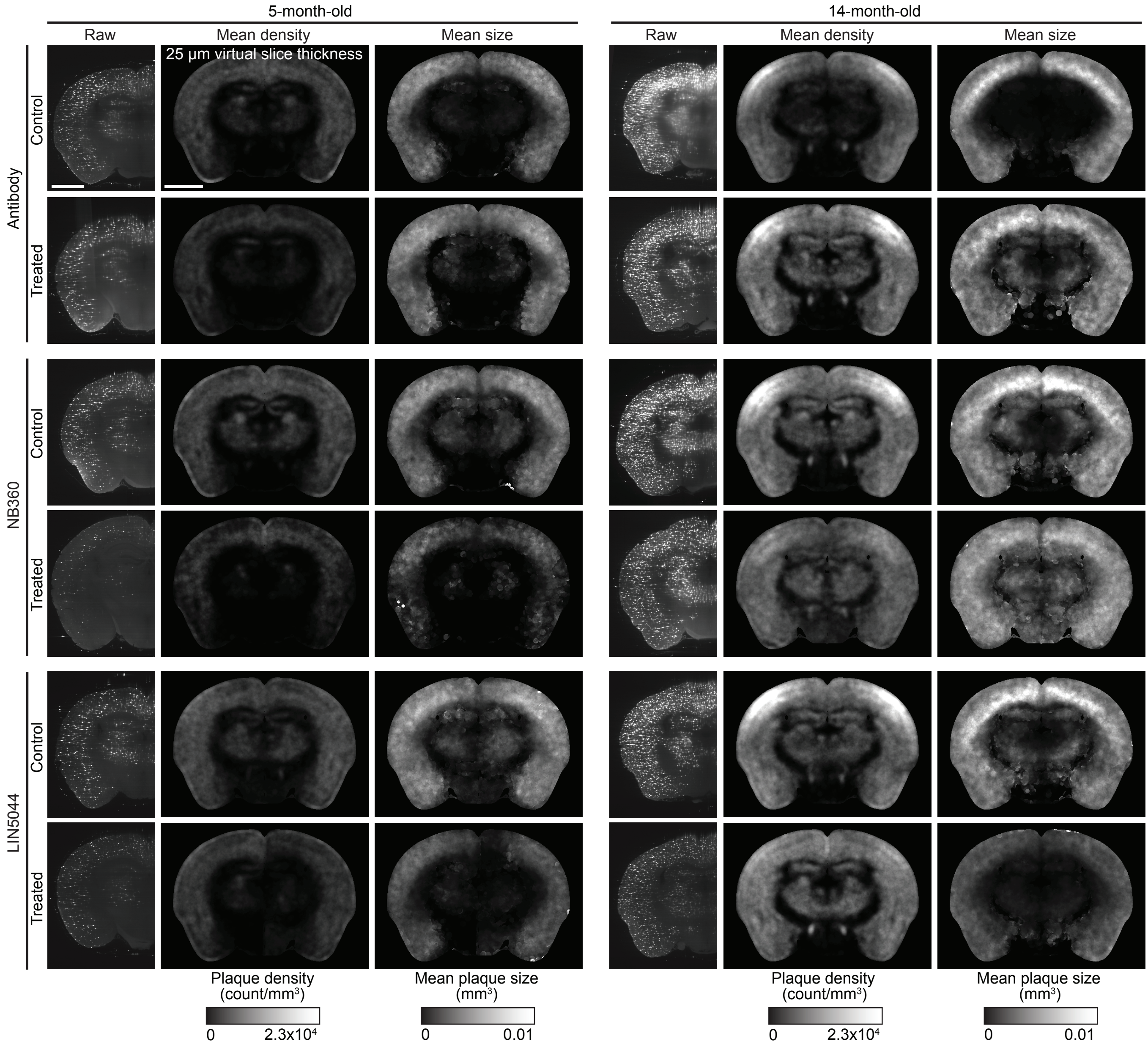

Appendix Figure S5

Appendix Figure S5. Data representations indicate differences between brains of control and treatment cohorts.

Heatmaps of mean plaque densities and sizes are shown across all the treatment and control groups. For each cohort an optical brain slice from lightsheet data is shown (Raw data). Plaque loads in aged mice are much higher than in young mice. The BACE1 inhibitor NB360 treatment suggests reduced plaque counts in 5 – month – old mice. LIN5044 treatment suggests decreased mean plaque size in 14 – month – old mice. In some cases, the optical brain slices do not precisely represent the anatomy of the atlas-registered heatmap-slices. This is because the mounting (and thus the orientation) of the brains in the microscope is variable. Scale bars: 2 mm

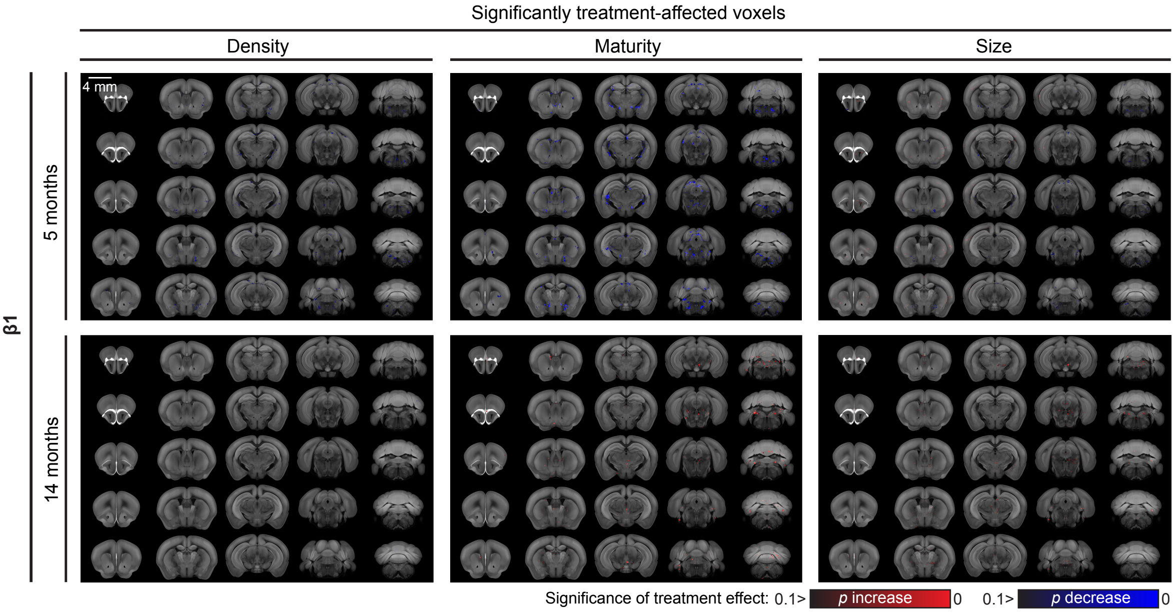

Appendix Figure S6

Appendix Figure S6. The β1 antibody has minimal to no effect on individual voxel plaque count, maturity, or size in 5-month-old and 14-month-old mice.

Heatmap of voxel-based statistics with significant mean plaque size reduction upon β1 antibody treatment in 5-month-old and 14-month-old mice, compared to control. The β1 antibody treatment shows a limited effect across all analyzed metrics.

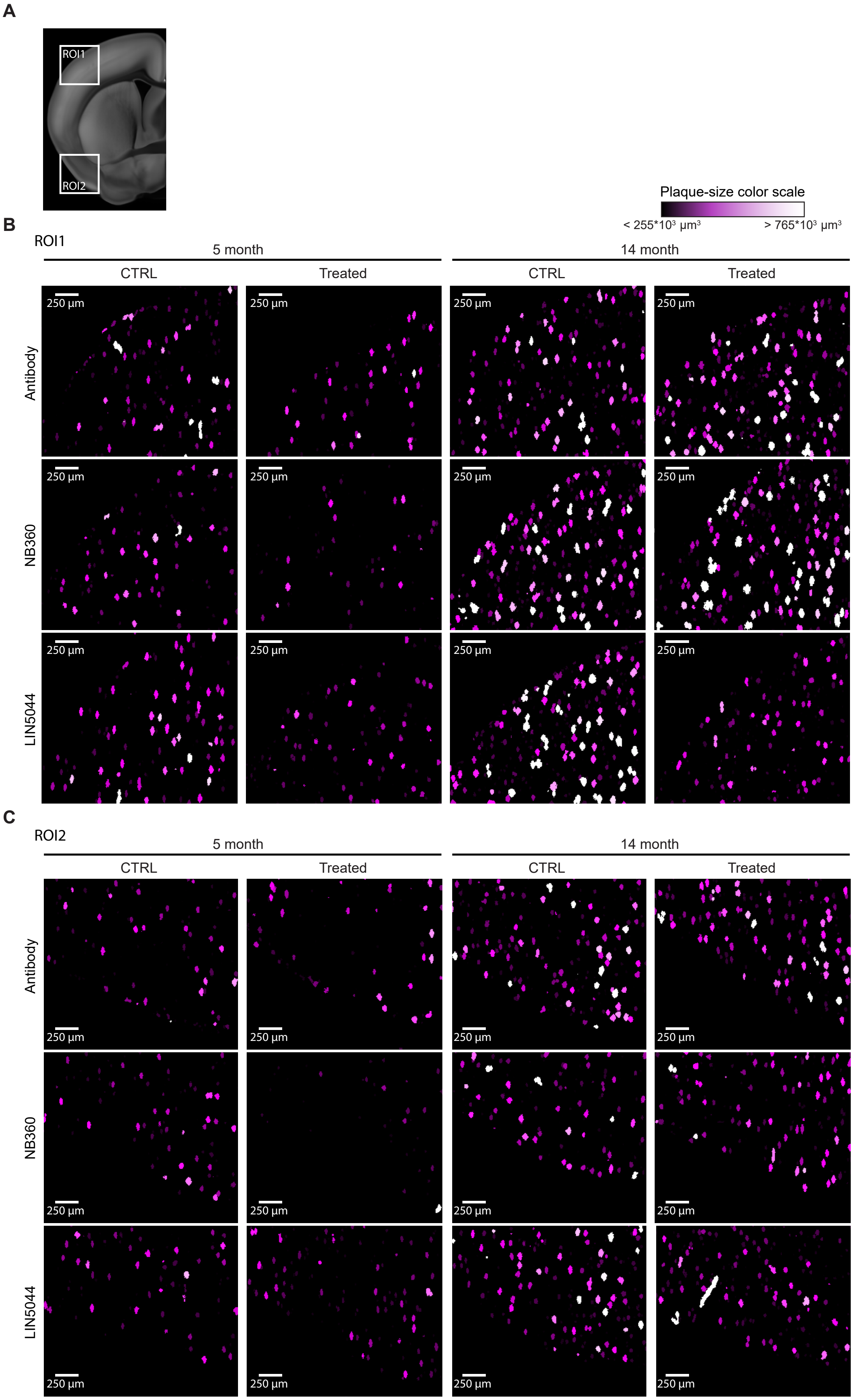

Appendix Figure S7

Appendix Figure S7. Raw data shows differential effects on plaque counts and sizes after amyloid β treatments.

A We randomly picked two optical slices from the cortex (ROI1, ROI2 white inserts) of a control and a treated brain in each treatment cohort. Next, we segmented the plaques and color-coded them such that colors represent 4 equal portions of the total range of plaque sizes (see **Figure 2B**).

B, C NB360 conspicuously reduced numbers and sizes of plaques in young mice, while LIN5044 the number of large plaques in aged mice. The effect in the other treatment cohorts is less obvious.

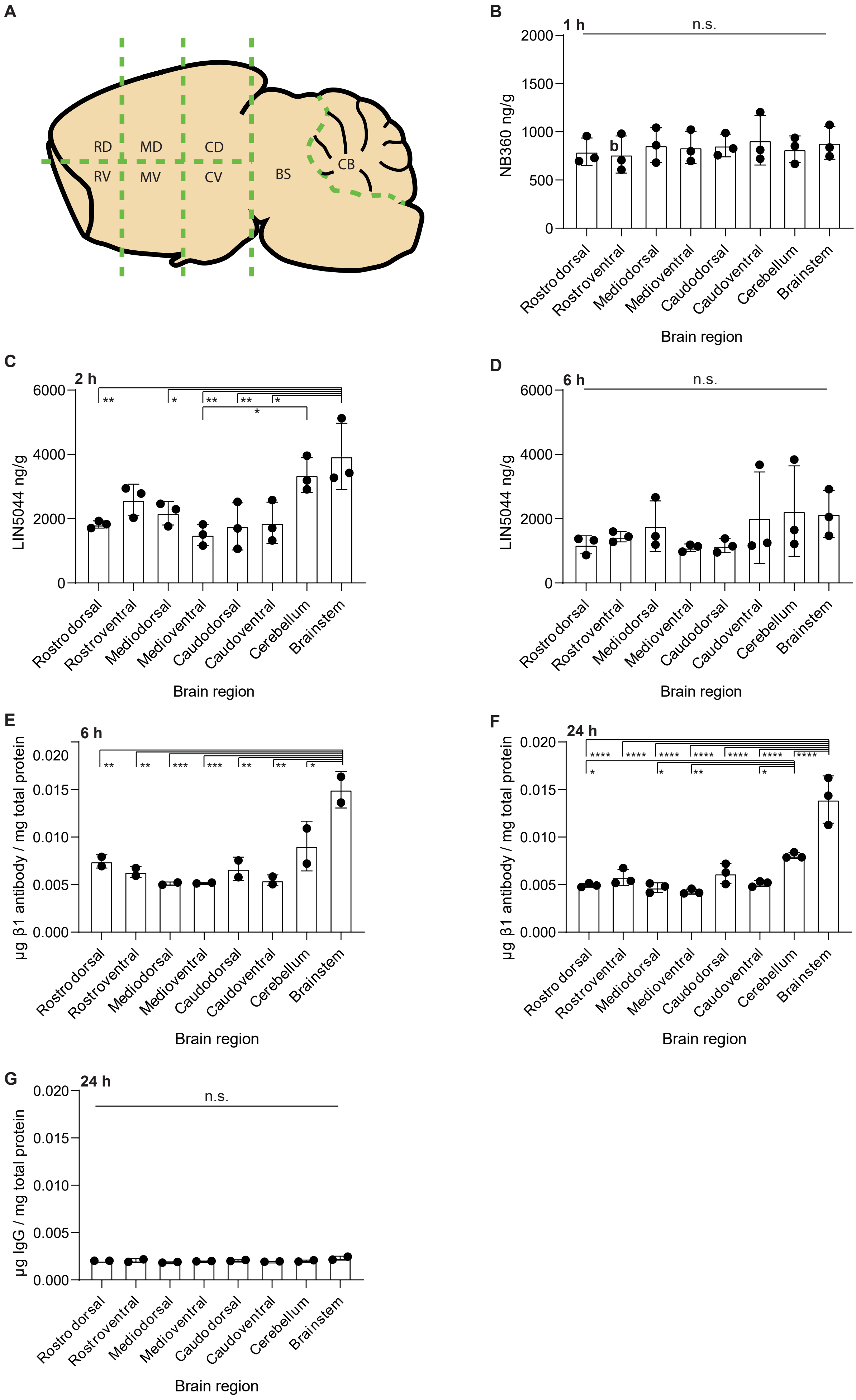

Appendix Figure S8

Appendix Figure S8. Variations in bioavailability across bulk regions do not explain the spatial patterns of drug effectiveness.

A Brains were dissected into eight bulk regions to test regional drug distributions.

B No regional difference in NB360 drug levels detectable after oral administration (n = 3).

C LIN5044 shows significantly higher levels in the brainstem (ranging $0.0023<p<0.0345$) and the cerebellum ($p = 0.0241$) 2 hours after intraperitoneal injection (n = 3).

D No difference detectable at 6 hours with LIN5044 (n = 3).

E, F ELISA measurements of β1 antibody levels after intraperitoneal application showed higher levels in the brainstem (for multiple brain regions ranging $p = 0.0223$ to $p<9\times10^{-4}$ at 6 hours and $p<10^{-4}$ at 24 hours) and the cerebellum (for multiple brain regions ranging $0.0072<p<0.034$ at 24 hours) compared to other regions both at 6 and 24 hours-similarly to LIN5044 (n = 2).

G Pooled non-specific recombinant IgG shows no difference in regional distribution (n = 2). RD – rostrodorsal, RV – rostroventral, MD – mediodorsal, MV – medioventral, CD – caudodorsal, CV – caudoventral, CB – cereballum, BS – brainstem

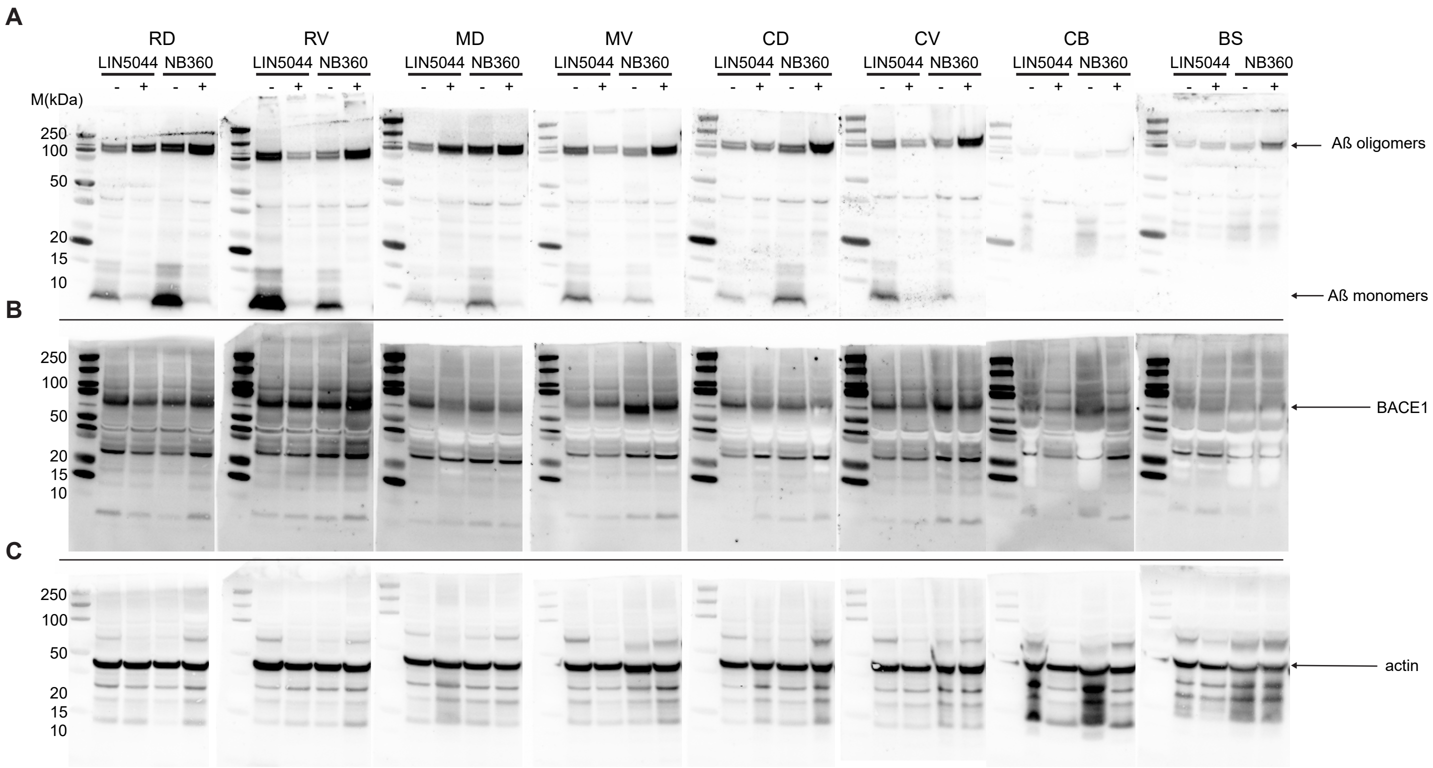

Appendix Figure S9

Appendix Figure S9. Biochemical measurements of Aβ and BACE1 levels across bulk regions do not explain spatial patterns of drug effectiveness.

2-month-old APPPS1 mice were treated with LIN5044 (n=1) or NB360 (n=1) for 3 months, followed by the dissection of one brain hemisphere into 8 bulk regions (**Appendix Figure S8A**).

A Monomeric and oligomeric Aβ species are reduced by both LIN5044 and NB360 treatments in all 8 macroscopic regions when compared to control groups. NB360 has a stronger effect on monomers than LIN5044, while the opposite is true for oligomers.

B BACE1 is detectable in all macroscopic regions without a clear regional predominance. Neither LIN5044 nor NB360 have a reproducible effect on BACE1 levels across macroscopic regions.

C Actin is detectable in all macroscopic regions without a clear regional predominance. RD – rostrodorsal, RV – rostroventral, MD – mediodorsal, MV – medioventral, CD – caudodorsal, CV – caudoventral, CB – cereballum, BS – brainstem

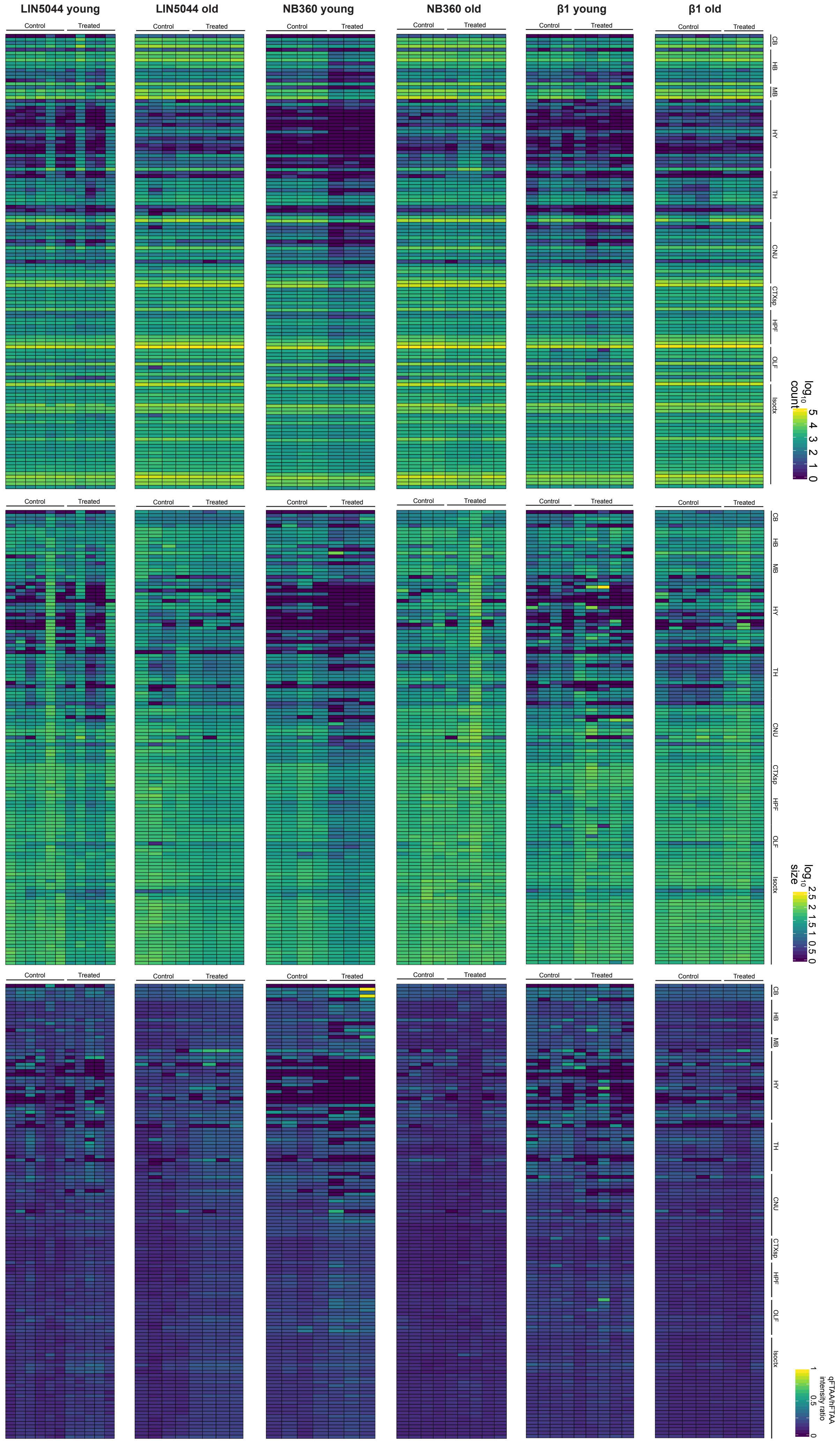

Appendix Figure S10

Appendix Figure S10. Per-subject regional quantification of plaque count, size, and maturity.

The regional quantification of total plaque count, mean plaque size, and mean plaque maturity for individual samples is depicted. Columns represent either a control or treated subject across the various cohorts. Regions are grouped by larger neuroanatomical structures: Isoctx – isocortex, OLF – olfactory areas, HPF – hippocampal formation, CTX sp – cortical subplate, CNU – caudate nucleus, TH – thalamus, HY – hypothalamus, MB – midbrain, HB – hindbrain, CB – cerebellum.
