# Supplementary material for: Whole‐brain microscopy reveals distinct temporal and spatial efficacy of anti‐Aβ therapies
Source: EMBO Mol Med. 2022 Nov 16;15(1):e16789. doi: 10.15252/emmm.202216789 (PMC9832821; doi:10.15252/emmm.202216789)
Supplement: Supplementary file 2 — Expanded View Figures PDF [file EMMM-15-e16789-s006.pdf]

Expanded View Figures

Figure EV1. Homogeneous whole-brain Aβ plaque stain by electrophoretic infusion of qFTAA and hFTAA polythiophenes.

A Plaque maturation states determined with qFTAA vs hFTAA were compared to those identified by N3pE (pyroglutamylated Aβ) vs $Aβ_{17-24}$ (all Aβ moieties). APPPS1 brain sections from one mouse (paraffin, 10 μm) were stained with Aβ-N3pE or Aβ17 24 followed by qFTAA+hFTAA staining. Aβ-N3pE detected plaques similarly to qFTAA, while hFTAA highlighted additional plaques. Conversely, $Aβ_{17-24}$ labeled the entire plaque population similarly to hFTAA (white arrowheads). Scale bars: 100 μm.

B Lightsheet imaging and digital reslicing of cleared whole brains. Plaques were visible in the cortex and in deep diencephalic areas, indicative of homogeneous dye penetration and image acquisition. Plaque maturity was assessed with qFTAA+hFTAA co-staining. As an example, hFTAA identified all plaques (blue arrows), whereas qFTAA stained the cores (white arrows) of more mature plaques. Scale bars: 1 mm.

C Whole-brain hemisphere rendering of an APPPS1 mouse with hFTAA signal. The cerebellum, where the Thy1 promoter is inactive, was unaffected.

Source data are available online for this figure.

© 2022 The Authors

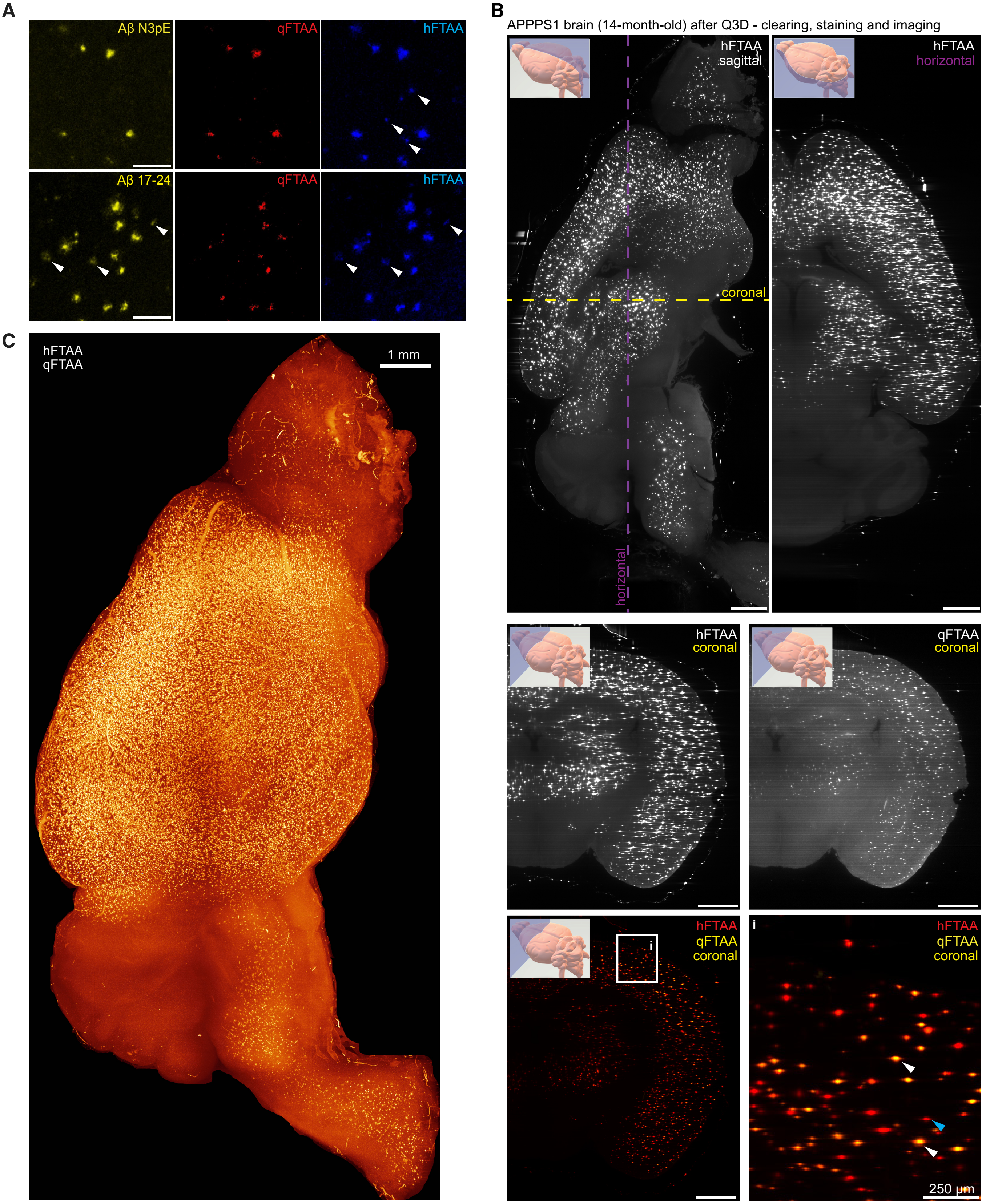
A
Aβ N3pE
qFTAA
hFTAA
Aβ 17-24
qFTAA
hFTAA
B
APPPS1 brain (14-month-old) after Q3D - clearing, staining and imaging
hFTAA
sagittal
coronal
horizontal
hFTAA
horizontal
hFTAA
coronal
qFTAA
coronal
hFTAA
qFTAA
coronal
i
hFTAA
qFTAA
coronal
250 µm
C
hFTAA
qFTAA
1 mm

Figure EV1.

Figure EV2. The β1 antibody has minimal to no effect on neuroanatomical plaque count, maturity, or size in 5-month-old and 14-month-old mice.

Fold-change reduction in various plaque metrics across all brain regions in both 5-month-old and 14-month-old mice, compared with control. The β1 antibody has a minimal effect on plaque-count increase in 5-month-old mice and has no effect in old mice. In 5-month-old mice, significant effects in plaque size reduction occurred mostly in the brainstem. The plaque-maturity change induced by β1 antibody treatment in 5- and 14-month-old mice is very limited. The plaque-size change induced by β1 antibody treatment in 5-month-old mice is very limited and absent in 14-month-old mice. The plaque-maturity change induced by β1 antibody treatment in 5- and 14-month-old mice is very limited. Brain regions with a significant treatment effect ($P < 0.05$) are shaded gray. Isoctx, isocortex; OLF, olfactory areas; HPF, hippocampal formation; CTX sp, cortical subplate; CNU, caudate nucleus; TH, thalamus; HY, hypothalamus; MB, midbrain; HB, hindbrain; CB, cerebellum.

 © 2022 The Authors

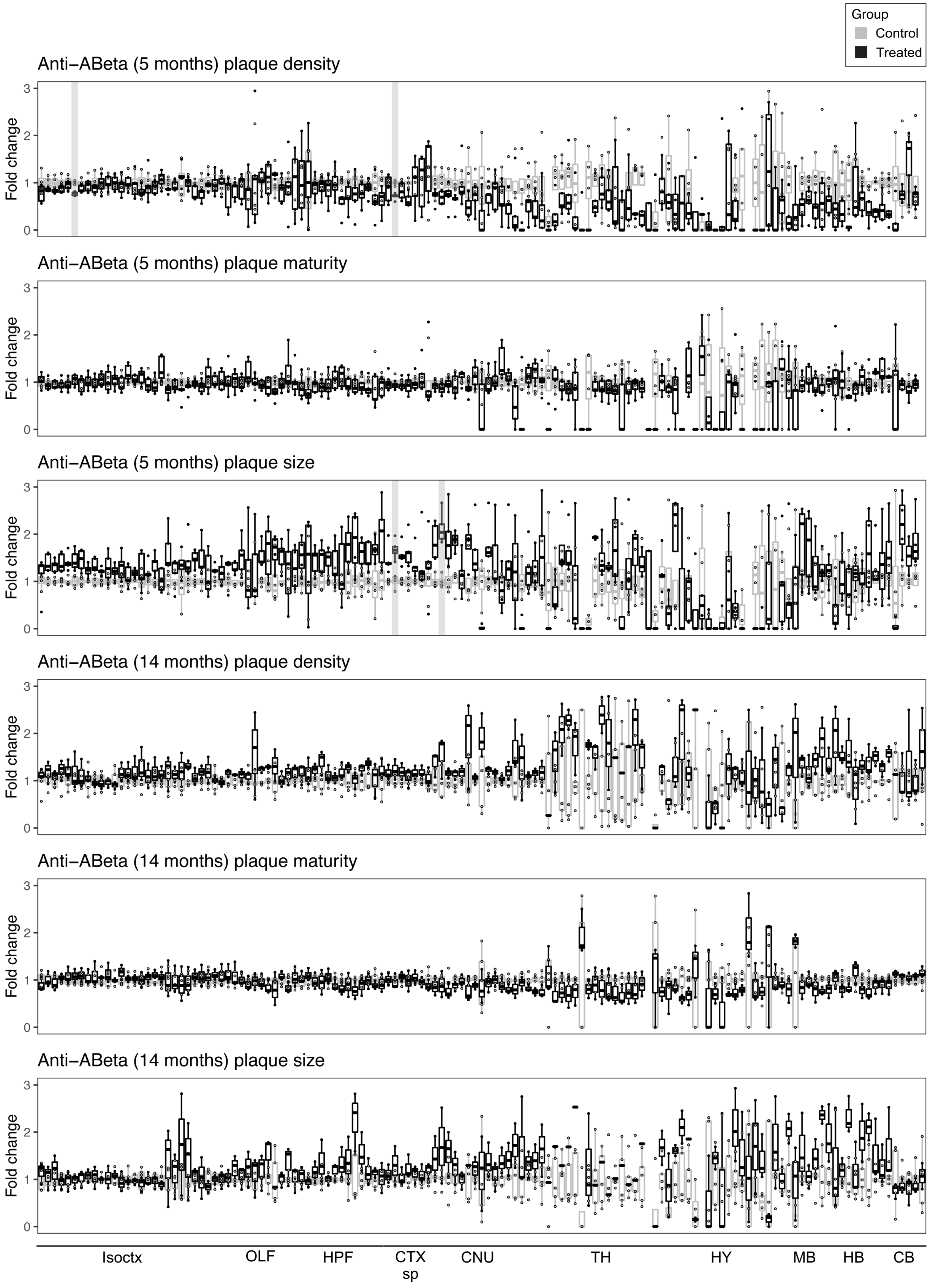
Group
Control
Treated
Anti−ABeta (5 months) plaque density
Anti−ABeta (5 months) plaque maturity
Anti−ABeta (5 months) plaque size
Anti−ABeta (14 months) plaque density
Anti−ABeta (14 months) plaque maturity
Anti−ABeta (14 months) plaque size
Fold change
0
1
2
3
Isoctx
OLF
HPF
CTX sp
CNU
TH
HY
MB
HB
CB

Figure EV2.

Figure EV3. BACE1-inhibition induced conspicuous plaque count reduction in 5-month-old mice, but not in 14-month-old mice.

Fold-change reduction in various plaque metrics across all brain regions in both 5-month-old and 14-month-old mice. BACE1-inhibition induces considerable reduction in both plaque count and plaque size in many anatomical regions in 5-month-old mice, but not in 14-month-old mice. Plaque maturity change by BACE1-inhibition in 5-month-old mice shows both region-dependent maturity increase and decrease, while there is no effect at 14-months. Brain regions with a significant treatment effect ($P < 0.05$) are shaded gray. Isoctx, isocortex; OLF, olfactory areas; HPF, hippocampal formation; CTX sp, cortical subplate; CNU, caudate nucleus; TH, thalamus; HY, hypothalamus; MB, midbrain; HB, hindbrain; CB, cerebellum.

 © 2022 The Authors

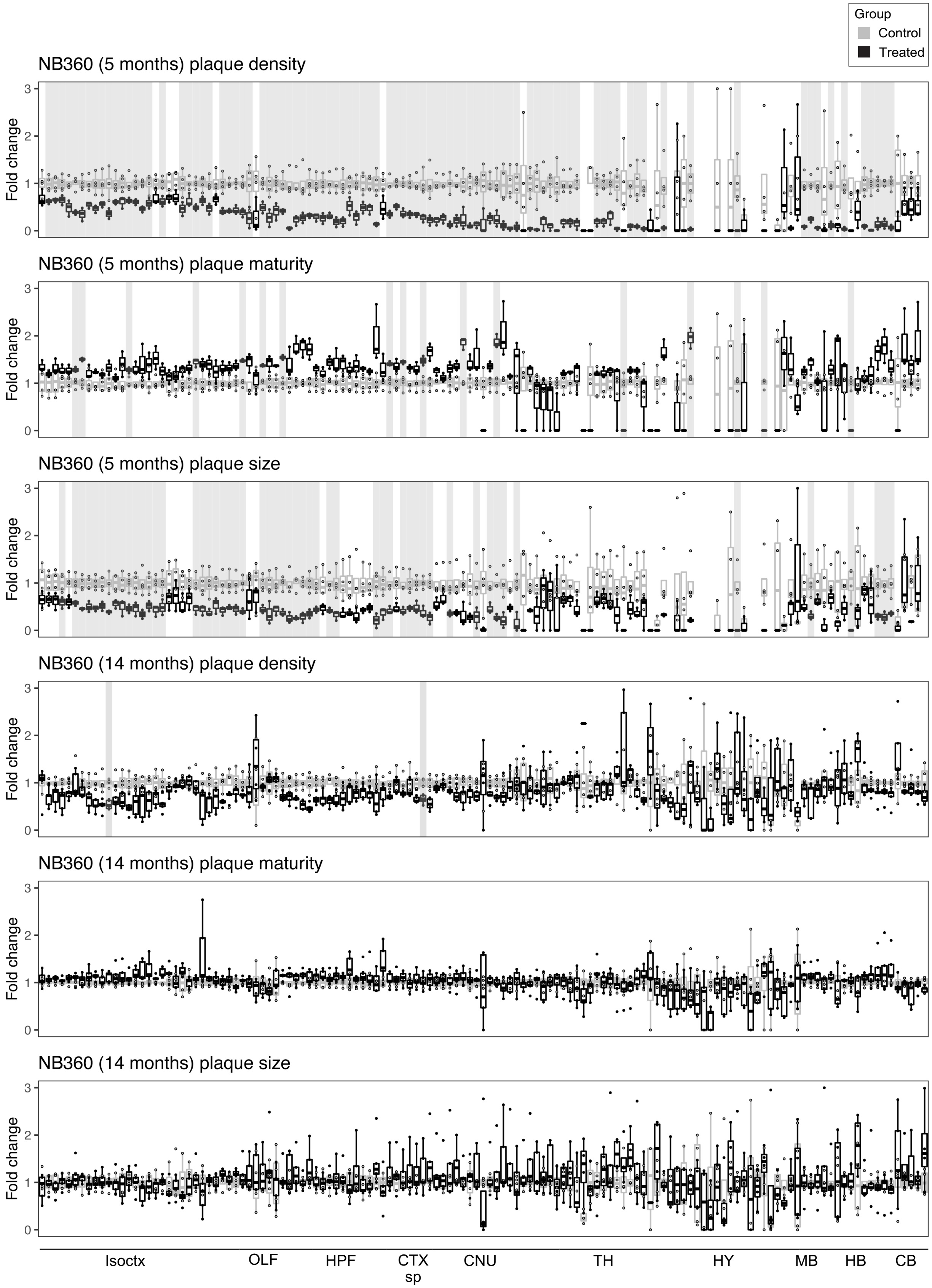
Group
Control
Treated
NB360 (5 months) plaque density
NB360 (5 months) plaque maturity
NB360 (5 months) plaque size
NB360 (14 months) plaque density
NB360 (14 months) plaque maturity
NB360 (14 months) plaque size
Fold change
0
1
2
3
Isoctx
OLF
HPF
CTX sp
CNU
TH
HY
MB
HB
CB

Figure EV3.

Figure EV4. Trend in plaque-count reduction in few anatomical regions after LIN5044 treatment in 5- and 14-month-old mice.

A To see whether LIN5044 induces artifacts into the plaque-maturity analysis APPPS1 mice were injected with a single-dose PBS ($n = 1$) or LIN5044 (0.4 mg in 100 µl PBS; $n = 1$). Next, emission intensities used for plaque maturity analysis (at wave lengths 498–520 and 565–605 nm) were measured in 10 plaques in 3 slices per animal with a confocal microscope. Plaques of LIN5044 and PBS injected mice showed no difference in fluorescent emissions.

B Fold-change reduction in various plaque metrics across all brain regions in both 5-month-old and 14-month-old mice, compared with control. Plaque-count reduction is not significant but shows a trend in 14-month-old mice. Mean plaque-sizes are significantly reduced in some anatomical regions after LIN5044 treatment in 5-month-old mice, and in cortical areas of 14-month-old mice. Plaque maturity change by LIN5044 in 5-month-old mice shows some neuroanatomical regions with mean maturity increase, and a more widespread regional increase at 14-months in cortical areas. Brain regions with a significant treatment effect ($P < 0.05$) are shaded gray. Isoctx, isocortex; OLF, olfactory areas; HPF, hippocampal formation; CTX sp, cortical subplate; CNU, caudate nucleus; TH, thalamus; HY, hypothalamus; MB, midbrain; HB, hindbrain; CB, cerebellum.

© 2022 The Authors

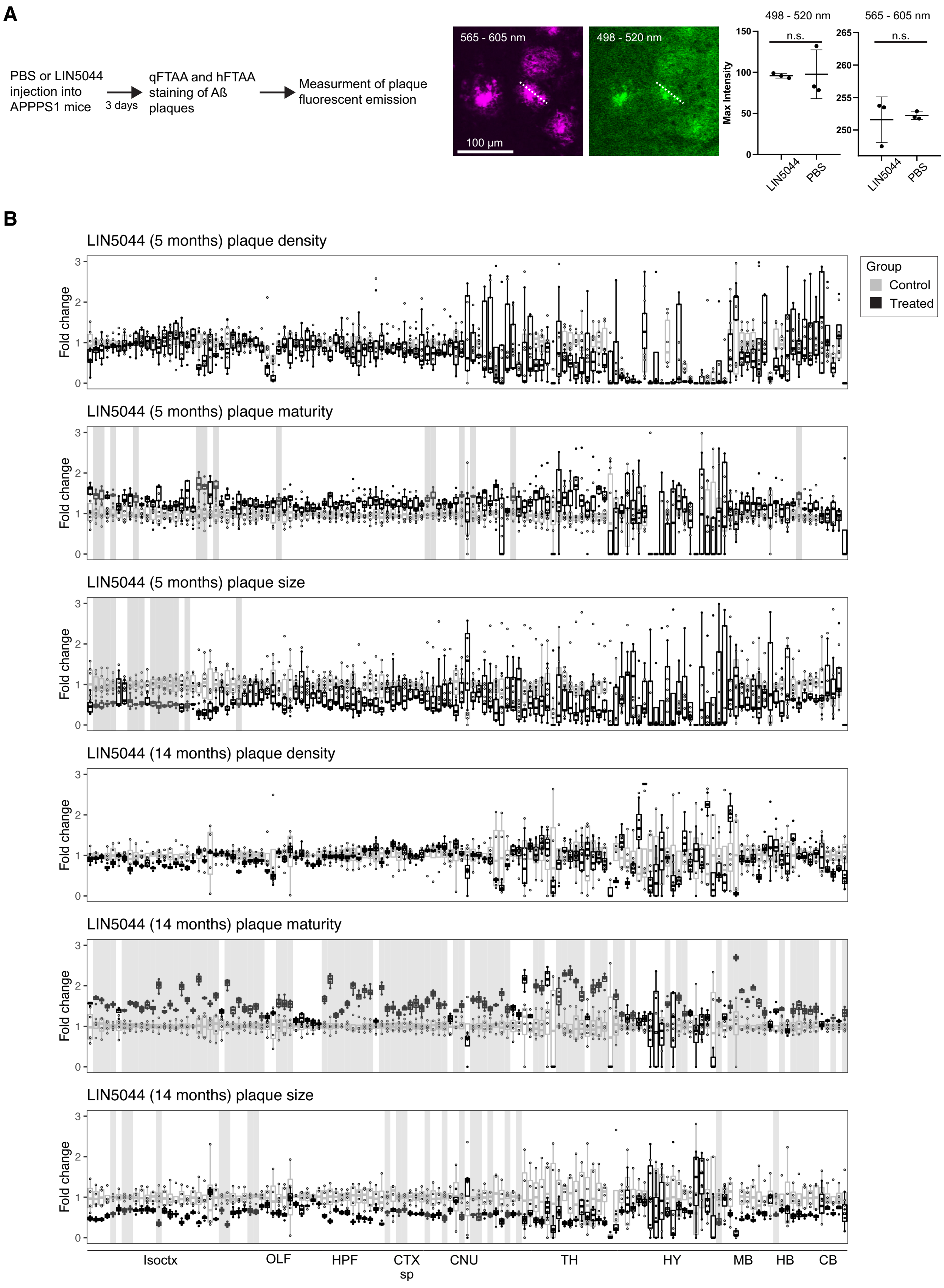
A
PBS or LIN5044 injection into APPPS1 mice
3 days
qFTAA and hFTAA staining of Aß plaques
Measurment of plaque fluorescent emission
565 - 605 nm
498 - 520 nm
100 µm
n.s.
Max Intensity
LIN5044
PBS
B
LIN5044 (5 months) plaque density
LIN5044 (5 months) plaque maturity
LIN5044 (5 months) plaque size
LIN5044 (14 months) plaque density
LIN5044 (14 months) plaque maturity
LIN5044 (14 months) plaque size
Fold change
Group
Control
Treated
Isoctx
OLF
HPF
CTX sp
CNU
TH
HY
MB
HB
CB

Figure EV4.

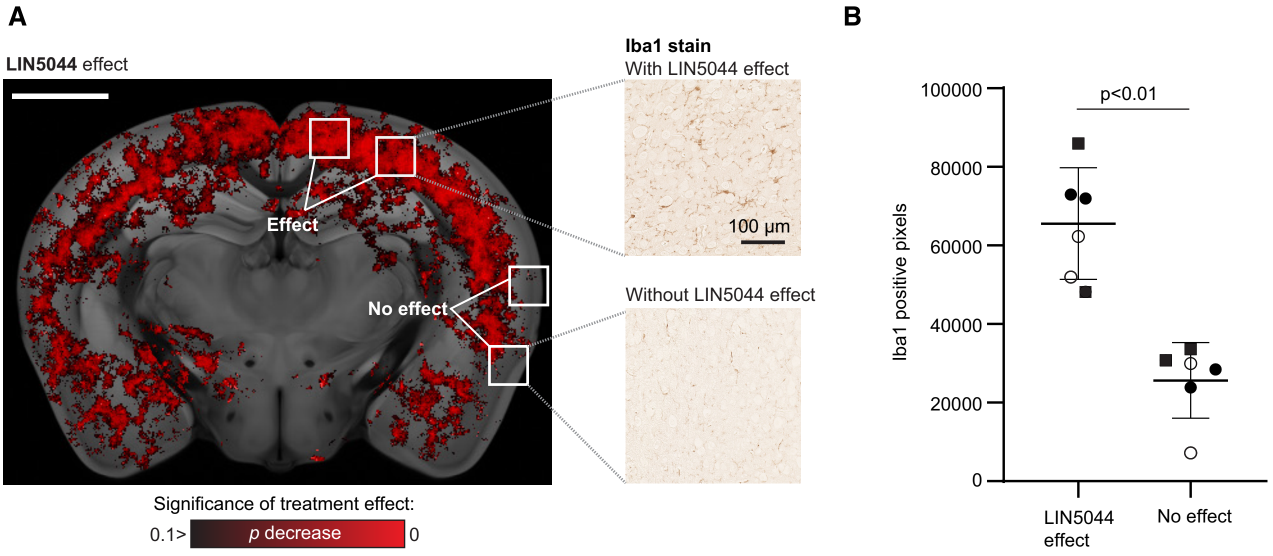

Figure EV5. Histological sectioning and Iba1 labeling validates increased microglia density in regions where LIN5044 was more effective in reducing plaque size.

A Regions of interest were selected based on voxel-based statistics of LIN5044 efficacy in reducing plaque size in 14-month-old mice. Microglia density was measured in regions displaying both strong or absent LIN5044 efficacy (3 three wild-type mice, 3 slices/mouse). Scale bar: 2 mm

B Regions showing strong LIN5044 effects contained more microglia (each distinct symbol represents one mouse).

 © 2022 The Authors
