# Supplementary material for: Whole‐brain microscopy reveals distinct temporal and spatial efficacy of anti‐Aβ therapies
Source: EMBO Mol Med. 2022 Nov 16;15(1):e16789. doi: 10.15252/emmm.202216789 (PMC9832821; doi:10.15252/emmm.202216789)
Supplement: Supplementary file 6 [file EMMM-15-e16789-s002.pdf]

EMBO Molecular Medicine

Whole-brain microscopy reveals distinct temporal and spatial efficacy of anti-Aβ therapies

Daniel Kirschenbaum, Ehsan Dadgar-Kiani, Francesca Catto, Fabian Voigt, Chiara Trevisan, Oliver Bichsel, Hamid Shirani, Peter Nilsson, Karl Frontzek, Paolo Paganetti, Fritjof Helmchen, Jin Hyung Lee, and Adriano Aguzzi
DOI: 10.15252/emmm.202216789

Corresponding author(s): Jin Hyung Lee (ljinhy@stanford.edu) , Adriano Aguzzi (adriano.aguzzi@usz.ch)

Review Timeline:

Transfer from Review Commons:	24th Aug 22
Editorial Decision:	21st Sep 22
Revision Received:	21st Oct 22
Accepted:	24th Oct 22

Review COMMONS

Editor: Zeljko Durdevic

Transaction Report: This manuscript was transferred to EMBO Molecular Medicine following peer review at Review Commons.

Review #1

1. Evidence, reproducibility and clarity:

Evidence, reproducibility and clarity (Required)

The authors develop a rapid tissue clearing method and staining protocol supported by electrophoresis and apply their method to map out Ab plaques.

As my expertise is not in Alzheimer's Disease I will here only comment on the tissue clearing and analysis methods as agreed with the editor.

The authors develop a rapid tissue clearing method and staining protocol supported by electrophoresis and apply their method to map out Ab plaques.

The paper is well written, and the results interesting. Particularly the region specificity of the treatments as well as the alignment of the observed spatially distributed effect sizes with existing transcriptomics data to reveal possible mechanisms behind the observations are interesting results.

The main criticism I have lies on the analysis side, in that the classifiers to detect plaques need to be retrained for each sample (or at least with changes in imagining conditions).
Systematic preprocessing of the data might circumvent this downside or otherwise this should be discussed clearly in the manuscript.

I have the following additional technical comments:

1. From the text and Figure 1 it is unclear to me how the electric field does not 'escape' on top and the bottom of the tissue ?
In addition it is unclear how field inhomogeneities might influence the clearing and staining process particularly near the insulting layers that might also deform the tissue itself ?
2. When testing for the number of plaques between the developed method and the test in restraining slices it would be interesting to know how many of the counts are missed in one sample and what is the overlap of detected plaques as well as their difference in size estimates.
While it seems ok in this study, in principle the counts could be similar but complete different populations of plaques are actually detected in each round.
3. Regarding the analysis, it would be useful to see how the trained classifiers perform on a hand annotated region not used for training to obtain error estimates on those counts. Those errors should then be also taken into consideration in the statistical analysis. How do significance levels change when including those ?
- As mentioned in the main comment, a possible downside of this approach that seems to do no preprocessing of the raw data might be that the classifiers need to be retrained for each sample if background /foreground intensities change (which typically is the case).
Thus, error rates on the numbers might be even harder to estimate making statistical statements when comparing the data sets less powerful.

The authors do not seem to discuss these potential sources of error in their manuscript.

In addition, the staining intensities may vary with distance from brain surface or along the direction of the dye passing through. It would be helpful to see samples from various areas and how the detection algorithm perform son those to ensure region specificity is not an artifact of inhomogeneous staining/imagining.
4. Another source of error might be the alignment process to the reference atlas. Methods like Clarity typically need additional care, while other methods like iDISCO are more robust against deformations.
It thus would be good to discuss this aspect in the paper and/or try to quantify possible alignment errors as well.
5. A study using tissue clearing and mapping of plaques in Alzheimer's has been published using iDISCO clearing followed by analysis.
https://pubmed.ncbi.nlm.nih.gov/27425620/
It surprises me that the authors do not mention this study at all as it is closely related. In my view at least a comparison between the proposed and already developed method would be in to help judging the differences (advantages / disadvantages) between both 'equivalent' methods
6. The results on effect sizes are interesting. However, it could be helpful besides providing p-values to also provide actual variation of counts (std) encountered in e.g., wild type vs treatment groups in various areas. I.e., are certain areas highly variable in plaque expression in untreated mice already. Which areas show highest variation? If there are strong differences in variation what are possible reasons for it.
7. It could be interesting to include a combined LIN5044 and NB360 treatment into the study to see if those complement each other or actually might interact in non-linear ways in certain areas.

2. Significance:

Significance (Required)

see report above for a closely related earlier study not discussed in the manuscript.
a comparison between both methods is needed.

The results of the study particular alignment of the effect sizes with transcriptomics data is very interesting as it allows to generate hypothesis on the underlying mechanisms.

for more details see report above.

3. How much time do you estimate the authors will need to complete the suggested revisions:

Estimated time to Complete Revisions (Required)

(Decision Recommendation)

Cannot tell / Not applicable

4. Review Commons values the work of reviewers and encourages them to get credit for their work. Select 'Yes' below to register your reviewing activity at Publons; note that the content of your review will not be visible on Publons.

Reviewer Publons

Yes

Review #2

1. Evidence, reproducibility and clarity:

Evidence, reproducibility and clarity (Required)

In this manuscript, Kirscherbaum et al described 3 major science advance they accomplished:

1. Developed a novel method to globally evaluate amyloid plaque load across brain of an amyloidosis model with high resolution.
2. Compared spatial changes of amyloid plaque density, size and maturity after treatment of 3 different anti-amyloid molecules.
3. Analyzed spatial correlation between gene expression and amyloid plaque changes.

The findings are novel and interesting (most data were published in a biorxiv last year). I support publication of the manuscript after the authors address several of my points.

Major points:

1. The author use tissue clearing and qFTAA/hFTAA labeling of whole brain for plaque labeling. That is the basis of the whole manuscript. How does the labeling of qFTAA and hFTAA comparing with widely used methoxy-X04 or X34 labeling of plaques? Methoxy X04 is BBB penetrant and widely used to label dense core plaques (Klunk et al 2002 PMID: 12230326). It will be good for the authors to compare their method with methoxy labeling.
2. the authors tried to make a point that whole brain electrophoretic staining with polythiophene can label plaque equally comparing with traditional staining. However, the two panels in figure 1D showed changes of several plaques either by location or by intensity (lower right, middle left etc) which is concerning in term of consistency of the electrophoresis labeling. Based on numbers in the manuscript, the change is minimal between the two conditions, but the picture showed different morphology or number for at least a few percent of the plaques. I would like the authors do more analysis and show a few more pictures in a supplementary figure.
3. the lack of obvious plaque changes in mice after 3m antibody treatment is a little surprising since many papers showed immunotherapy is able to reduce amyloid plaque load (antibodies like 3D6, mE8). The authors evaluated PK but did not discuss extensively the dose they select. What is the basis for that dose? One thing can be done is a target engagement study with biotinylated antibody. The authors know there is exposure of the antibody in the tissue but they did not test whether the antibody engage with Abeta.
4. the spatial correlation of gene expression and plaque change is very intriguing and potentially important to understand brain microenvironment. But I am not sure the data fully make sense. For example, the BACE1 mRNA is mainly in the cytoplasm but BACE1 protein is often transported and localized into neurites. Does that mean the cell body BACE is the main driver for Abeta production? if not, the spatial correlation will be difficult to explain.

Minor points:

1. Several figures (e.g. Figure 2F) is difficult to read with small font and panel.
2. Figure 2D can be put in supplemental.
3. The difference of LIN5044's impact on intracellular prion protein and extracellular abeta can be discussed a bit more.

2. Significance:

Significance (Required)

Anti-amyloid therapies have been the front runners for Alzheimer's disease clinical trials, with billions of dollars already spent. It is important to understand the similarities and difference among these therapies. The manuscript tried to address these questions in a comprehensive and age related way in prevention and treatment paradigms. Although I still have some questions and concerns about the methodology, the data package can probably stimulate the broad AD research community to think and design anti-amyloid studies with different disease stages, combination approaches and endpoints in mind. The spatial gene expression analysis is also quite interesting. But the future key experiment will need to done to understand the spatial correlation between mRNA expression and protein distribution in a similar voxel based analysis.

3. How much time do you estimate the authors will need to complete the suggested revisions:

Estimated time to Complete Revisions (Required)

(Decision Recommendation)

Between 1 and 3 months

4. Review Commons values the work of reviewers and encourages them to get credit for their work. Select 'Yes' below to register your reviewing activity at Publons; note that the content of your review will not be visible on Publons.

Reviewer Publons

Yes

Review #3

1. Evidence, reproducibility and clarity:

Evidence, reproducibility and clarity (Required)

Summary:

The authors developed an improved tissue clearing method based on a focused electrophoretic clearing. They used this method to analyze the effects of anti Aß immunotherapy (ß1 antibody), BACE1 inhibition (NB360), and amyloid intercalator (LIN5044) treatments in young or old APP/PS1 mice. Effects on amyloid plaque density, structure

(presence of a dense core) and size were studied.

The main conclusions were that the immunotherapy had no effect. The BACE1 antagonist decreased the number and size of plaques but only when applied to young mice. The amyloid-binding compound LIN5044 did not change plaque number but increased their compaction when applied in old mice.

Alignment with published spatial transcriptomic indicated that the effect of LIN5044 on plaque compaction occurred in association with expression of Cx3c11, a microglial chemoattractant mostly expressed by neurons. Accordingly, the authors found that areas where LIN5044 has a stronger effect had more microglia.

Major comments:

In the Abstract, Introduction, ... the authors argue that clinical trial failures of Aß-therapies could be due to the fact that specific treatments may have regional or temporal heterogeneity of efficacy. However, Aß immunotherapy is very efficient to remove plaques in human patients, even so it does not prevent cognitive deterioration. Thus, the absence of clinical effects is not due to a poor efficiency for plaque removal. This argument should be removed from all the manuscript. Further, BACE antagonist therapy were stopped because of side effects.

As it is known that immunotherapy against Aß has strong effects on plaques, both in human AD patients and in transgenic mouse models, the surprisingly modest (at best) effect of immunotherapy in the present study was likely a technical issue. Thus, the data concerning Aß immunotherapy should be removed.

In the quantifications the authors frequently indicated the significance of the results without showing the magnitude of the effects (Fig. 2). When the fold changes were presented (Suppl Fig 6, 8 and 9), there was no indication of the relative level of plaque parameters according to the various brain regions analyzed. Further, in Fig 2 as in Suppl Fig 6, 8 and 9 there were many samples per region, and the borders between regions were not indicated. I would suggest to make graphs with the mean values per region, to allow an easier visualization of the effects.

Minor comments:

Suppl Fig 12: was it really n=1 as indicated in the Legend? If so, it should be removed. Also, it is not clear why no difference in Aß abundance was detected between the 8 brain regions (Results p. 8), given the major differences in plaque density.

In the Discussion p. 9, it is indicated that "... both LIN5044 and NB360 cleared plaques ...". However, in Fig 2 LIN5044 did not modify plaque density.

The mention of ventrocaudal and dorsorostral gradients should be removed. Indeed, ventral and caudal (or dorsal and rostral) regions are eventually co-localized, and it is therefore not possible to establish a gradient between them.

p. 10 "... evidence that LIN5044 reduces additive plaque growth". Is it possible that LIN5044 increases plaque compaction?

Methods p. 12 Tissue clearing. Clearing time varied between 6 and 14 hours. What were the criteria to stop the clearing?

The color codes for increasing or decreasing effects are opposite in Figs 2 and 3.

Legend of Suppl Fig 4: It seems to me that the "C, " in the various parentheses should be removed.

2. Significance:

Significance (Required)

The study is very interesting and brings new methodological tools. Since the failure of several clinical trials in Alzheimer's disease targeting the amyloid pathology, it remains important to understand the reasons of these failures. Here the authors bring new hypothesis regarding the maturity of amyloid deposits, their localization and specific treatments.

3. How much time do you estimate the authors will need to complete the suggested revisions:

Estimated time to Complete Revisions (Required)

(Decision Recommendation)

Between 1 and 3 months

4. Review Commons values the work of reviewers and encourages them to get credit for their work. Select 'Yes' below to register your reviewing activity at Publons; note that the content of your review will not be visible on Publons.

Reviewer Publons

Yes

Manuscript number: RC-2022-01485
Corresponding author(s): Jin Hyung Lee

1. General Statements [optional]

This section is optional. Insert here any general statements you wish to make about the goal of the study or about the reviews.

We sincerely thank the Reviewers for their helpful comments that helped us significantly improve our revised manuscript and figures.

We have added two new supplementary figures (Fig. S3, S15) and modified four existing figures (Fig. 1, 2, S5, and S13), and made significant changes to our methods and discussion sections. In these new and updated figures, we analyzed the sensitivity of our conclusions to the plaque segmentation accuracy (Fig. S5B) and have highlighted quantitative methods we developed for assessing whole brain registration results (Fig. S5D). Reviewer also asked for a comparison of our results with other amyloid labeling methods. To address this, we introduced a new figure (Fig. S3) quantitatively comparing our rapid clearing method with a conventional passive method. We have also now cited previous publications that compared and validated our clearing method. (These updates addressed 12 out of 16 major comments)

There was also a comment caused by a misunderstanding, particularly regarding the technical approaches used for image segmentation and the classification of pathology. In this study, we have developed one machine learning classifier that is generalizable across all samples (we did not use separate classifiers for each sample). We have since updated our methods section to clearly state this. (This addressed 1 out of 16 major comments)

Reviewers asked for a comparison between our method and solvent-based tissue clearing methods. While these are interesting comparisons to pursue for future studies, our primary goal for this study was to compare the effect of different anti-amyloid-beta drugs across the brain, for which we developed an fast tissue clearing pipeline that can quantify amyloid beta plaques. This was very useful given the large number (12 groups, 4-5 brains/group) of brains we had to clear and image for comparison. However, to ensure that the method could provide accurate results,

we did compare our pipeline with existing and validated passive tissue clearing methods. (This addressed 3 out of 16 major comments.)

2. Point-by-point description of the revisions

This section is mandatory. Please insert a point-by-point reply describing the revisions that were already carried out and included in the transferred manuscript.

Reviewer #1 (Evidence, reproducibility and clarity (Required)):

The authors develop a rapid tissue clearing method and staining protocol supported by electrophoresis and apply their method to map out Ab plaques.

As my expertise is not in Alzheimer's Disease I will here only comment on the tissue clearing and analysis methods as agreed with the editor.

The authors develop a rapid tissue clearing method and staining protocol supported by electrophoresis and apply their method to map out Ab plaques.

The paper is well written, and the results interesting. Particularly the region specificity of the treatments as well as the alignment of the observed spatially distributed effect sizes with existing transcriptomics data to reveal possible mechanisms behind the observations are interesting results.

The main criticism I have lies on the analysis side, in that the classifiers to detect plaques need to be retrained for each sample (or at least with changes in imaging conditions). Systematic preprocessing of the data might circumvent this downside or otherwise this should be discussed clearly in the manuscript.

I have the following additional technical comments:

1.) From the text and Figure 1 it is unclear to me how the electric field does not 'escape' on top and the bottom of the tissue ? In addition it is unclear how field inhomogeneities might influence the clearing and staining process particularly near the insulting layers that might also deform the tissue itself ?

Response: We agree that the insulation cannot be perfect and that some current will inevitably flow around the tissue. Nonetheless, this technique better focuses the current (and detergent) flow to the tissue than the original CLARITY protocol, and it improves the efficiency of the clearing process as shown in our data in Fig. 1B-C. We have added some additional schematic of the brain to Figure 1A to clarify. We agree that a more detailed analysis of the electric field distribution

and a time-resolved analysis of the clearing heterogeneity in the tissue would be of interest for future studies. Since the statistical analysis was performed across the brains processed with the same protocol that has the same electric field inhomogeneities, we believe that the electrical field inhomogeneities would not affect any of the conclusions we present. Also, as depicted in new Figure S15, the plaque counts in control mice measured using our proposed clearing method showed relatively low variability, so we do not expect the inhomogeneities to impact the plaque counts or sizes we measured in this study.

2.) When testing for the number of plaques between the developed method and the test in restraining slices it would be interesting to know how many of the counts are missed in one sample and what is the overlap of detected plaques as well as their difference in size estimates. While it seems ok in this study, in principle the counts could be similar but complete different populations of plaques are actually detected in each round.

Response: The Authors thank the Reviewer for bringing up this important point. This calibration experiment was originally a qualitative test, and we found that the difference between the images was minimal by simple visual inspection. However, the re-staining, re-mounting, and re-focusing can result in distortions of the tissue. To better evaluate this, we have included an additional supplementary figure with more images and quantitative comparisons (see Fig. S3). We also emphasized this in the text: "Slight differences in plaque counts and morphology were a result of physical distortions of the slices during passive staining, and due to focal shifts during re-imaging of the free-floating slices." (p.4).

3.) Regarding the analysis, it would be useful to see how the trained classifiers perform on a hand annotated region not used for training to obtain error estimates on those counts. Those errors should then be also taken into consideration in the statistical analysis. How do significance levels change when including those ?

Response: The Authors thank the Reviewer for bringing up this important point. We have updated Fig. S5B to include the trained classifier's error alongside the statistical whole brain analysis. We found that all our prominent findings for NB360 and LIN5044 had effects within the precision and recall rates of our classifier.

$) As mentioned in the main comment, a possible downside of this approach that seems to do no preprocessing of the raw data might be that the classifiers need to be retrained for each sample if background /foreground intensities change (which typically is the case). Thus, error rates on the numbers might be even harder to estimate making statistical statements when comparing the data sets less powerful.

Response: The Authors would like to sincerely apologize for any potential misunderstanding we might have caused. We only trained a single classifier utilized for the analysis of all brains. We have since updated the methods section of our manuscript to more clearly indicate this.

The authors do not seem to discuss these potential sources of error in their manuscript. In addition, the staining intensities may vary with distance from brain surface or along the direction of the dye passing through. It would be helpful to see samples from various areas and how the detection algorithm perform son those to ensure region specificity is not an artifact of inhomogeneous staining/imagining.

Response: In Fig. S4, we show images recorded with Mesospim spanning the entire brain, including deep regions. We believe these images depict how well plaques are captured throughout the brain. We disagree that a gradient in staining or imaging quality could lead to the regional differences we see. Such artifacts would affect both the treated and control groups. It would be hard to explain the very distinct plaque clearance patterns upon the three treatment modalities. This would mean that clearing artefacts only affect specific experimental groups.

4.) Another source of error might be the alignment process to the reference atlas. Methods like Clarity typically need additional care, while other methods like iDISCO are more robust against deformations.
It thus would be good to discuss this aspect in the paper and/or try to quantify possible alignment errors as well.

Response: We thank the Reviewer for raising this point. We have since analyzed in more detail the transformations and similarity metrics used in the registration process for each brain. In Fig. S5D we now depict the linear expansion of cleared brains along each axis (ML, DV, AP), showing consistent tissue shrinkage along the ML and AP axes. We also depict the convergence of the metric (mutual information) used for registration.

5.) A study using tissue clearing and mapping of plaques in Alzheimer's has been published using iDISCO clearing followed by analysis.
https://pubmed.ncbi.nlm.nih.gov/27425620/
It surprises me that the authors do not mention this study at all as it is closely related. In my view at least a comparison between the proposed and already developed method would be in to help judging the differences (advantages / disadvantages) between both 'equivalent' methods

Response: We apologize for the oversight. We have now added it in our manuscript on page 9 with the addition ", as shown previously,". While an in-depth comparison of our method to the vast field of tissue clearing approaches would be beyond the focus of this manuscript, our system is inspired by CLARITY and we aimed to make the process more efficient. Solvent based methods like iDISCO can take somewhat longer. We are not claiming to be the first to image amyloid in cleared tissue. Such work was even published before Liebmann et al 2016, for example, by Jährling et al 2015. None of these work compared therapeutic outcomes, which is the main point of our work.

6.) The results on effect sizes are interesting. However, it could be helpful besides providing p-

values to also provide actual variation of counts (std) encountered in e.g., wild type vs treatment groups in various areas. I.e., are certain areas highly variable in plaque expression in untreated mice already. Which areas show highest variation? If there are strong differences in variation what are possible reasons for it.

Response: We thank the Reviewer for this suggestion. The fold change plots in Figures S7, S9, and S10 depict the variability at the regional level for all groups, while the various control heatmaps in Fig. S6 also provide a preview into the average values for each cohort at the voxel-level. However, we agree that this would be useful data to share within our study, and we have added a new Supplementary Figure S15 depicting the regional variability in each plaque metric per group.

7.) It could be interesting to include a combined LIN5044 and NB360 treatment into the study to see if those complement each other or actually might interact in non-linear ways in certain areas.

Response: We thank the Reviewer for this interesting suggestion.

As depicted in Fig. 4, the two highly effective treatments, NB360 and LIN5044, affected mean plaque size in almost completely distinct regions of the brain. Based on this result, we agree that evaluating a cocktail of these two drugs would be an interesting experiment to carry out. However, since NB360 was primarily effective at 5-months, and LIN5044 at 14-months, evaluating their combination based on this result would require doubling the treatment duration from 90 days to 180 days. In this study, all treatment groups received their drug for 90 days. We believe that modifying this would require significant optimization beyond the scope of our study, which mainly focused on the evaluation of existing treatments and doses.

More generally, as our study explores treatment efficacy across every permutation of three drugs, two timepoints, and three plaque metrics in an unbiased manner, we believe that introducing an additional axis of exploration, specifically combinations of treatments, would be an exponentially larger scope. Nonetheless, we agree that experiments regarding cocktail therapies are wonderful directions of future studies that would be guided by the current results.

Reviewer #1 (Significance (Required)):

see report above for a closely related earlier study not discussed in the manuscript. a comparison between both methods is needed.

The results of the study particular alignment of the effect sizes with transcriptomics data is very interesting as it allows to generate hypothesis on the underlying mechanisms.

for more details see report above.

Response: We thank the Reviewer for this extremely helpful feedback. As discussed in point 5, we have updated our manuscript and included a recent review (Richardson et al 2021) of tissue clearing methods to our discussion on page 9.

Reviewer #2 (Evidence, reproducibility and clarity (Required)):

In this manuscript, Kirscherbaum et al described 3 major science advance they accomplished:
1, Developed a novel method to globally evaluate amyloid plaque load across brain of an amyloidosis model with high resolution.
2, Compared spatial changes of amyloid plaque density, size and maturity after treatment of 3 different anti-amyloid molecules.
3, Analyzed spatial correlation between gene expression and amyloid plaque changes.

The findings are novel and interesting (most data were published in a biorxiv last year). I support publication of the manuscript after the authors address several of my points.

Major points:
1, The author use tissue clearing and qFTAA/hFTAA labeling of whole brain for plaque labeling. That is the basis of the whole manuscript. How does the labeling of qFTAA and hFTAA comparing with widely used methoxy-X04 or X34 labeling of plaques? Methoxy X04 is BBB penetrant and widely used to label dense core plaques (Klunk et al 2002 PMID: 12230326). It will be good for the authors to compare their method with methoxy labeling.

Response: The Authors thank the Reviewer for this feedback. Polythiophenes are well established amyloid dyes which were described in detail in many papers cited in the manuscript [14,15]. We chose to use Polythiophenes since they allow distinction between amyloid conformations, which is a major advantage of these molecules. We also show in our manuscript how polythiophene staining relates to labeling by a set of amyloid-beta antibodies which target specific amyloid-beta species. These results further underscored the versatility of polythiophene dyes.

2, the authors tried to make a point that whole brain electrophoretic staining with polythiophene can label plaque equally comparing with traditional staining. However, the two panels in figure 1D showed changes of several plaques either by location or by intensity (lower right, middle left etc) which is concerning in term of consistency of the electrophoresis labeling. Based on numbers in the manuscript, the change is minimal between the two conditions, but the picture showed different morphology or number for at least a few percent of the plaques. I would like the authors do more analysis and show a few more pictures in a supplementary figure.

Response: We thank the Reviewer for raising this point. As this is similar to Point 2 of Reviewer #1 and would like to refer to that response:

This calibration experiment was originally a qualitative test, and we found that the difference between the images was minimal by simple visual inspection. However, the re-staining, re-mounting, and re-focusing can result in distortions of the tissue. To better evaluate this, we have included an additional supplementary figure with more images and quantitative comparisons (see Fig. S3). We also emphasized this in the text: "Slight differences in plaque counts and morphology were a result of physical distortions of the slices during passive staining, and due to focal shifts during re-imaging of the free-floating slices." (p.4).

3, the lack of obvious plaque changes in mice after 3m antibody treatment is a little surprising since many papers showed immunotherapy is able to reduce amyloid plaque load (antibodies like 3D6, mE8). The authors evaluated PK but did not discuss extensively the dose they select. What is the basis for that dose? One thing can be done is a target engagement study with biotinylated antibody. The authors know there is exposure of the antibody in the tissue but they did not test whether the antibody engage with Abeta.

Response: We agree that the low impact (however, not completely absent) of the beta-1 antibody on the amyloid load is surprising. However, as we discuss in our manuscript (p. 9), the same antibody was used in two publications (Balakrishnan et al. 2015, Pfeifer et al. 2002). One paper found no difference in amyloid load (Balakrishnan et al 2015), while the other paper did see a change in amyloid load (Pfeifer et al 2002). We chose the same dose as was used in these two papers. Balakrishnan et al 2015 show in their study how amyloid-beta epitopes are masked by the *in vivo* injected beta-1 antibody – proving that the antibody penetrates the blood brain barrier and binds to amyloid. To address this comment, we have added to the result section: ", based on previous protocols (Balakrishnan et al 2015, Pfeifer et al 2002)". We have also added to the discussion: "It was shown that Beta-1 injection results in target engagement (Balakrishnan et al 2015)".

4, the spatial correlation of gene expression and plaque change is very intriguing and potentially important to understand brain microenvironment. But I am not sure the data fully make sense. For example, the BACE1 mRNA is mainly in the cytoplasm but BACE1 protein is often transported and localized into neurites. Does that mean the cell body BACE is the main driver for Abeta production? if not, the spatial correlation will be difficult to explain.

Response: This is a great a point. We agree that gene expression and final protein expression are not necessarily spatially colocalized. This would need to be taken into consideration when using our therapy atlas to generate new hypotheses. In our case, we show that there is a good correlation between BACE1 mRNA expression and the effect of the BACE1 inhibitor. This does not exclude that we miss some areas where the effect of BACE1 inhibition unfolds in remote synapses. We suspect this could be cell-type dependent (e.g. depending on interneurons, projection neurons). In order to discuss this point further we added to the discussion the following sentence on p.11: "Correlating spatial transcriptomic atlases to therapy atlases as shown here, can be used as a tool to generate hypotheses. When using such an approach one needs to take into consideration that gene expression and protein localization might not coincide – adding complexity to data interpretation."

Minor points:

1, Several figures (e.g. Figure 2F) is difficult to read with small font and panel.

2, Figure 2D can be put in supplemental.

3, The difference of LIN5044's impact on intracellular prion protein and extracellular abeta can be discussed a bit more.

Response: We think the mechanism of action of LIN5044 is very similar in amyloid-beta and prion models. LIN5044 was shown to stabilize extracellular prion fibrils and by that reduce their templating potential. We think it is by the same mechanism through which LIN5044 inhibits the growth of amyloid-beta plaques. To clarify this we added to the discussion: "In prion diseases, polythiophenes (including LIN5044) slow disease progression by reducing the number of seeds for secondary nucleation. This is achieved by polythiophenes binding and stabilizing amyloid fibrils, resulting in reduced fibril fragmentation as a source of seeds (14, 15). Similarly, Aβ fibril hyper-stabilization could explain the effect of LIN5044 in APP/PS1 mice."

Reviewer #2 (Significance (Required)):

Anti-amyloid therapies have been the front runners for Alzheimer's disease clinical trials, with billions of dollars already spent. It is important to understand the similarities and difference among these therapies. The manuscript tried to address these questions in a comprehensive and age related way in prevention and treatment paradigms. Although I still have some questions and concerns about the methodology, the data package can probably stimulate the broad AD research community to think and design anti-amyloid studies with different disease stages, combination approaches and endpoints in mind. The spatial gene expression analysis is also quite interesting. But the future key experiment will need to done to understand the spatial correlation between mRNA expression and protein distribution in a similar voxel based analysis.

Response: We agree that in the future the transcriptomic analysis will need to be combined with proteomic data. We have added this point to the discussion on p.11: "In the future, such spatial analyses could benefit from incorporating proteomic data."

Reviewer #3 (Evidence, reproducibility and clarity (Required)):

Summary:
The authors developed an improved tissue clearing method based on a focused electrophoretic clearing. They used this method to analyze the effects of anti Aß immunotherapy (ß1 antibody), BACE1 inhibition (NB360), and amyloid intercalator (LIN5044) treatments in young or old APP/PS1 mice. Effects on amyloid plaque density, structure (presence of a dense core) and size were studied.
The main conclusions were that the immunotherapy had no effect. The BACE1 antagonist decreased the number and size of plaques but only when applied to young mice. The amyloid-

binding compound LIN5044 did not change plaque number but increased their compaction when applied in old mice.
Alignment with published spatial transcriptomic indicated that the effect of LIN5044 on plaque compaction occurred in association with expression of Cx3c11, a microglial chemoattractant mostly expressed by neurons. Accordingly, the authors found that areas where LIN5044 has a stronger effect had more microglia.

Major comments:
In the Abstract, Introduction, ... the authors argue that clinical trial failures of Aß-therapies could be due to the fact that specific treatments may have regional or temporal heterogeneity of efficacy. However, Aß immunotherapy is very efficient to remove plaques in human patients, even so it does not prevent cognitive deterioration. Thus, the absence of clinical effects is not due to a poor efficiency for plaque removal. This argument should be removed from all the manuscript. Further, BACE antagonist therapy were stopped because of side effects.

Response: We agree that some Abeta immunotherapies are potently removing amyloid in humans. Although the efficacy is a matter of intense debate, the first and only FDA-approved specific therapy against Alzheimer's disease is an Abeta antibody (adecanumab). As we show, the efficacy of therapies reducing Abeta show temporal and regional heterogeneity in the brain of mice. We argue that this temporal and regional heterogeneity in amyloid clearing is likely to happen in humans too, and that this heterogeneity might have an influence on the outcome of clinical trials. There is no in-depth analysis of the temporal and regional efficacy of Abeta immunotherapy in humans; in fact, our study is the first analysis at such a high resolution in mice. Thus we cannot exclude that the lack of efficacy in humans is due to temporal and neuroanatomical heterogeneity in drug-effect. Reflecting the Reviewer's concern, we have revised the sentence in the Abstract as follows to: "As observed in this study, there is a striking dependence of specific treatments on the location and maturity of Aβ plaques. This may also contribute to the clinical trial failures of Aβ-therapies…"

As it is known that immunotherapy against Aß has strong effects on plaques, both in human AD patients and in transgenic mouse models, the surprisingly modest (at best) effect of immunotherapy in the present study was likely a technical issue. Thus, the data concerning Aß immunotherapy should be removed.

Response: We thank the Reviewers for raising this important point. The beta-1 antibody used in this study has been used in two previous studies [31,32]. Balakrishnan et al. 2015 [32] demonstrated that this beta1-antibody treatment reduced pathology in 5-month-old APP mice, whereas treatment with 7-month-old mice yielded no noticeable effect on pathology. We noticed similar results with our whole brain quantitative methods, in that the beta1-antibody was significantly more effective in younger 5-month-old mice than in 14-month-old mice (Fig. S7, S8). In summary, although the beta1-antibody treatment effect is surprisingly modest compared to the other treatments we explored (NB360 and LIN5044), the effect of the beta1-antibody we observed is in accordance with the literature.

Review COMMONS

In the quantifications the authors frequently indicated the significance of the results without showing the magnitude of the effects (Fig. 2). When the fold changes were presented (Suppl Fig 6, 8 and 9), there was no indication of the relative level of plaque parameters according to the various brain regions analyzed. Further, in Fig 2 as in Suppl Fig 6, 8 and 9 there were many samples per region, and the borders between regions were not indicated. I would suggest to make graphs with the mean values per region, to allow an easier visualization of the effects.

Response: We thank the Reviewers for raising this important point. We agree that it is important to visualize the relative levels of plaque parameters for each region in addition to the fold change. We have added an additional supplementary figure (S15) with graphs of the mean values per region to allow for better visualization.

Minor comments:
Suppl Fig 12: was it really n=1 as indicated in the Legend? If so, it should be removed. Also, it is not clear why no difference in Aß abundance was detected between the 8 brain regions (Results p. 8), given the major differences in plaque density.

Response: We have removed the n=1 in the legend for Fig S13 (originally S12) and have updated the legend text to better indicate the various groups. When quantifying plaque density and drug efficacy for plaque reduction, we mainly detected differences at the neuroanatomical region and voxel level (Fig. 2G,3A). However, as demonstrated in Fig. 2F, the effects across the whole brain were much less drastic than across regions or voxels (Fig. 2G, 3A). Similarly, we suspect that pooling brain tissue into large chunks before processing will mask most of these region-specific results. We would also like to highlight that western blots may not be ideal for quantitative measurements.

In the Discussion p. 9, it is indicated that "... both LIN5044 and NB360 cleared plaques ...". However, in Fig 2 LIN5044 did not modify plaque density.

Response: We would like to apologize for this oversight. We have replaced “cleared plaques” to “reduced amyloid load”.

The mention of ventrocaudal and dorsorostral gradients should be removed. Indeed, ventral and caudal (or dorsal and rostral) regions are eventually co-localized, and it is therefore not possible to establish a gradient between them.

p. 10 "... evidence that LIN5044 reduces additive plaque growth". Is it possible that LIN5044 increases plaque compaction?

Response: This is a great point. We have added the following to the discussion: “Potentially, smaller plaques might be a consequence of an increase in plaque compaction upon LIN5044.”

Review COMMONS

Methods p. 12 Tissue clearing. Clearing time varied between 6 and 14 hours. What were the criteria to stop the clearing?

Response: We have updated the methods section to mention this: "Tissue clarity was determined by visual inspection."

The color codes for increasing or decreasing effects are opposite in Figs 2 and 3.

Legend of Suppl Fig 4: It seems to me that the "C, " in the various parentheses should be removed.

Response: We have removed "C" from the parentheses.

Reviewer #3 (Significance (Required)):

The study is very interesting and brings new methodological tools. Since the failure of several clinical trials in Alzheimer's disease targeting the amyloid pathology, it remains important to understand the reasons of these failures. Here the authors bring new hypothesis regarding the maturity of amyloid deposits, their localization and specific treatments.

Response: We thank the Reviewer for the encouragement and positive feedback.

21st Sep 2022

Dear Prof. Lee,

Thank you for the submission of your revised manuscript to EMBO Molecular Medicine. I am pleased to inform you that we will be able to accept your manuscript pending the following final amendments:

1) Please address the referee #1 (Review Commons referee #2) point about target engagement experimentally. All other points from both referees should be addressed in writing.
2) Your manuscript is currently being reviewed by our data editors. I will send you the manuscript with the track changes suggested by our data editors as soon as all the checks are completed.
3) Source data: During a standard image analysis we detected potential aberrations in the figure set, and we would like to clarify these issues before sending your paper back to referees. We kindly invite you to check the composition of Figures 2B and Supplementary Figure S11A yourself, and to send us the related source data. If you make changes to the figure, please include a point-by-point describing what you changed.

Image source data should be provided as one file per figure that contains the original, uncropped and unprocessed scans of all or key gels/microscopy images used in the figure. The file(s) should be labeled with the appropriate figure/panel number, and should display molecular weight markers; further annotation may be useful but is not essential. Source data files will be published online with the article as supplementary "Source Data."
4) Manuscript format: Please format your manuscript according to our guidelines outlined here:
https://www.embopress.org/page/journal/17574684/authorguide#researcharticleguide
5) Please submit a complete Author Checklist. https://www.embopress.org/pb-assets/embo-site/EMBO%20Press%20Author%20Checklist-1642513524327.xlsx
6) Figures: Please upload individual, high-resolution main Figure files. Please check "Author Guidelines" for more information:
https://www.embopress.org/page/journal/17574684/authorguide#figureformat
7) In the main manuscript file, please do the following:
- Add up to 5 keywords.
- All Figures should be called out in a sequential order. Currently Fig. S8 is called out after Fig S10, please correct. Also, as there is only one panel in Figure 3 it should be simply called out as Fig 3 and panel label removed.
- Please rename "Competing Interest" to "Disclosure Statement & Competing Interests". We updated our journal's competing interests policy in January 2022 and request authors to consider both actual and perceived competing interests. Please review the policy https://www.embopress.org/competing-interests and update your competing interests if necessary. Also, please add the sentence "Prof. Adriano Aguzzi is a member of the EMM Editorial Board. This has no bearing on the editorial consideration of this article for publication."
- Correct the reference citation in the reference list and remove URLs. Where there are more than 10 authors on a paper, 10 will be listed, followed by "et al.". Please check "Author Guidelines" for more information.
https://www.embopress.org/page/journal/17574684/authorguide#referencesformat
- In data availability statement if no data are deposited in public repositories, please add the sentence: "This study includes no data deposited in external repositories". Computational code should, however, be deposited in an appropriate repository and made freely available.

Please check "Author Guidelines" for more information.
https://www.embopress.org/page/journal/17574684/authorguide#availabilityofpublishedmaterial
8) Appendix: Supplementary tables and supplementary figures and their legends should be merged into one PDF labelled "Appendix". Ideally, figure legends should be displayed underneath the corresponding figure. A table of contents should be added with page numbers. Please rename all supplementary figures and tables to "Appendix Figure S1" etc and "Appendix Table S1" etc and update their callouts in the main manuscript text.
9) Funding. Please merge this section with "Acknowledgments".
10) Author contributions: Please remove it from the manuscript and specify author contributions in our submission system. CRediT has replaced the traditional author contributions section because it offers a systematic machine-readable author contributions format that allows for more effective research assessment. You are encouraged to use the free text boxes beneath each contributing author's name to add specific details on the author's contribution. More information is available in our guide to authors:
https://www.embopress.org/page/journal/17574684/authorguide#authorshipguidelines
11) The Paper Explained: Please provide "The Paper Explained" and add it to the main manuscript text. Please check "Author Guidelines" for more information. https://www.embopress.org/page/journal/17574684/authorguide#researcharticleguide
12) Synopsis: Every published paper now includes a 'Synopsis' to further enhance discoverability. Synopses are displayed on the journal webpage and are freely accessible to all readers. They include separate synopsis image and synopsis text.
- Synopsis image: Please provide a striking image or visual abstract as a high-resolution jpeg file 550 px-wide x (250-400)-px high to illustrate your article.
- Synopsis text: Please provide a short standfirst (maximum of 300 characters, including space) as well as 2-5 one sentence

bullet points that summarise the paper as a .doc file. Please write the bullet points to summarise the key NEW findings. They should be designed to be complementary to the abstract - i.e. not repeat the same text. We encourage inclusion of key acronyms and quantitative information (maximum of 30 words / bullet point). Please use the passive voice.
- Please check your synopsis text and image before submission with your revised manuscript. Please be aware that in the proof stage minor corrections only are allowed (e.g., typos).
13) For more information: This space should be used to list relevant web links for further consultation by our readers. Could you identify some relevant ones and provide such information as well? Some examples are patient associations, relevant databases, OMIM/proteins/genes links, author's websites, etc...
14) As part of the EMBO Publications transparent editorial process initiative (see our Editorial at http://embomolmed.embopress.org/content/2/9/329), EMBO Molecular Medicine will publish online a Review Process File (RPF) to accompany accepted manuscripts. This file will be published in conjunction with your paper and will include the anonymous referee reports, your point-by-point response and all pertinent correspondence relating to the manuscript. Let us know whether you agree with the publication of the RPF and as here, if you want to remove or not any figures from it prior to publication. Please note that the Authors checklist will be published at the end of the RPF.
15) Please provide a point-by-point letter INCLUDING my comments as well as the reviewer's reports and your detailed responses (as Word file).

I look forward to reading a new revised version of your manuscript as soon as possible.

Yours sincerely,

Zeljko Durdevic

Zeljko Durdevic
Editor
EMBO Molecular Medicine

*** Instructions to submit your revised manuscript ***

*** PLEASE NOTE *** As part of the EMBO Publications transparent editorial process initiative (see our Editorial at https://www.embopress.org/doi/pdf/10.1002/emmm.201000094), EMBO Molecular Medicine will publish online a Review Process File to accompany accepted manuscripts.

In the event of acceptance, this file will be published in conjunction with your paper and will include the anonymous referee reports, your point-by-point response and all pertinent correspondence relating to the manuscript. If you do NOT want this file to be published, please inform the editorial office at contact@embomolmed.org.

To submit your manuscript, please follow this link:

https://embomolmed.msubmit.net/cgi-bin/main.plex

When submitting your revised manuscript, please include:

1) a .docx formatted version of the manuscript text (including Figure legends and tables)

2) Separate figure files*

3) supplemental information as Expanded View and/or Appendix. Please carefully check the authors guidelines for formatting Expanded view and Appendix figures and tables at
https://www.embopress.org/page/journal/17574684/authorguide#expandedview

4) a letter INCLUDING the reviewer's reports and your detailed responses to their comments (as Word file).

5) The paper explained: EMBO Molecular Medicine articles are accompanied by a summary of the articles to emphasize the major findings in the paper and their medical implications for the non-specialist reader. Please provide a draft summary of your article highlighting
- the medical issue you are addressing,

- the results obtained and
- their clinical impact.

This may be edited to ensure that readers understand the significance and context of the research.
Please refer to any of our published articles for an example.

6) For more information: There is space at the end of each article to list relevant web links for further consultation by our readers. Could you identify some relevant ones and provide such information as well? Some examples are patient associations, relevant databases, OMIM/proteins/genes links, author's websites, etc...

7) Author contributions: the contribution of every author must be detailed in a separate section.

8) EMBO Molecular Medicine now requires a complete author checklist (https://www.embopress.org/page/journal/17574684/authorguide) to be submitted with all revised manuscripts. Please use the checklist as guideline for the sort of information we need WITHIN the manuscript. The checklist should only be filled with page numbers were the information can be found. This is particularly important for animal reporting, antibody dilutions (missing) and exact values and n that should be indicted instead of a range.

9) Every published paper now includes a 'Synopsis' to further enhance discoverability. Synopses are displayed on the journal webpage and are freely accessible to all readers. They include a short stand first (maximum of 300 characters, including space) as well as 2-5 one sentence bullet points that summarise the paper. Please write the bullet points to summarise the key NEW findings. They should be designed to be complementary to the abstract - i.e. not repeat the same text. We encourage inclusion of key acronyms and quantitative information (maximum of 30 words / bullet point). Please use the passive voice. Please attach these in a separate file or send them by email, we will incorporate them accordingly.

You are also welcome to suggest a striking image or visual abstract to illustrate your article. If you do please provide a jpeg file 550 px-wide x 400-px high.

10) A Conflict of Interest statement should be provided in the main text

11) Please note that we now mandate that all corresponding authors list an ORCID digital identifier. This takes <90 seconds to complete. We encourage all authors to supply an ORCID identifier, which will be linked to their name for unambiguous name identification.

Currently, our records indicate that the ORCID for your account is 0000-0001-7540-1018.

Please click the link below to modify this ORCID:
Link Not Available

12) The system will prompt you to fill in your funding and payment information. This will allow Wiley to send you a quote for the article processing charge (APC) in case of acceptance. This quote takes into account any reduction or fee waivers that you may be eligible for. Authors do not need to pay any fees before their manuscript is accepted and transferred to our publisher.

*Additional important information regarding Figures

Each figure should be given in a separate file and should have the following resolution:
Graphs 800-1,200 DPI
Photos 400-800 DPI
Colour (only CMYK) 300-400 DPI"

Figures are not edited by the production team. All lettering should be the same size and style; figure panels should be indicated by capital letters (A, B, C etc). Gridlines are not allowed except for log plots. Figures should be numbered in the order of their appearance in the text with Arabic numerals. Each Figure must have a separate legend and a caption is needed for each panel.

*Additional important information regarding figures and illustrations can be found at https://bit.ly/EMBOPressFigurePreparationGuideline. See also figure legend preparation guidelines: https://www.embopress.org/page/journal/17574684/authorguide#figureformat

The system will prompt you to fill in your funding and payment information. This will allow Wiley to send you a quote for the article processing charge (APC) in case of acceptance. This quote takes into account any reduction or fee waivers that you may be eligible for. Authors do not need to pay any fees before their manuscript is accepted and transferred to our publisher.

***** Reviewer's comments *****

Referee #1 (Comments on Novelty/Model System for Author):

In this manuscript, the authors developed a novel method to globally evaluate amyloid plaques load across different brain regions in an amyloidosis model with high resolution. Then the authors compared changes of amyloid plaque density, size and maturity after chronic treatment of 3 different anti-amyloid molecules at two different ages. To properly combine tissue clearing, light-sheet microscopy based plaque imaging and comprehensive quantification package is still novel and important, which deserves to be seen by Alzheimer's and imaging field. The different patterns of plaque change upon treatment of these molecules are also intriguing. Although the discussion to explain these observations is not fully convincing, that is probably acceptable for a short article.

Referee #1 (Remarks for Author):

The modified manuscript discussed my concerns of poor target engagement in the antibody therapy arm. But an engagement study is really easy and the authors could have done a simple staining to show the potentially diluted target engagement.

The original Pfeifer 2002 paper showed hemorrhage induced by the same antibody. The authors could also analyze or at least discuss changes of vascular plaque vs parenchymal plaque.

Referee #2 (Comments on Novelty/Model System for Author):

The study brings new methodological tools (improved tissue clearing method based on a focused electrophoretic clearing) that are applied to understand why several clinical trials in Alzheimer's disease targeting the amyloid pathology have failed. The technique is here validated using classical histology study on brain slices. Based on the use of this new technology, the authors bring new hypothesis regarding the maturity of amyloid deposits, their localization and specific treatments.

Referee #2 (Remarks for Author):

The revised version of the manuscript from Kirschenbaum has now addressed the comments of this reviewer. Still some revisions need to be done in Fig. S15:
Does Fig. S15 show heatmap or hierarchical classification? Are controls and treated properly classified? Fig. S15 legend needs to be updated with a full description of all panels. Only panel 1 is described. The others (mean size & mean maturity) need to be described as B and C.
In the discussion, section Fig. S11 should be replaced with Fig. S12 right?
Finally, the limitations of the study should be highlighted clearly, particularly the translatability to human. Do the authors expect similar results in humans based on the neuropathology studies done after treatment?

We sincerely thank the Reviewers for their helpful comments that helped us significantly improve our revised manuscript and figures.

1) Please address the referee #1 (Review Commons referee #2) point about target engagement experimentally. All other points from both referees should be addressed in writing.

Response: We agree with the Reviewer that this would be a nice addition to the study. However, this antibody has been previously used *in vivo* in more than three peer reviewed publications we have cited. These three papers show intracerebral and peripheral effects on amyloid load with the β1 antibody. Balakrishnan *et al.* (2015) showed epitope masking on the plaques (see their Fig. 3), and immunoprecipitated antibody-bound Aβ (see their Fig. 6). In Pfeifer *et al.* (2002) there is a clear reduction of Aβ histologically and biochemically in the brain. In Winkler *et al.* (2010) the authors demonstrate that the β1 antibody binds Aβ plaques and also retains Aβ in the blood in vivo – we have updated our manuscript to also include this reference. It is important to note that the therapeutic effect of Aβ antibodies can emerge not only from direct plaque-binding in the brain, but also from the Aβ "sink" effect changing Aβ concentration in the plasma (DeMattos *et al.*, 2001). Lastly, all in-depth analyses and the main conclusions in this study are based on the effects of a BACE1 inhibitor and a polythiophene compound. The effectiveness of the β1 antibody is not a primary focus of our study. We show how the BACE1 inhibitor and the polythiophene result in spatially distinct effects. These conclusions won't change regardless of diluted antibody target-engagement *in vivo*. Hence, we argue that doing this experiment will take significantly more time than is relevant to the conclusion of our study, while it was previously shown that the β1-antibody reaches the brain parenchyma and engages Aβ in the plasma and the CNS. We have further discussed this point in the Discussion as follows:

> **Page 9, Line 27:** "It was shown that peripheral β1 injections result in significant Aβ reduction in the brain and Aβ epitope masking (Pfeifer, Boncristiano et al. 2002, Balakrishnan, Rijal Upadhaya et al. 2015). Furthermore, the β1 antibody was shown to bind Aβ plaques in the brain and to retain Aβ in the blood after peripheral administration (Winkler, Abramowski et al. 2010). However, the limited effect of the β1 antibody may be explained by its low bioavailability relative to the high abundance of Aβ."

Below, we have included results from a calibration with the β1 antibody on APPPS1 brain slices. Panel A depicts staining with β1 conjugated to a DY677 fluorophore, and Panel B depicts staining without an antibody, clearly showing β1 staining of the plaques (scale bars represent 100 um).

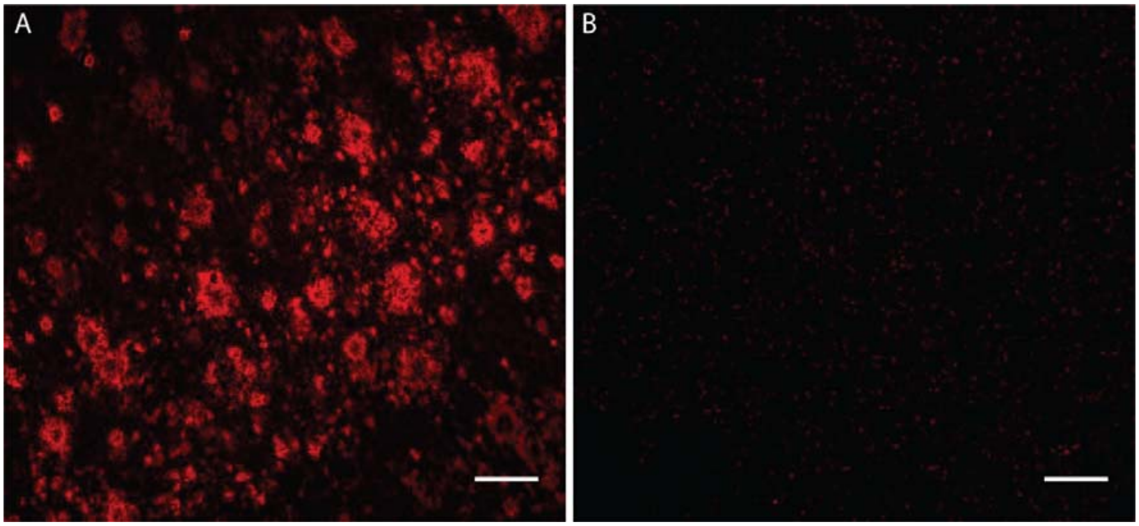

2) Your manuscript is currently being reviewed by our data editors. I will send you the manuscript with the track changes suggested by our data editors as soon as all the checks are completed.

Response: The Authors would like to thank the Data Editors for their comments. As indicated in the tracked changes document, we have updated the legend for Figure 4 to include the number of replicates used in the statistical analysis:

> **Page 31, Line 9:** (*p=0.03; hypergeometric test: 32403808 total voxels, 66128 NB360 voxels, 492779 LIN5044 voxels, 947 overlapping voxels).

3) Source data: During a standard image analysis we detected potential aberrations in the figure set, and we would like to clarify these issues before sending your paper back to referees. We kindly invite you to check the composition of Figures 2B and Supplementary Figure S11A yourself, and to send us the related source data. If you make changes to the figure, please include a point-by-point describing what you changed.

Response: Old Figure 2B and S11A, which is now Appendix Figure S7 in the revised manuscript, illustrate how an image looks before and after segmentation. In Figure 2B, the image gives the reader an illustration of this process, while in the old Figure S11A the image helps explain the rest of that figure. These two images are identical. We agree that reusing the same image can be confusing. We have removed this panel in old Figure S11A entirely. Instead, where we previously introduced Figure S11 in the text, we now refer to Figure 2B.

> **Page 7, Line 11:** "Upon segmentation, we color-coded plaques based on their size (Appendix Figure S7, 2B)."

Image source data should be provided as one file per figure that contains the original, uncropped and unprocessed scans of all or key gels/microscopy images used in the figure. The file(s) should be labeled with the appropriate figure/panel number, and should display molecular weight markers; further annotation may be useful but is not essential. Source data files will be published online with the article as supplementary "Source Data."

Response: We have included source files for the confocal and stereomicroscopy images (Fig 1, EV1, and Appendix Figure S3). The 3D light-sheet datasets are in the range of 100 terabytes and cannot be efficiently summarized in 2D images. We have archived all datasets on external hard disks and they are available upon request. We show gels and western blots in 2 figures (Appendix Figures S2 and S9). All gels and blots, and the photographs thereof, are shown in these figures in their original, non-cropped form.

4) Manuscript format: Please format your manuscript according to our guidelines outlined here: https://www.embopress.org/page/journal/17574684/authorguide#researcharticleguide

Response: This has been completed.

5) Please submit a complete Author Checklist.
https://www.embopress.org/pb-assets/embo-site/EMBO%20Press%20Author%20Checklist-1642513524327.xlsx

Response: This has been completed.

6) Figures: Please upload individual, high-resolution main Figure files. Please check "Author Guidelines" for more information:
https://www.embopress.org/page/journal/17574684/authorguide#figureformat

Response: This has been completed.

7) In the main manuscript file, please do the following:
- Add up to 5 keywords.

Response: This has been completed.

- All Figures should be called out in a sequential order. Currently Fig. S8 is called out after Fig S10, please correct. Also, as there is only one panel in Figure 3 it should be simply called out as Fig 3 and panel label removed.

Response: This has been completed.

- Please rename "Competing Interest" to "Disclosure Statement & Competing Interests". We updated our journal's competing interests policy in January 2022 and request authors to consider both actual and perceived competing interests. Please review the policy https://www.embopress.org/competing-interests and update your competing interests if necessary.

Response: This has been completed.

Also, please add the sentence "Prof. Adriano Aguzzi is a member of the EMM Editorial Board. This has no bearing on the editorial consideration of this article for publication."

Response: This has been completed.

- Correct the reference citation in the reference list and remove URLs.

Response: This has been completed.

Where there are more than 10 authors on a paper, 10 will be listed, followed by "et al.". Please check "Author Guidelines" for more information.
https://www.embopress.org/page/journal/17574684/authorguide#referencesformat

Response: This has been completed.

- In data availability statement if no data are deposited in public repositories, please add the sentence: "This study includes no data deposited in external repositories". Computational code should, however, be deposited in an appropriate repository and made freely available.

Please check "Author Guidelines" for more information.
https://www.embopress.org/page/journal/17574684/authorguide#availabilityofpublishedmaterial

Response: This has been completed.

8) Appendix: Supplementary tables and supplementary figures and their legends should be merged into one PDF labelled "Appendix". Ideally, figure legends should be displayed underneath the corresponding figure. A table of contents should be added with page numbers. Please rename all supplementary figures and tables to "Appendix Figure S1" etc and "Appendix Table S1" etc and update their callouts in the main manuscript text.

Response: This has been completed.

9) Funding. Please merge this section with "Acknowledgments".

Response: This has been completed.

10) Author contributions: Please remove it from the manuscript and specify author contributions in our submission system. CRediT has replaced the traditional author contributions section because it offers a systematic machine-readable author contributions format that allows for more effective research assessment. You are encouraged to use the free text boxes beneath each contributing author's name to add specific details on the author's contribution. More information is available in our guide to authors:
https://www.embopress.org/page/journal/17574684/authorguide#authorshipguidelines

Response: This has been completed.

11) The Paper Explained: Please provide "The Paper Explained" and add it to the main manuscript text. Please check "Author Guidelines" for more information.
https://www.embopress.org/page/journal/17574684/authorguide#researcharticleguide

Response: This has been completed.

12) Synopsis: Every published paper now includes a 'Synopsis' to further enhance discoverability. Synopses are displayed on the journal webpage and are freely accessible to all readers. They include separate synopsis image and synopsis text.

- Synopsis image: Please provide a striking image or visual abstract as a high-resolution jpeg file 550 px-wide x (250-400)-px high to illustrate your article.
- Synopsis text: Please provide a short standfirst (maximum of 300 characters, including space) as well as 2-5 one sentence bullet points that summarise the paper as a .doc file. Please write the bullet points to summarise the key NEW findings. They should be designed to be complementary to the abstract - i.e. not repeat the same text. We encourage inclusion of key acronyms and quantitative information (maximum of 30 words / bullet point). Please use the passive voice.
- Please check your synopsis text and image before submission with your revised manuscript. Please be aware that in the proof stage minor corrections only are allowed (e.g., typos).

Response: This has been completed.

13) For more information: This space should be used to list relevant web links for further consultation by our readers. Could you identify some relevant ones and provide such information as well? Some examples are patient associations, relevant databases, OMIM/proteins/genes links, author's websites, etc...

Response: This has been completed.

14) As part of the EMBO Publications transparent editorial process initiative (see our Editorial at http://embomolmed.embopress.org/content/2/9/329), EMBO Molecular Medicine will publish online a Review Process File (RPF) to accompany accepted manuscripts. This file will be published in conjunction with your paper and will include the anonymous referee reports, your point-by-point response and all pertinent correspondence relating to the manuscript. Let us know whether you agree with the publication of the RPF and as here, if you want to remove or not any figures from it prior to publication. Please note that the Authors checklist will be published at the end of the RPF.

Response: Authors agree.

15) Please provide a point-by-point letter INCLUDING my comments as well as the reviewer's reports and your detailed responses (as Word file).

Response: This has been completed.

I look forward to reading a new revised version of your manuscript as soon as possible.

*** Instructions to submit your revised manuscript ***

*** PLEASE NOTE *** As part of the EMBO Publications transparent editorial process initiative (see our Editorial at https://www.embopress.org/doi/pdf/10.1002/emmm.201000094), EMBO Molecular Medicine will publish online a Review Process File to accompany accepted manuscripts.

In the event of acceptance, this file will be published in conjunction with your paper and will include the anonymous referee reports, your point-by-point response and all pertinent correspondence relating to the manuscript. If you do NOT want this file to be published, please inform the editorial office at contact@embomolmed.org.

Response: This has been completed. We agree to publish the Review Process File.

To submit your manuscript, please follow this link:
https://embomolmed.msubmit.net/cgi-bin/main.plex

When submitting your revised manuscript, please include:

1) a .docx formatted version of the manuscript text (including Figure legends and tables)

Response: This has been completed.

2) Separate figure files*

Response: This has been completed.

3) supplemental information as Expanded View and/or Appendix. Please carefully check the authors guidelines for formatting Expanded view and Appendix figures and tables at
https://www.embopress.org/page/journal/17574684/authorguide#expandedview

Response: This has been completed.

4) a letter INCLUDING the reviewer's reports and your detailed responses to their comments (as Word
file).

Response: This has been completed.

5) The paper explained: EMBO Molecular Medicine articles are accompanied by a summary of the articles to emphasize the major findings in the paper and their medical implications for the non-specialist reader. Please provide a draft summary of your article highlighting
- the medical issue you are addressing,
- the results obtained and
- their clinical impact.
This may be edited to ensure that readers understand the significance and context of the research. Please refer to any of our published articles for an example.

Response: This has been completed.

6) For more information: There is space at the end of each article to list relevant web links for further

consultation by our readers. Could you identify some relevant ones and provide such information as well? Some examples are patient associations, relevant databases, OMIM/proteins/genes links, author's websites, etc...

Response: This has been completed.

7) Author contributions: the contribution of every author must be detailed in a separate section.

Response: This has been completed.

8) EMBO Molecular Medicine now requires a complete author checklist (https://www.embopress.org/page/journal/17574684/authorguide) to be submitted with all revised manuscripts. Please use the checklist as guideline for the sort of information we need WITHIN the manuscript. The checklist should only be filled with page numbers were the information can be found. This is particularly important for animal reporting, antibody dilutions (missing) and exact values and n that should be indicted instead of a range.

Response: This has been completed.

9) Every published paper now includes a 'Synopsis' to further enhance discoverability. Synopses are displayed on the journal webpage and are freely accessible to all readers. They include a short stand first (maximum of 300 characters, including space) as well as 2-5 one sentence bullet points that summarise the paper. Please write the bullet points to summarise the key NEW findings. They should be designed to be complementary to the abstract - i.e. not repeat the same text. We encourage inclusion of key acronyms and quantitative information (maximum of 30 words / bullet point). Please use the passive voice. Please attach these in a separate file or send them by email, we will incorporate them accordingly.

You are also welcome to suggest a striking image or visual abstract to illustrate your article. If you do please provide a jpeg file 550 px-wide x 400-px high.

Response: This has been completed.

10) A Conflict of Interest statement should be provided in the main text

Response: This has been completed.

11) Please note that we now mandate that all corresponding authors list an ORCID digital identifier. This takes <90 seconds to complete. We encourage all authors to supply an ORCID identifier, which will be linked to their name for unambiguous name identification.

Currently, our records indicate that the ORCID for your account is 0000-0001-7540-1018.

Please click the link below to modify this ORCID:
https://embomolmed.msubmit.net/cgi-bin/main.plex?el=A7EI1wBV3A7DmtE3Bh4B9ftdJubXLA94OfLOHyPdyOqXQY

Response: This has been completed.

12) The system will prompt you to fill in your funding and payment information. This will allow Wiley to send you a quote for the article processing charge (APC) in case of acceptance. This quote takes into account any reduction or fee waivers that you may be eligible for. Authors do not need to pay any fees before their manuscript is accepted and transferred to our publisher.

*Additional important information regarding Figures

Each figure should be given in a separate file and should have the following resolution:
Graphs 800-1,200 DPI
Photos 400-800 DPI
Colour (only CMYK) 300-400 DPI"

Figures are not edited by the production team. All lettering should be the same size and style; figure panels should be indicated by capital letters (A, B, C etc). Gridlines are not allowed except for log plots. Figures should be numbered in the order of their appearance in the text with Arabic numerals. Each Figure must have a separate legend and a caption is needed for each panel.

*Additional important information regarding figures and illustrations can be found at: https://bit.ly/EMBOPressFigurePreparationGuideline.

See also figure legend preparation guidelines:
https://www.embopress.org/page/journal/17574684/authorguide#figureformat

The system will prompt you to fill in your funding and payment information. This will allow Wiley to send you a quote for the article processing charge (APC) in case of acceptance. This quote takes into account any reduction or fee waivers that you may be eligible for. Authors do not need to pay any fees before their manuscript is accepted and transferred to our publisher.

***** Reviewer's comments *****

Referee #1 (Comments on Novelty/Model System for Author):

In this manuscript, the authors developed a novel method to globally evaluate amyloid plaques load across different brain regions in an amyloidosis model with high resolution. Then the authors compared changes of amyloid plaque density, size and maturity after chronic treatment of 3 different anti-amyloid molecules at two different ages. To properly combine tissue clearing, light-sheet microscopy based plaque imaging and comprehensive quantification package is still novel and important, which deserves to be seen by Alzheimer's and imaging field. The different patterns of plaque change upon treatment of these molecules are also intriguing. Although the discussion to explain these observations is not fully convincing, that is probably acceptable for a short article.

Referee #1 (Remarks for Author):

The modified manuscript discussed my concerns of poor target engagement in the antibody therapy arm. But an engagement study is really easy and the authors could have done a simple staining to show the potentially diluted target engagement.

Please see our detailed response to this point under the editorial comment Point 1. The original Pfeifer 2002 paper showed hemorrhage induced by the same antibody. The authors could also analyze or at least discuss changes of vascular plaque vs parenchymal plaque.

Response: The Authors thank the Reviewer for raising this important point. The APP23 model, which was used in the Pfeifer et. al. (2002) study, is known to develop extensive vascular amyloid deposits and show spontaneous microbleeding. However, this is not the case for the APPPS1 model used in our study. See https://www.alzforum.org/research-models/appps1 and https://www.alzforum.org/research-models/app23. The difference of models developing cerebral amyloid angiopathy and bleeding is also addressed in the discussion in Pfeifer et al 2002. Taken together, we think that this point would be only loosely connected to our work and emphasizing it might confuse readers.

Referee #2 (Comments on Novelty/Model System for Author):

The study brings new methodological tools (improved tissue clearing method based on a focused electrophoretic clearing) that are applied to understand why several clinical trials in Alzheimer's disease targeting the amyloid pathology have failed. The technique is here validated using classical histology study on brain slices. Based on the use of this new technology, the authors bring new hypothesis regarding the maturity of amyloid deposits, their localization and specific treatments.

Response: The Authors thank the Reviewer for this succinct summary of our study.

Referee #2 (Remarks for Author):

The revised version of the manuscript from Kirschenbaum has now addressed the comments of this reviewer. Still some revisions need to be done in Fig. S15:
Does Fig. S15 show heatmap or hierarchical classification? Are controls and treated properly classified? Fig. S15 legend needs to be updated with a full description of all panels. Only panel 1 is described. The others (mean size & mean maturity) need to be described as B and C.

Response: The Authors thank the Reviewer for this observation. We would like to confirm that the previous Figure S15 (now Appendix Figure S10) shows hierarchical classification, and that the controls and treated were properly classified. This figure has also been updated to include a full description for each panel.

In the discussion, section Fig. S11 should be replaced with Fig. S12 right?

Response: The Authors thank the Reviewer for noticing this. We sincerely apologize for this mistake; we have replaced Figure S11 with Appendix Figure S8 (Figure S12 in the previous version of the manuscript) in the Discussion.

Finally, the limitations of the study should be highlighted clearly, particularly the translatability to human. Do the authors expect similar results in humans based on the neuropathology studies done after treatment?

Response: We thank the Reviewer for this comment. We have updated the Discussion with the following,

> **Page 11, Line 25**: "However, to what extent regional variation in the effects of CNS drugs translates from mouse to human is unclear. Systematic regional neuropathological analysis after AD clinical trials could shed light on regional differences in drug response."

24th Oct 2022

Dear Prof. Lee,

We are pleased to inform you that your manuscript is accepted for publication and is now being sent to our publisher to be included in the next available issue of EMBO Molecular Medicine.

Please read below for additional IMPORTANT information regarding your article, its publication and the production process.

Congratulations on your interesting work,

Zeljko Durdevic

Zeljko Durdevic
Editor
EMBO Molecular Medicine

Follow us on Twitter @EmboMolMed
Sign up for eTOCs at embopress.org/alertsfeeds

*** *** *** IMPORTANT INFORMATION *** *** ***

SPEED OF PUBLICATION
The journal aims for rapid publication of papers, using using the advance online publication "Early View" to expedite the process: A properly copy-edited and formatted version will be published as "Early View" after the proofs have been corrected. Please help the Editors and publisher avoid delays by providing e-mail address(es), telephone and fax numbers at which author(s) can be contacted.

Should you be planning a Press Release on your article, please get in contact with embomolmed@wiley.com as early as possible, in order to coordinate publication and release dates.

LICENSE AND PAYMENT:

All articles published in EMBO Molecular Medicine are fully open access: immediately and freely available to read, download and share.

EMBO Molecular Medicine charges an article processing charge (APC) to cover the publication costs. You, as the corresponding author for this manuscript, should have already received a quote with the article processing fee separately. Please let us know in case this quote has not been received.

Once your article is at Wiley for editorial production you will receive an email from Wiley's Author Services system, which will ask you to log in and will present you with the publication license form for completion. Within the same system the publication fee can be paid by credit card, an invoice, pro forma invoice or purchase order can be requested.

Payment of the publication charge and the signed Open Access Agreement form must be received before the article can be published online.

PROOFS

You will receive the proofs by e-mail approximately 2 weeks after all relevant files have been sent o our Production Office. Please return them within 48 hours and if there should be any problems, please contact the production office at embopressproduction@wiley.com.

Please inform us if there is likely to be any difficulty in reaching you at the above address at that time. Failure to meet our

deadlines may result in a delay of publication.

All further communications concerning your paper proofs should quote reference number EMM-2022-16789-V2 and be directed to the production office at embopressproduction@wiley.com.

Thank you,

Zeljko Durdevic
Editor
EMBO Molecular Medicine

EMBO Press Author Checklist

Corresponding Author Name: Adriano Aguzzi
Journal Submitted to: EMBO Molecular Medicine
Manuscript Number: EMM-2022-16789

USEFUL LINKS FOR COMPLETING THIS FORM
The EMBO Journal - Author Guidelines
EMBO Reports - Author Guidelines
Molecular Systems Biology - Author Guidelines
EMBO Molecular Medicine - Author Guidelines

Reporting Checklist for Life Science Articles (updated January 2022)
This checklist is adapted from Materials Design Analysis Reporting (MDAR) Checklist for Authors. MDAR establishes a minimum set of requirements in transparent reporting in the life sciences (see Statement of Task: 10.31222/osf.io/9sm4x). Please follow the journal's guidelines in preparing your manuscript.
Please note that a copy of this checklist will be published alongside your article.

Abridged guidelines for figures
1. Data
The data shown in figures should satisfy the following conditions:
- the data were obtained and processed according to the field's best practice and are presented to reflect the results of the experiments in an accurate and unbiased manner.
- ideally, figure panels should include only measurements that are directly comparable to each other and obtained with the same assay.
- plots include clearly labeled error bars for independent experiments and sample sizes. Unless justified, error bars should not be shown for technical replicates.
- if n<5, the individual data points from each experiment should be plotted. Any statistical test employed should be justified.
- Source Data should be included to report the data underlying figures according to the guidelines set out in the authorship guidelines on Data Presentation.

2. Captions
Each figure caption should contain the following information, for each panel where they are relevant:
- a specification of the experimental system investigated (eg cell line, species name).
- the assay(s) and method(s) used to carry out the reported observations and measurements.
- an explicit mention of the biological and chemical entity(ies) that are being measured.
- an explicit mention of the biological and chemical entity(ies) that are altered/varied/perturbed in a controlled manner.
- the exact sample size (n) for each experimental group/condition, given as a number, not a range;
- a description of the sample collection allowing the reader to understand whether the samples represent technical or biological replicates (including how many animals, litters, cultures, etc.).
- a statement of how many times the experiment shown was independently replicated in the laboratory.
- definitions of statistical methods and measures:
  - common tests, such as t-test (please specify whether paired vs. unpaired), simple χ2 tests, Wilcoxon and Mann-Whitney tests, can be unambiguously identified by name only, but more complex techniques should be described in the methods section;
  - are tests one-sided or two-sided?
  - are there adjustments for multiple comparisons?
  - exact statistical test results, e.g., P values = x but not P values < x;
  - definition of 'center values' as median or average;
  - definition of error bars as s.d. or s.e.m.

Please complete ALL of the questions below.
Select "Not Applicable" only when the requested information is not relevant for your study.

Materials

Newly Created Materials	Information included in the manuscript?	In which section is the information available? (Reagents and Tools Table, Materials and Methods, Figures, Data Availability Section)
New materials and reagents need to be available; do any restrictions apply?	Not Applicable	

Antibodies	Information included in the manuscript?	In which section is the information available? (Reagents and Tools Table, Materials and Methods, Figures, Data Availability Section)
For **antibodies** provide the following information:<br>- Commercial antibodies: RRID (if possible) or supplier name, catalogue number and or/clone number<br>- Non-commercial: RRID or citation	Yes	Materials and methods, p16,19

DNA and RNA sequences	Information included in the manuscript?	In which section is the information available? (Reagents and Tools Table, Materials and Methods, Figures, Data Availability Section)
**Short novel DNA or RNA including primers, probes**: provide the sequences.	Not Applicable	

Cell materials	Information included in the manuscript?	In which section is the information available? (Reagents and Tools Table, Materials and Methods, Figures, Data Availability Section)
**Cell lines:** Provide species information, strain. Provide accession number in repository **OR** supplier name, catalog number, clone number, and/**OR** RRID.	Not Applicable	
**Primary cultures:** Provide species, strain, sex of origin, genetic modification status.	Not Applicable	
Report if the cell lines were recently **authenticated** (e.g., by STR profiling) and tested for mycoplasma contamination.	Not Applicable	

Experimental animals	Information included in the manuscript?	In which section is the information available? (Reagents and Tools Table, Materials and Methods, Figures, Data Availability Section)
**Laboratory animals or Model organisms:** Provide species, strain, sex, age, genetic modification status. Provide accession number in repository **OR** supplier name, catalog number, clone number, **OR** RRID.	Yes	Materials and Methods, p13
**Animal observed in or captured from the field:** Provide species, sex, and age where possible.	Not Applicable	
Please detail **housing and husbandry conditions**.	Yes	Materials and Methods, p13

Plants and microbes	Information included in the manuscript?	In which section is the information available? (Reagents and Tools Table, Materials and Methods, Figures, Data Availability Section)
**Plants:** provide species and strain, ecotype and cultivar where relevant, unique accession number if available, and source (including location for collected wild specimens).	Not Applicable	
**Microbes:** provide species and strain, unique accession number if available, and source.	Not Applicable	

Human research participants	Information included in the manuscript?	In which section is the information available? (Reagents and Tools Table, Materials and Methods, Figures, Data Availability Section)
If collected and within the bounds of privacy constraints report on age, sex and gender or ethnicity for all study participants.	Not Applicable	

Core facilities	Information included in the manuscript?	In which section is the information available? (Reagents and Tools Table, Materials and Methods, Figures, Data Availability Section)
If your work benefited from core facilities, was their service mentioned in the acknowledgments section?	Not Applicable	

Design

Study protocol	Information included in the manuscript?	In which section is the information available? (Reagents and Tools Table, Materials and Methods, Figures, Data Availability Section)
If study protocol has been **pre-registered, provide DOI in the manuscript**. For clinical trials, provide the trial registration number **OR** cite DOI.	Not Applicable	
Report the **clinical trial registration number** (at ClinicalTrials.gov or equivalent), where applicable.	Not Applicable	

Laboratory protocol	Information included in the manuscript?	In which section is the information available? (Reagents and Tools Table, Materials and Methods, Figures, Data Availability Section)
Provide DOI OR other citation details if **external detailed step-by-step protocols** are available.	Not Applicable	

Experimental study design and statistics	Information included in the manuscript?	In which section is the information available? (Reagents and Tools Table, Materials and Methods, Figures, Data Availability Section)
Include a statement about **sample size** estimate even if no statistical methods were used.	Yes	Materials and Methods, section: Computational and statistical analysis, p20
Were any steps taken to minimize the effects of subjective bias when allocating animals/samples to treatment (e.g. **randomization procedure**)? If yes, have they been described?	Not Applicable	
Include a statement about **blinding** even if no blinding was done.	Yes	Materials and Methods, section: Whole brain imaging, p19
Describe **inclusion/exclusion criteria** if samples or animals were excluded from the analysis. Were the criteria pre-established?<br><br>If sample or data points were omitted from analysis, report if this was due to attrition or intentional exclusion and provide justification.	Not Applicable	No brain scans were excluded from analysis.
For every figure, are **statistical tests** justified as appropriate? Do the data meet the assumptions of the tests (e.g., normal distribution)? Describe any methods used to assess it. Is there an estimate of variation within each group of data? Is the variance similar between the groups that are being statistically compared?	Yes	Materials and Methods, section: Computational and statistical analysis, p20

Sample definition and in-laboratory replication	Information included in the manuscript?	In which section is the information available? (Reagents and Tools Table, Materials and Methods, Figures, Data Availability Section)
In the figure legends: state number of times the experiment was **replicated** in laboratory.	Yes	Figures, Figure legends, Appendix and Table 1
In the figure legends: define whether data describe **technical or biological replicates**.	Yes	

Ethics

Ethics	Information included in the manuscript?	In which section is the information available? (Reagents and Tools Table, Materials and Methods, Figures, Data Availability Section)
Studies involving **human participants**: State details of **authority granting ethics approval** (IRB or equivalent committee(s), provide reference number for approval.	Not Applicable	
Studies involving **human participants**: Include a statement confirming that **informed consent** was obtained from all subjects and that the experiments conformed to the principles set out in the WMA Declaration of Helsinki and the Department of Health and Human Services Belmont Report.	Not Applicable	
Studies involving **human participants:** For publication of **patient photos**, include a statement confirming that consent to publish was obtained.	Not Applicable	
Studies involving experimental **animals**: State details of **authority granting ethics approval** (IRB or equivalent committee(s), provide reference number for approval. Include a statement of compliance with ethical regulations.	Yes	Materials and Methods, p13
Studies involving **specimen and field samples:** State if relevant **permits** obtained, provide details of authority approving study; if none were required, explain why.	Not Applicable	

Dual Use Research of Concern (DURC)	Information included in the manuscript?	In which section is the information available? (Reagents and Tools Table, Materials and Methods, Figures, Data Availability Section)
Could your study fall under dual use research restrictions? Please check biosecurity documents and list of **select agents and toxins** (CDC): https://www.selectagents.gov/sat/list.htm	Not Applicable	
If you used a select agent, is the security level of the lab appropriate and reported in the manuscript?	Not Applicable	
If a study is subject to dual use research of concern regulations, is the name of the **authority granting approval and reference number** for the regulatory approval provided in the manuscript?	Not Applicable	

Reporting

The MDAR framework recommends adoption of discipline-specific guidelines, established and endorsed through community initiatives. Journals have their own policy about requiring specific guidelines and recommendations to complement MDAR.

Adherence to community standards	Information included in the manuscript?	In which section is the information available? (Reagents and Tools Table, Materials and Methods, Figures, Data Availability Section)
State if relevant guidelines or checklists (e.g., **ICMJE, MIBBI, ARRIVE, PRISMA**) have been followed or provided.	Not Applicable	
For **tumor marker prognostic studies,** we recommend that you follow the **REMARK** reporting guidelines (see link list at top right). See author guidelines, under 'Reporting Guidelines'. Please confirm you have followed these guidelines.	Not Applicable	
For **phase II and III randomized controlled trials**, please refer to the **CONSORT** flow diagram (see link list at top right) and submit the CONSORT checklist (see link list at top right) with your submission. See author guidelines, under 'Reporting Guidelines'. Please confirm you have submitted this list.	Not Applicable	

Data Availability

Data availability	Information included in the manuscript?	In which section is the information available? (Reagents and Tools Table, Materials and Methods, Figures, Data Availability Section)
Have **primary datasets** been deposited according to the journal's guidelines (see 'Data Deposition' section) and the respective accession numbers provided in the Data Availability Section?	Not Applicable	The raw SPIM scans are adding up to ~30-50 terabyte of data; after stitching the size is double. We have the data archived on magnetic tapes. They are available to anyone upon request.
Were **human clinical and genomic datasets** deposited in a public access-controlled repository in accordance to ethical obligations to the patients and to the applicable consent agreement?	Not Applicable	
Are **computational models** that are central and integral to a study available without restrictions in a machine-readable form? Were the relevant accession numbers or links provided?	Not Applicable	
If publicly available data were reused, provide the respective **data citations in the reference list**.	Yes	Results, Materials and Methods, Tables, References
